# Proteomic landscape of TGF-β1-induced fibrogenesis in renal fibroblasts

**DOI:** 10.1038/s41598-020-75989-4

**Published:** 2020-11-04

**Authors:** Shujun Zhou, Xiaoke Yin, Manuel Mayr, Mazhar Noor, Peter J. Hylands, Qihe Xu

**Affiliations:** 1 Renal Science and Integrative Chinese Medicine Laboratory, Department of Inflammation Biology, School of Immunology and Microbial Sciences, King’s College London, London, UK; 2 School of Cardiovascular Medicine and Sciences, King’s BHF Centre of Research Excellence, King’s College London, London, UK; 3 Institute of Pharmaceutical Science, King’s College London, London, UK

**Keywords:** Biochemistry, Cell biology, Computational biology and bioinformatics, Drug discovery, Molecular biology, Systems biology, Kidney diseases

## Abstract

Transforming growth factor-β1 (TGF-β1) plays a premier role in fibrosis. To understand the molecular events underpinning TGF-β1-induced fibrogenesis, we examined the proteomic profiling of a TGF-β1-induced in vitro model of fibrosis in NRK-49F normal rat kidney fibroblasts. Mass spectrometric analysis indicated that 628 cell-lysate proteins enriched in 44 cellular component clusters, 24 biological processes and 27 molecular functions were regulated by TGF-β1. Cell-lysate proteins regulated by TGF-β1 were characterised by increased ribosomal proteins and dysregulated proteins involved in multiple metabolic pathways, including reduced Aldh3a1 and induced Enpp1 and Impdh2, which were validated by enzyme-linked immunosorbent assays (ELISA). In conditioned media, 62 proteins enriched in 20 cellular component clusters, 40 biological processes and 7 molecular functions were regulated by TGF-β1. Secretomic analysis and ELISA uncovered dysregulated collagen degradation regulators (induced PAI-1 and reduced Mmp3), collagen crosslinker (induced Plod2), signalling molecules (induced Ccn1, Ccn2 and Tsku, and reduced Ccn3) and chemokines (induced Ccl2 and Ccl7) in the TGF-β1 group. We conclude that TGF-β1-induced fibrogenesis in renal fibroblasts is an intracellular metabolic disorder and is inherently coupled with inflammation mediated by chemokines. Proteomic profiling established in this project may guide development of novel anti-fibrotic therapies in a network pharmacology approach.

## Introduction

Transforming growth factor (TGF)-β activation of mesenchymal cells, e.g. fibroblasts, plays an important role in fibrosis, a pathological condition characterised by excessive accumulation of extracellular collagen proteins^1^. Fibrosis affects virtually all organs, including the kidney, and causes chronic organ failure and mortality^2^. For example, chronic kidney disease (CKD) affects 8–16% of the world’s population^3,4^. CKD was the 12th most common cause of mortality and caused 4.6% of deaths globally in 2017^5^. If this rising prevalence of CKD continues, it is predicted that CKD will become a top-5 cause of mortality worldwide by 2040^6^. Given that kidney fibrosis is the common pathway mediating CKD progression to end-stage kidney disease, it has long been a burgeoning area of research investigating mechanisms of renal myofibroblasts-mediated fibrogenesis and related intervention^7^.

To facilitate discovery of antifibrotics and mechanistic studies, we have used TGF-β1 to activate fibroblasts and other mesenchymal cells into myofibroblasts and to induce in vitro models of fibrosis suitable for high-throughput screening^8^. An in vitro model of fibrosis induced by TGF-β1 in NRK-49F normal rat kidney fibroblasts has been successfully used to examine antifibrotic and profibrotic activities, many of which have been recapitulated in animal studies and clinical trials^9–12^. Thus, TGF-β1-induced fibrogenesis in NRK-49F cells offers a useful in vitro model for understanding the mechanisms of fibrosis, and for research and development of therapies for preventing and reversing renal fibrosis.

NRK-49F cells are arguably the most commonly used renal fibroblast clones in studies of renal fibrogenesis, and have been used in thousands of scientific reports ever since its first cloning and characterisation in 1978^13^. It was among the earliest renal fibroblasts for studying cellular production and activation of latent TGF-β^14,15^ and has been established as a suitable model to study TGF-β1-induced fibrogenesis in 1998^16^. In this cellular model, many seminal discoveries have been made on TGF-β1-induced fibrogenesis in the past four decades, e.g. TGF-β1 dual regulation of fibroblast proliferation^17^, mutually exclusive nature of TGF-β1-induced proliferation and fibrogenesis in fibroblasts^18^, hepatocyte growth factor and Klotho antagonism to TGF-β1^19,20^ and Smad and non-Smad signal transduction pathways^21–23^.

As far as we know, NRK-49F cells are the only renal fibroblasts in which TGF-β1-induced fibrogenesis has ever been studied by a proteomic approach^24^. In 2009, Kang et al. conducted 2D-gel electrophoresis in-gel digestion, followed by matrix-assisted laser desorption/ionisation-time‐of‐flight mass spectrometry (MALDI-TOF–MS) analysis of selected spots differentially expressed in NRK-49F cells with and without TGF-β1 treatment for 6 h^24^. This study examined cell lysates only, identified 62 TGF-β1-regulated proteins and has established a proof of concept that a proteomics-based approach can be used to investigate mechanisms of fibrogenesis and potential antifibrotic drugs^24^. However, this study has a few major limitations: (i) it did not examine conditioned media, which could provide crucial insights into fibrogenesis, a well-established disorder characterised by the accumulation of collagen in the extracellular compartment; (ii) TGF-β1 treatment for 6 h was not sufficient to induce an established model of fibrosis and indeed, the authors did not show accumulation of total collagens in their samples for proteomic analysis; and (iii) 2D-gel electrophoresis detected more than 1500 protein spots, among which 150 were differentially displayed, but they only selected 78 for proteomic analysis. Thus, they failed to establish an un-biased complete proteomic view on TGF-β1-regulated proteins^24^.

The objective of this study was to apply state-of-the-art proteomic technology to develop an unbiased systems view on intracellular and extracellular events underpinning TGF-β1-induced in vitro model of fibrosis in NRK-49F cells and to identify novel intervening strategies.

## Results

### Establishment of TGF-β1-induced in vitro model of fibrosis

**Figure 1:**
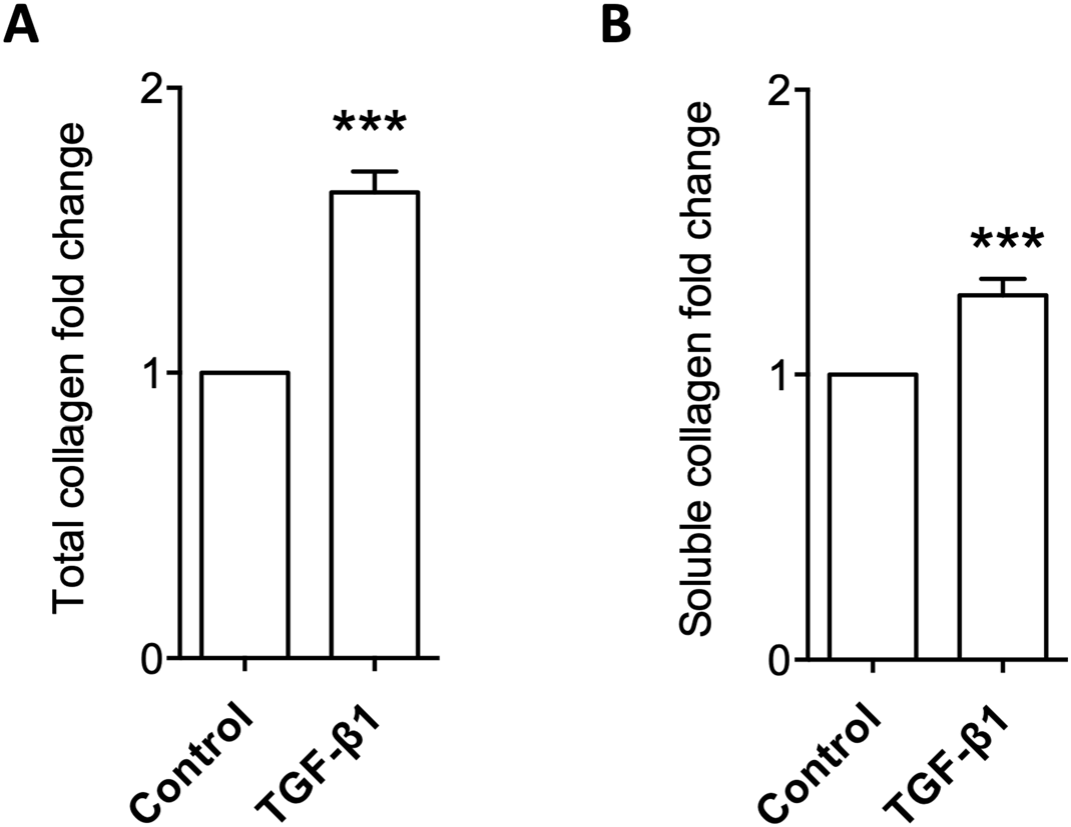
TGF-β1-induced accumulation of total collagen in cell lysates and soluble collagen in conditioned media. NRK-49F cells were cultured with or without 5 ng/ml TGF-β1 for 48 h. Cell lysates and conditioned media were harvested for hydroxyproline assay (**A**) and soluble collagen assay (**B**) to quantify total and soluble collagens, respectively. Data of 4 independent experiments were normalised to the control group of each experiment and shown as mean ± SEM. ***p < 0.001 versus control group.

Quiescent NRK-49F cells were cultured with 5 ng/ml TGF-β1 for 48 h to induce an in vitro model of fibrosis as we reported before^4–7^. Cell lysates and conditioned media of TGF-β1 and control groups of four independent experiments were harvested for measurement of total collagen contents by hydroxyproline assay and soluble collagen assay, respectively. As shown in Fig. 1, TGF-β1 significantly induced total collagen in cell lysates and soluble collagen in conditioned media. The cell lysates and conditioned media were then trypsinised, labelled with tandem mass tag (TMT) reagents and subjected to high performance liquid chromatography (HPLC)-tandem mass spectrometry (MS/MS) analysis.

### Proteomic profiling and enzyme-linked immunosorbent assays (ELISA) of cell lysates

**Figure 2:**
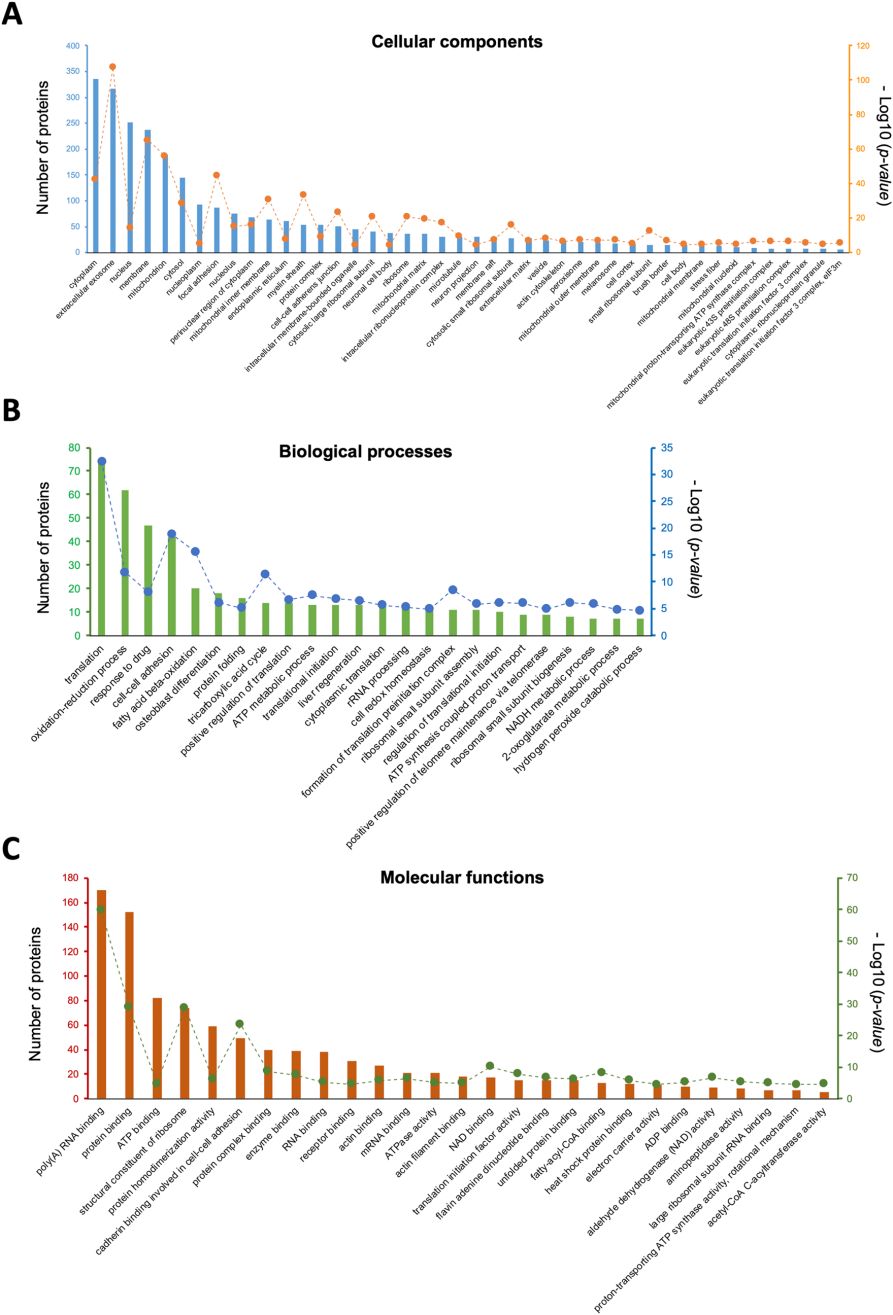
GO analysis of cell-lysate proteins regulated by TGF-β1. Numbers of involved proteins are indicated by the left y-axis and displayed as bars; *p*-values (as -Log10 values) are indicated by the right y-axis and displayed in dots. (**A**) cellular component enrichment; (**B**) biological process enrichment; (**C**) molecular function enrichment.

HPLC–MS/MS analysis identified 1438 proteins with at least 2 unique peptides detected and a 1% false discovery rate (FDR), among which 628 were regulated by TGF-β1 (*p* < 0.05, Supplementary table 1). Gene Ontology (GO) function enrichment analysis of TGF-β1-regulated proteins was performed on Database for Annotation, Visualization and Integrated Discovery (DAVID; https://david.ncifcrf.gov/). As shown in Fig. 2A, TGF-β1-regulated proteins were enriched in 44 GO cellular component clusters (*p* < 0.05, e.g. cytoplasm, extracellular exosome, nucleus, membrane, mitochondrion and cytosol categories), 24 GO biological process clusters (Fig. 2B, e.g. translation, oxidation–reduction process, response to drugs, cell–cell adhesion and fatty acid β-oxidation) and 27 GO molecular function clusters (Fig. 2C, e.g. poly(A)RNA binding, protein binding, ATP binding, structural constituent of ribosome and protein homodimerisation).

**Figure 3:**
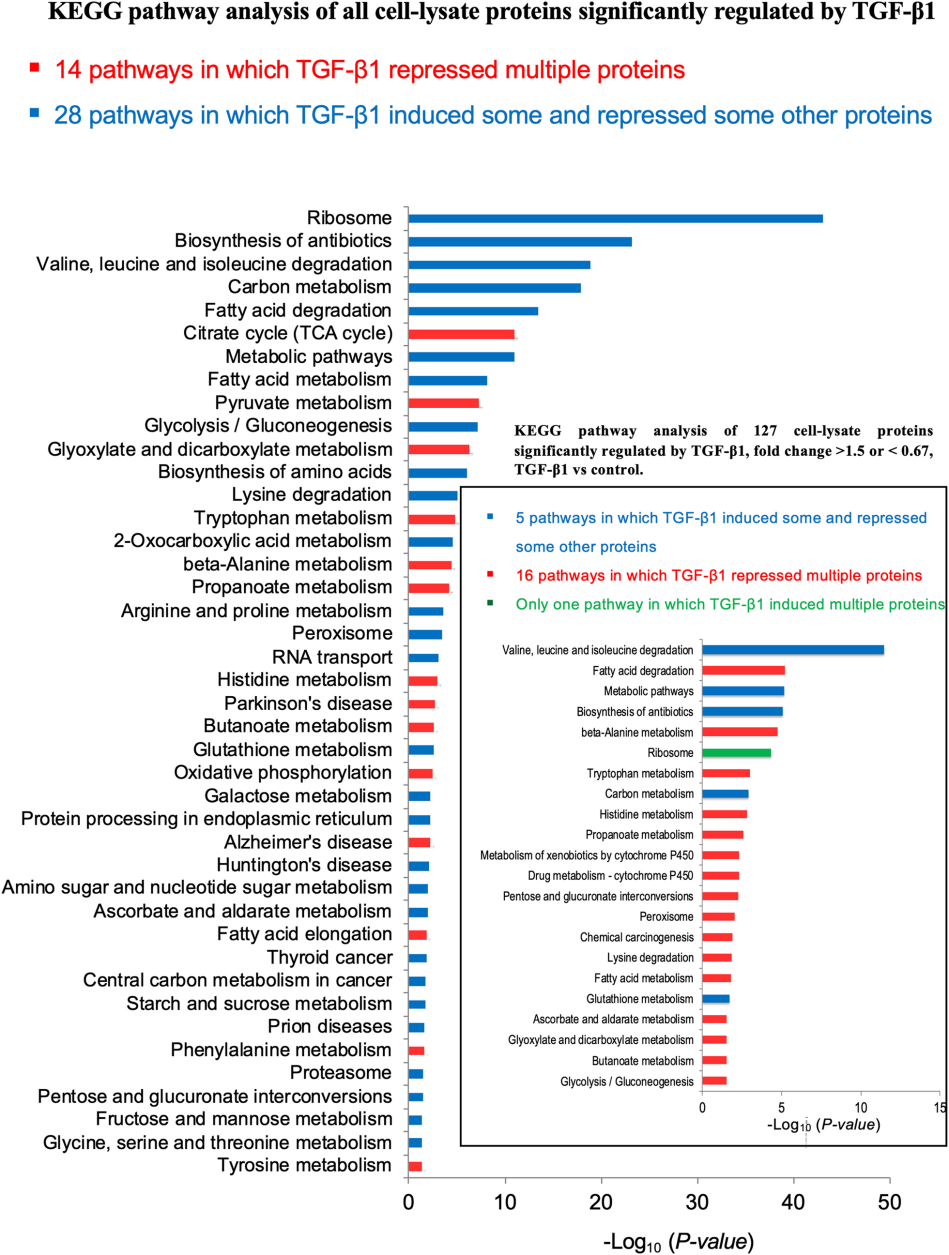
KEGG pathway analyses of cell-lysate proteins regulated by TGF-β1. KEGG analysis of 628 cell-lysate proteins regulated by TGF-β1 shows 42 pathways significantly regulated by TGF-β1. Y-axis represents significantly enriched KEGG pathway terms and X-axis indicates -Log_10_ *p*-value. KEGG pathway analysis of all proteins with TGF-β1/control ratios > 1.5 or < 0.67 is shown in the insert.

KEGG analysis of TGF-β1-regulated proteins using DAVID unveiled 42 enriched pathways (Fig. 3), including 14 pathways in which TGF-β1 repressed multiple proteins and 28 pathways in which TGF-β1 induced some and repressed some other proteins. The proteins involved in each pathway are listed in Supplementary table 2. To focus on the top 20% proteins most robustly regulated by TGF-β1 in terms of fold-change, a cut-off of TGF-β1/Control ratios > 1.5 (n = 51, accounting for 20.08% of all those 254 proteins induced by TGF β1) or < 0.67 (n = 76, accounting for 20.32% of 374 proteins repressed by TGF β1) was applied and the KEGG pathway analysis of short-listed proteins is also shown in Fig. 3, including 16 pathways in which multiple proteins were repressed by TGF-β1, 5 pathways in which TGF-β1 induced some and repressed some other proteins. Intriguingly, multiple proteins of the ribosome pathway were all induced by TGF-β1.

**Figure 4:**
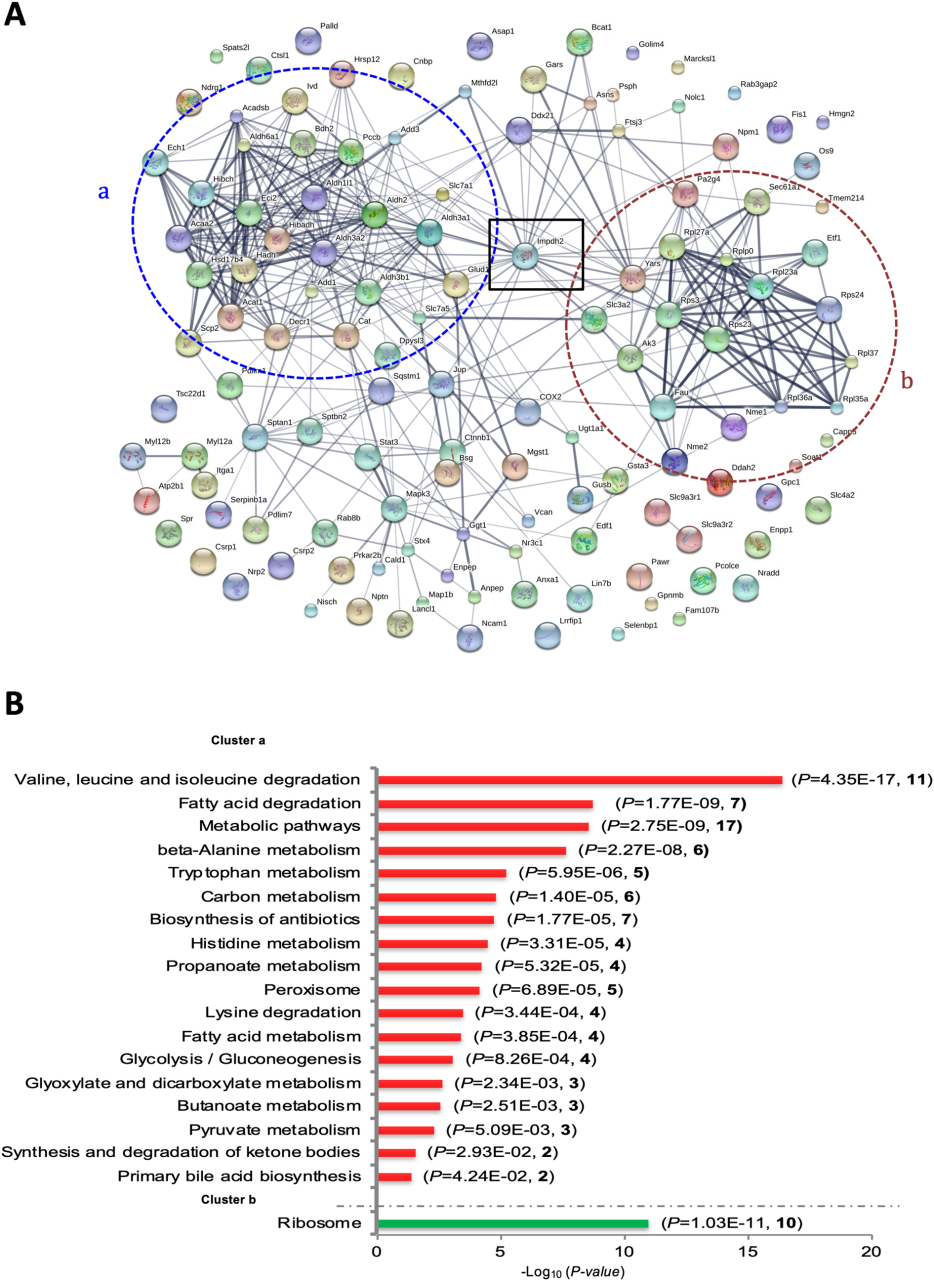
TGF-β1 regulation of the ribosome pathway and many other metabolism pathways. (**A**) STRING analysis reveals predicted direct (physical) or indirect (functional) association among proteins regulated by TGF-β1/control ratios > 1.5 or < 0.67. (**B**) KEGG pathway analyses of proteins in clusters a and b. Cluster a contains proteins involved in multiple metabolic pathways; cluster b is enriched with proteins of the ribosome pathway only. In brackets, *p*-value and number of proteins involved are shown.

STRING analysis (https://string-db.org/) of predicted direct (physical) and indirect (functional) associations among the 628 TGF-β1-regulated proteins created dense interaction networks that do not provide the resolution to see much details (not shown). Focusing on the interaction networks of proteins with TGF-β1/Control ratios > 1.5 or < 0.67, STRING analysis revealed two major clusters of proteins (highlighted as blue circle a and red circle b) connected by inosine-5′-monophosphate dehydrogenase 2 (Impdh2 in black square; Fig. 4A). The accession numbers, TGF-β1/Control ratios, *p*-values and *q*-values of proteins involved in clusters a and b are listed in Supplementary table 3, which showed that most proteins in cluster a, except Dpysl3, Slc7a1 and Slc7a5 (located at periphery of the cluster), were down-regulated by TGF-β1, and that in cluster b, TGF-β1 up-regulated most proteins except Ak3, a mitochondrial enzyme located at the periphery of the cluster.

KEGG pathway analysis of proteins in these two clusters indicated that TGF-β1-induced in vitro model of fibrosis was mainly associated with multiple metabolic pathways (cluster a) and the ribosome pathway (cluster b) as highlighted in Fig. 4B. There were 18 pathways involved in cluster a and the top 3 with the lowest *p-values* were valine, leucine and isoleucine degradation, fatty acid degradation and metabolic pathways. As shown in Supplementary table 4, most proteins involved in metabolic pathways were down-regulated by TGF-β1. However, TGF-β1 significantly induced 10 ribosomal proteins, including four of the small subunit (Rps30, Rps24, Rps23 and Rps3) and six of the large subunit (Rpl36a, Rpl35a, Rpl23a, Rpl37 Rpl27a and Rplp0). Thus, TGF-β1-induced fibrogenesis is associated with a previously unrecognised mismatch of reduced proteins involved in metabolic pathways and increased ribosomal proteins.

**Figure 5:**
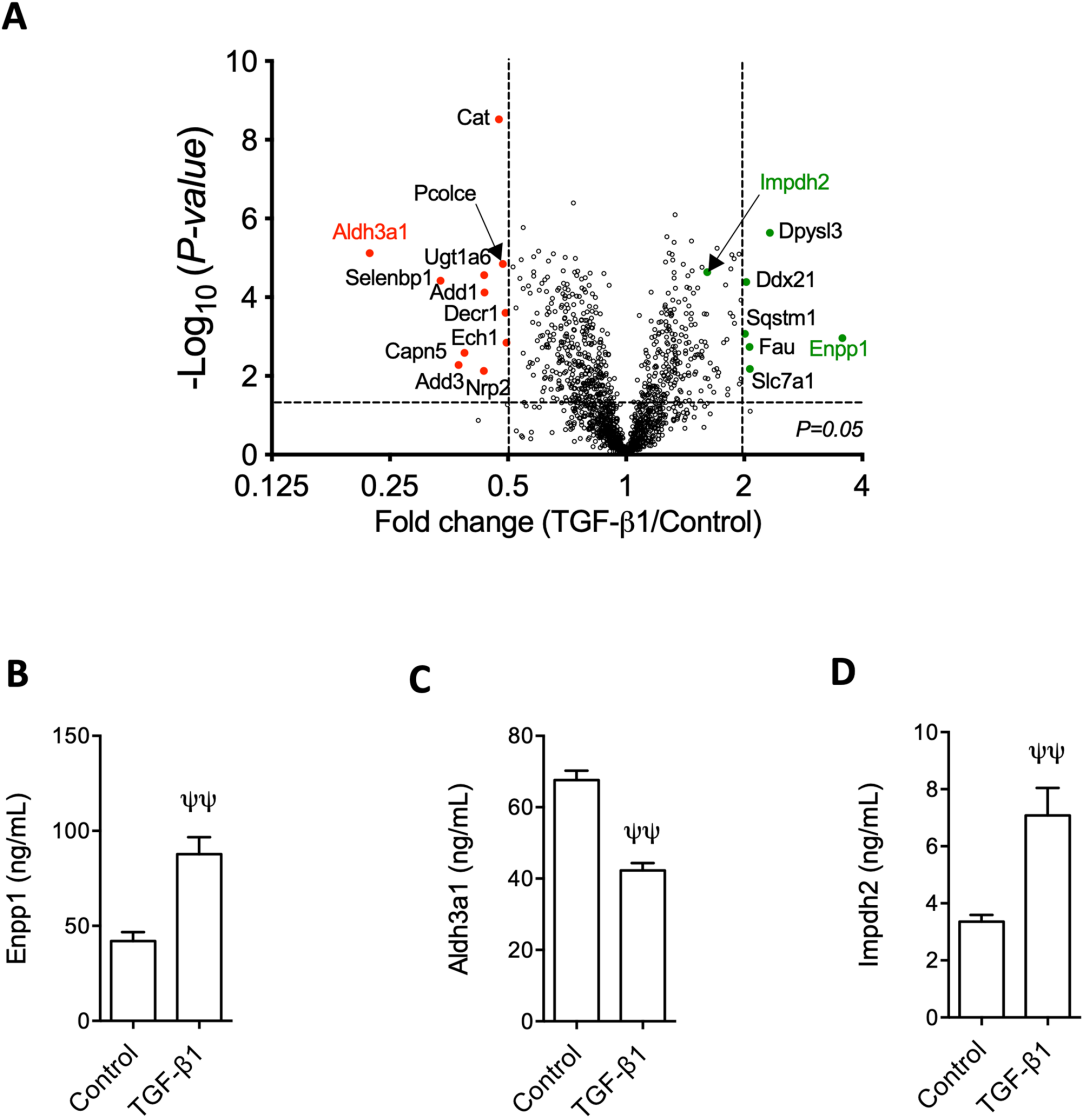
Volcano plot of cell-lysate proteins regulated by TGF-β1 and ELISA validation of TGF-β1 induction of Enpp1, Impdh2 and repression of Aldh3a1. Volcano plot (**A**); ELISA validation of Enpp1 (**B**), Aldh3a1 (**C**) and Impdh2 (**D**). n = 4, ψψ: *p* < 0.01 versus control group. In Fig. 5A, red and green fonts highlight TGF-β1-repressed and -induced proteins subjected to ELISA validation, respectively; red dots highlight proteins repressed by more than twofold; green dots highlight proteins induced by TGF-β1 more than twofold and Impdh2, which was selected for validation by ELISA although it was induced less than twofold.

Volcano plot of all the 1438 identified proteins showed that Enpp1 and Aldh3a1 were the most robustly up- and down-regulated by TGF-β1, respectively (Fig. 5A). They were validated by ELISA (Fig. 5B,C). Also validated by ELISA was TGF-β1-induced Impdh2 (Fig. 5A,D), a middleman between two clusters of TGF-β1-regulated proteins (Fig. 4A).

### Proteomic profiling and ELISA analysis of conditioned media

**Figure 6:**
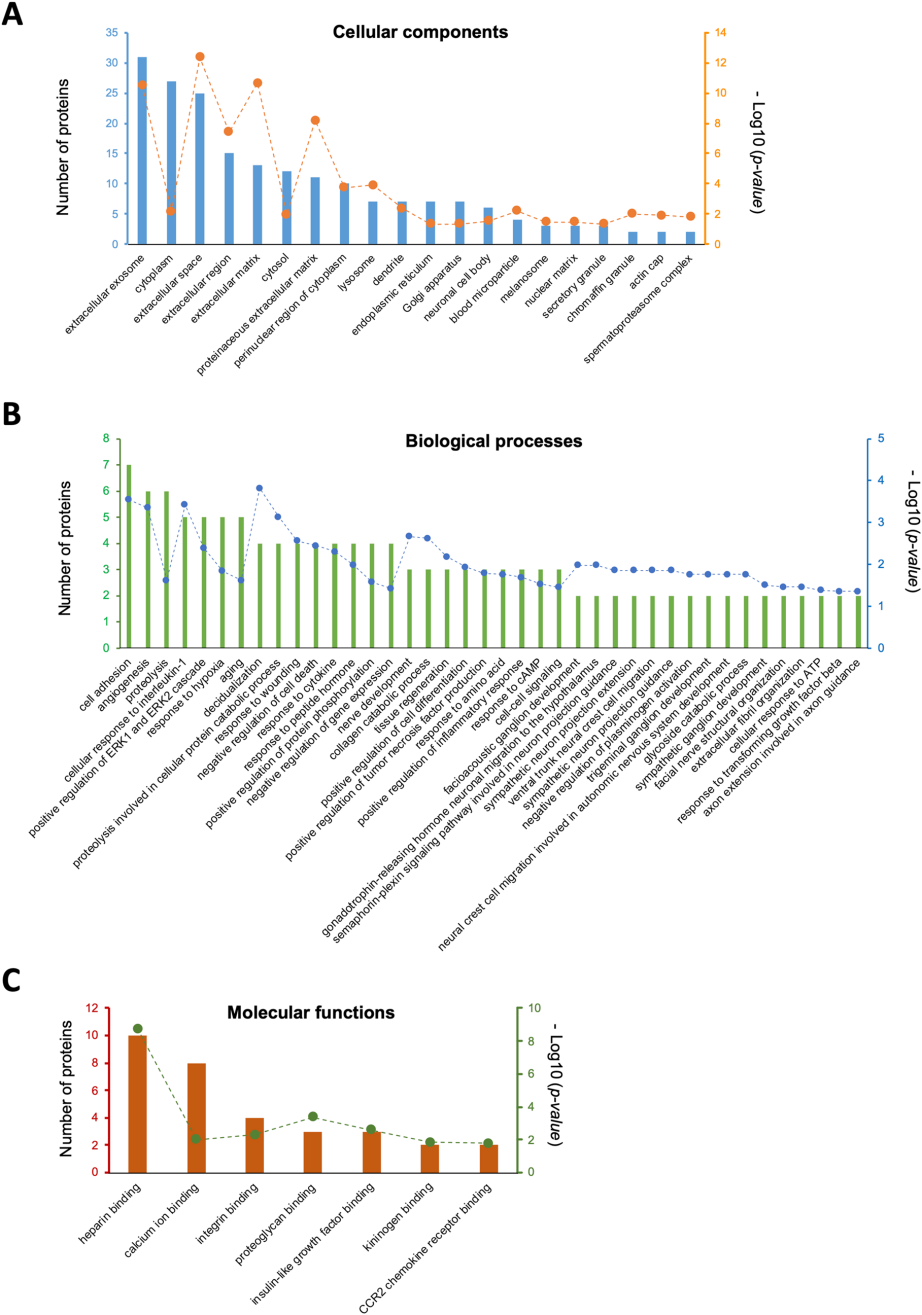
GO analysis of proteins in conditioned media regulated by TGF-β1. Numbers of involved proteins are indicated by the left y-axis and displayed as bars; *p*-values (as -Log10 values) are indicated by the right y-axis and displayed as dots. (**A**) cellular component enrichment; (**B**) biological process enrichment; (**C**) molecular function enrichment.

In total 725 proteins were identified in the conditioned media, of which 62 were regulated by TGF-β1 (*p* < 0.05), including 25 induced and 37 repressed, respectively (Supplementary tables 5)*.* TGF-β1-regulated proteins were enriched in 20 GO cellular component clusters (e.g. extracellular exosomes, cytoplasm, extracellular space, extracellular region, extracellular matrix; Fig. 6A & Supplementary table 6), 40 GO biological process clusters (e.g. cell adhesion, angiogenesis, proteolysis, cellular response to interleukin-1, positive regulation of ERK1 and ERK2 cascade; Fig. 6B & Supplementary table 7) and 7 GO molecular functions associated with binding heparin, calcium ion, integrin, proteoglycan, insulin-like growth factor, kininogen, CCR2 chemokine receptor (Fig. 6C & Supplementary table 8).

**Figure 7:**
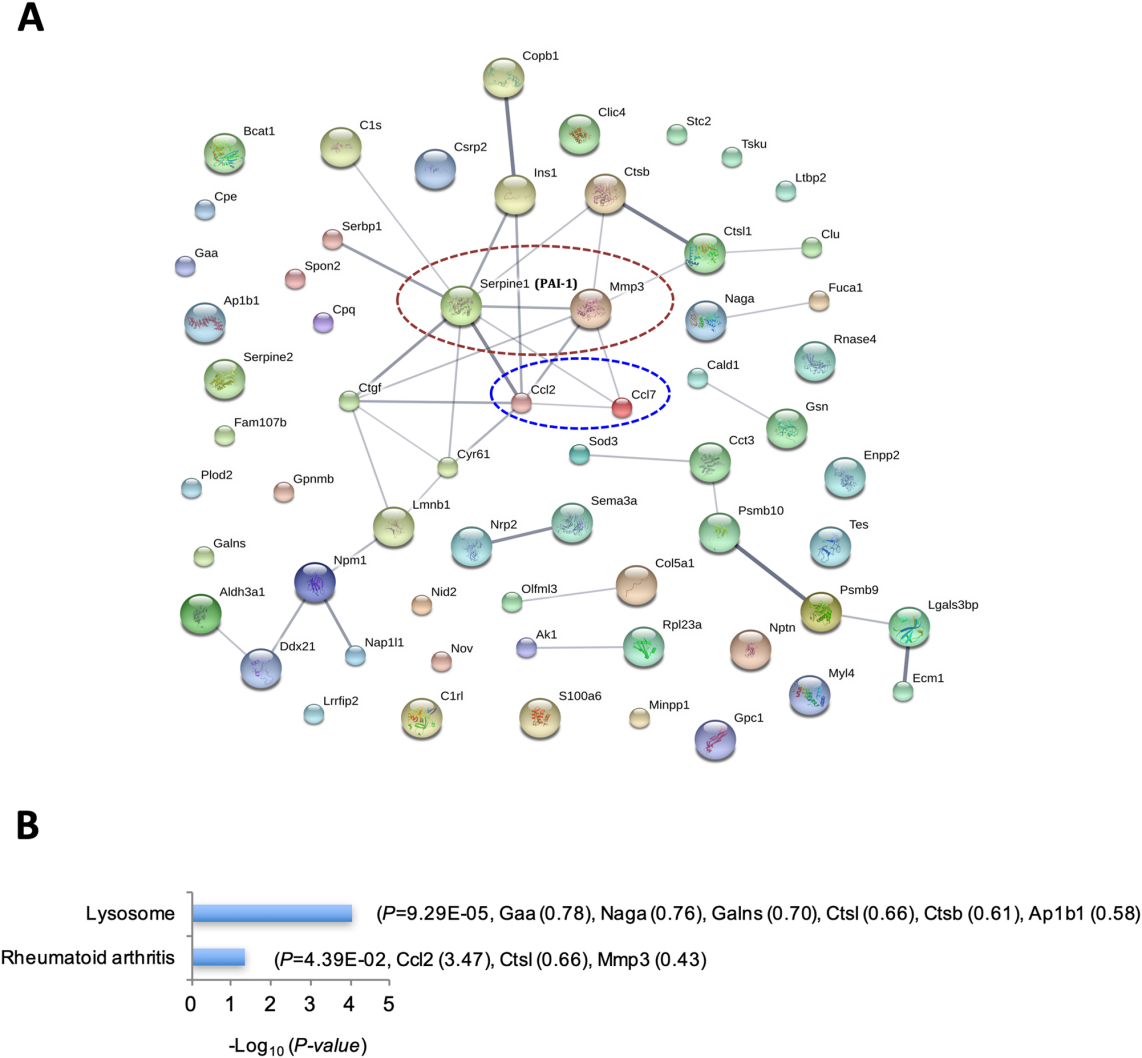
STRING and KEGG pathway analyses of proteins in conditioned media regulated by TGF-β1. (**A**) STRING analysis of TGF-β1-regulated proteins in conditioned media revealed TGF-β1-induced PAI-1 and -repressed Mmp3 (red circle) and TGF-β1-induced chemokines Ccl2 and Ccl7 (blue circle) at the centre of the protein interaction network. (**B**) KEGG pathway enrichment analysis of TGF-β1-regulated proteins in conditioned media revealed only two pathways.

Upon STRING analysis, increased Serpine 1 (PAI-1), a potent inhibitor of collagen degradation, and repressed collagen-degradation enzyme Mmp3 centred the secretomic interaction map of TGF-β1-regulated proteins, suggesting that TGF-β1 repression of collagen degradation might play a crucial role in TGF-β1-induced fibrogenesis. Also located at the centre of this secretomic interaction map were TGF-β1-induced chemokines Ccl2 (MCP-1) and Ccl7 (MCP-3), suggesting that TGF-β1-induced fibrogenesis may be inherently coupled with inflammation by promoting chemotactic effects (Fig. 7A).

KEGG pathway analysis suggested that TGF-β1 regulated the lysosome and the rheumatoid arthritis pathways in the conditioned media (Fig. 7B). Six proteins involved in the lysosome pathway (Gaa, Naga, Galns, Ctsl, Ctsb and Ap1b1) were all reduced in the TGF-β1 group. In the rheumatoid arthritis pathway, TGF-β1 induced Ccl2 and suppressed Ctsl and Mmp3.

**Figure 8:**
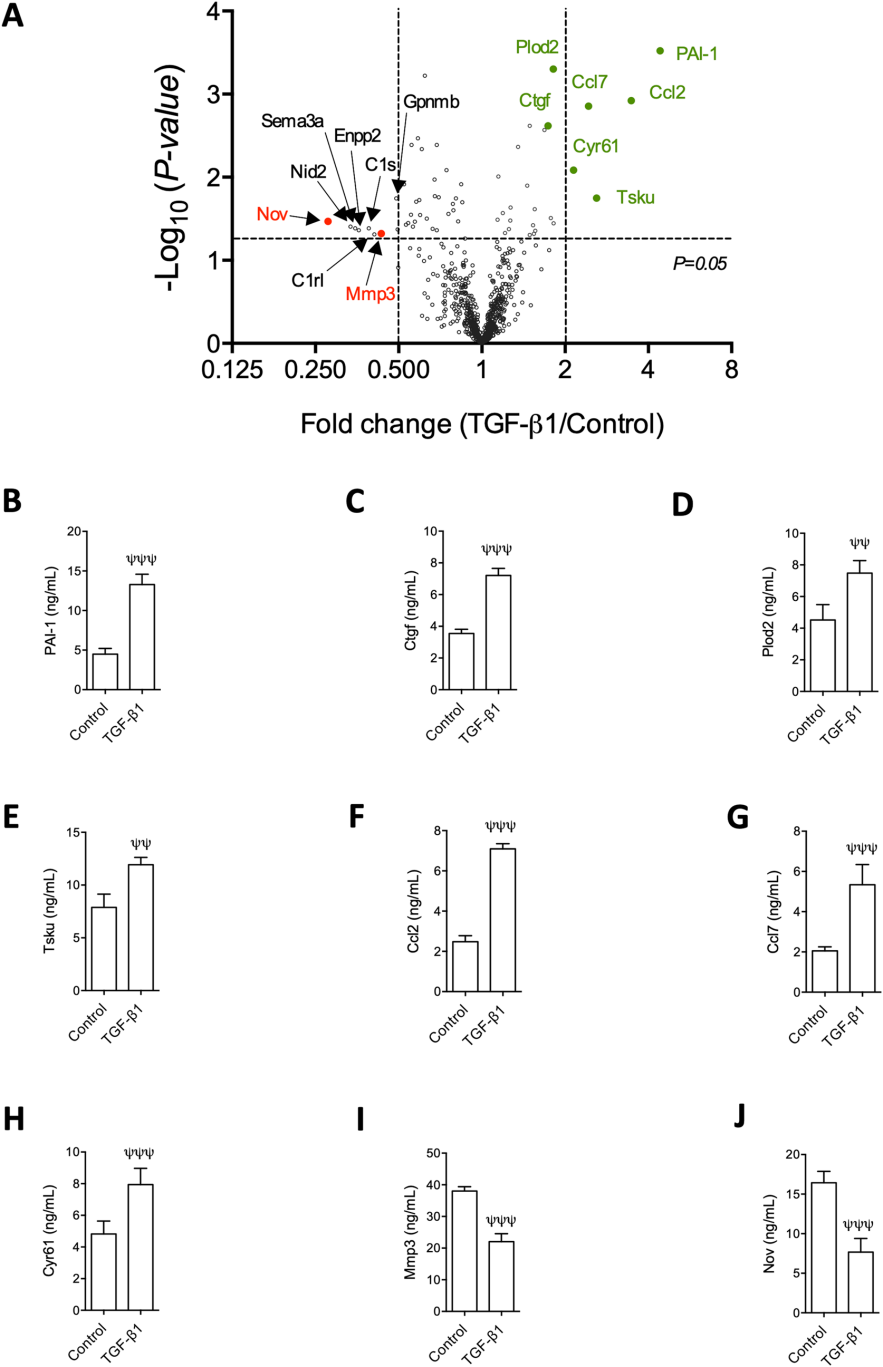
Volcano plot of TGF-β1-regulated secretome and ELISA validation of selected proteins in conditioned media. (**A**) Volcano plot; red and green fonts highlight TGF-β1-repressed and -induced proteins subjected to ELISA validation, respectively; red dots highlight proteins repressed by more than twofold and validated by ELISA; green dots highlight proteins induced by TGF-β1 more than twofold as well as Plod2 and Ctgf, which were selected for ELISA validation despite TGF-β1/Control ratios < 2. (**B**–**J**) ELISA, n = 4, ψψ, ψψψ: *p* < 0.01 and *p* < 0.001 versus control group.

Volcano plot analysis was applied to display the 25 proteins induced by TGF-β1 and the 37 suppressed in conditioned media. As shown in Fig. 8A, PAI-1 was the most significantly and most dramatically induced by TGF-β1, accompanying up-regulation of chemokines Ccl2 and Ccl7, signalling mediators Cyr61 (Ccn1), Ctgf (Ccn2) and Tsku, and collagen crosslinking enzyme Plod2. In addition, TGF-β1 most dramatically repressed Nov (Ccn3) and suppressed collagen degradation enzyme Mmp3, among other proteins. These proteins were validated by ELISA (Fig. 8B–J).

## Discussion

**Figure 9:**
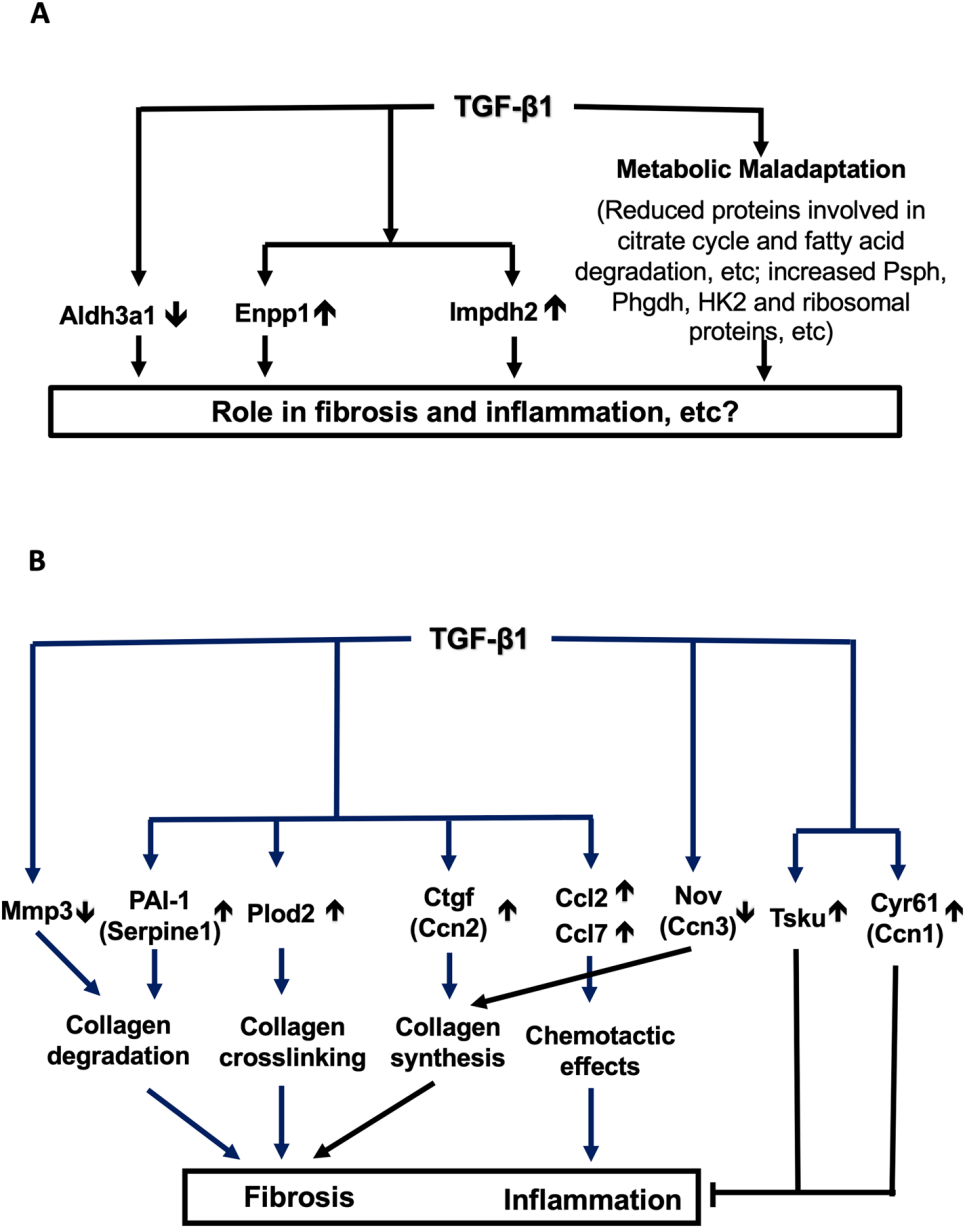
Interpretation of findings of the study. Proteins with upright (induced by TGF-β1) and downwards (repressed by TGF-β1) arrows are validated by ELISA. (**A**) Main findings in cell-lysate proteins, characterised by reduced Aldh3a1, increased Enpp1 and Impdh2 and dysregulated metabolism pathways; (**B**) Main secretomic findings characterised by dysregulation of key matrix degradation regulators (PAI-1↑ and Mmp3 ↓), signalling mediators (Cyr61/Ccn1 ↑, Ctgf/Ccn2 ↑, Nov/Ccn3 ↓ and Tsku ↑) and a collagen crosslinker (Plod2 ↑), and induced chemokines (Ccl2 ↑ and Ccl7 ↑).

The proteomic technology has long been expected to be a powerful tool for unravelling the complex mechanisms of fibrogenesis^25^. In this study, analyses of cell lysates and conditioned media of an in vitro model of TGF-β1-induced fibrosis have led to complementary insights into the biological context of TGF-β1-induced fibrosis, characterised by dysregulated metabolism pathways, reduced Aldh3a1 and increased Enpp1 and Impdh2 in cells (Fig. 9A), and complex extracellular mechanisms regulating fibrogenesis and inflammation (Fig. 9B).

Our results show that TGF-β1-induced fibrogenesis may be an intracellular metabolic disorder characterised by increased intracellular ribosomal proteins and dysregulation of proteins involved in multiple other metabolic pathways (Figs. 3 and 4). To characterise the metabolite changes in conditioned-media and cell-lysate samples, we subjected them to metabonomic studies. Unfortunately, the results were inconclusive, at least in part, due to technical reasons. Nonetheless, our data clearly warrant further metabonomic studies of fibrogenesis and support intracellular metabolic pathways as novel antifibrotic targets^26^.

TGF-β1 is well known to induce cell hypertrophy^27^, which is associated with increased protein synthesis through regulating mTOR^28^. mTORC1 positively regulates ribosomal RNA transcription, the synthesis of ribosomal proteins and other components required for ribosome assembly^29,30^. Given roles for ribosomes in protein synthesis, it may not be surprising that the ribosome pathway is overwhelmingly induced by TGF-β1 (Fig. 3 and Supplementary table 2) and our results warrant further studies on roles for the ribosome pathway as a potential target for novel antifibrotic therapies.

Among proteins enriched in the glycine, serine and threonine metabolism pathway, Psph and Phgdh were induced by TGF-β1 (Supplementary table 2). Selvarajah et al. reported that TGF-β1 induced Psph and Phgdh in primary human lung fibroblasts in a Smad3- and mTORC1/ATF4-dependent manner and that interfering with the mTOR-ATF4 axis reduced Psph- and Phgdh-mediated serine and glycine biosynthesis from glucose, and thus prevented the incorporation of glucose-derived glycine into collagen in TGF-β1 stimulated fibroblasts^31^. TGF-β1-induced Psph and Phgdh were also enriched in carbon metabolism pathway, alongside TGF-β1-induced HK2 (Supplementary table 2). HK2 was the sole TGF-β1-induced protein enriched in glycolysis, gluconeogenesis, galactose, fructose and mannose metabolism pathways, among many other proteins repressed by TGF-β1 (Supplementary table 2). HK2 is known to activate the Hippo pathway and mediate TGF-β1-induced fibrogenesis in murine and human lung fibroblasts and TGF-β-stimulated fibrogenesis can be repressed by pharmacological inhibition of HK2 in a mouse model of bleomycin-induced lung fibrosis^32^. Among TGF-β-regulated proteins enriched in the starch and sucrose metabolism pathway, there were only two induced by TGF-β1, i.e. Enpp1 and HK2. TGF-β1 more robustly induced Enpp1 than HK2 and, indeed, Enpp1 was the most robustly induced cell-lysate protein (Supplementary table 2; Fig. 5A,B). Enpp1 expression in vascular smooth muscle cells and end-plate chondrocytes was TGF-β/Smad3-dependent^33–35^, although its exact role in TGF-β1-induced fibrogenesis remains obscure. It is plausible to hypothesise that, similar to HK-2, Enpp1, as an enzyme, may also mediate TGF-β1-induced fibrogenesis and thus be a novel antifibrotic target (Fig. 9A).

Another plausible target of TGF-β1-induced fibrosis is Impdh2, which links the ribosome and other metabolism pathways (Figs. 4A, 5A,D) and is a known druggable target selectively repressed by Sappanone A, a plant-derived small molecule^37^. As rate-limiting enzymes mediating guanosine and deoxyguanosine biosynthesis, Impdh1 and Impdh2 are upstream of ribosome biogenesis^36^. Non-selective Impdh1 and Impdh 2 inhibitors have multiple therapeutic values, including repressing fibrosis^38–40^.

In contrast to TGF-β1-induced Psph, Phgdh, HK2 and Enpp1, all proteins involved in the tricarboxylic acid (TCA) cycle and a great majority of proteins involved in glycolysis/gluconeogenesis (except HK2) were reduced in TGF-β1 treated group (Fig. 3, Supplementary table 2). This suggests that TGF-β1-induced fibrogenesis is a consequence of metabolic maladaptation, characterised by switching TCA cycles off to give way to glycine and ribosome pathways. Indeed, Zhao et al. reported an overall decrease in TCA cycle metabolites and enzymes in lungs of patients with idiopathic pulmonary fibrosis^41^. In a rat model of liver fibrosis, metabonomic analysis revealed reduced fumaric acid and increased succinic acid, indicating stagnated TCA cycle^42^. The increased succinic acid may have crucial biological consequences in view that the metabolite has an emerging cytokine-like proinflammatory effect^43^.

We found that multiple proteins involved in fatty acid degradation, metabolism and elongation were reduced in the TGF-β1 treated group (Fig. 3, Supplementary table 2). The role for fatty acid metabolism in TGF-β1-induced fibrogenesis in fibroblasts is elusive, but its pro-fibrotic effects in renal tubular and endothelial cells are now well established^44–46^. Thus, targeting dysregulated fatty acid metabolism in TGF-β1-activated fibroblasts may also be a viable antifibrotic strategy.

Among TGF-β1-repressed intracellular proteins, Aldh3a1 and Cat (catalase) stand out (Fig. 5A,C). Catalase is a well-known scavenger of reactive oxygen species and its deficiency is known to promote renal fibrosis^47,48^; Aldh3a1 is involved in multiple metabolic pathways, e.g. glycolysis/gluconeogenesis, β-alanine, histidine, phenylalanine and tyrosine metabolism (Supplementary tables 2 and 4), and is implicated in ageing and response to hypoxia and cAMP (Supplementary table 6). Aldh3a1 is also protective against oxidative stress through multiple mechanisms^49–51^, although the exact role for Aldh3a1 in TGF-β1-induced fibrogenesis in fibroblasts remains elusive and deserves further investigation (Fig. 9A).

Secretomic analysis suggests that lysosome and rheumatoid arthritis pathways were regulated by TGF-β1 (Fig. 7B). TGF-β1 repression of the lysosome pathway may contribute to fibrogenesis and be a potential antifibrotic target, given that lysosomal enzymes may degrade collagens and the lysosome is a recognised potential therapeutic target in a variety of diseases^52^. In the rheumatoid arthritis pathway, TGF-β1 induced Ccl2 and suppressed Ctsl and Mmp3. This may imply their involvement in regulating chronic inflammation as they do in rheumatoid arthritis.

As shown in Fig. 9B, TGF-β1-induced in vitro model of fibrosis in NRK-49F fibroblasts results from reduced collagen degradation, increased crosslinking and dysregulated pro-fibrotic and pro-inflammatory signals. PAI-1 (Serpine1), the protein most robustly induced by TGF-β1 in the conditioned media, is a potent inhibitor of plasminogen activators, which mediate collagen degradation^53,54^. PAI-1 is known to mediate kidney fibrosis^55,56^ and has been considered a potential target for antifibrotic therapy^57^.

The matrix metalloproteinase (MMP) system also play a critical role in collagen degradation^58^. We found that TGF-β1 reduced Mmp3 (Stromelysin), which is known to degrade collagen types II, III, IV, IX, and X, proteoglycans, fibronectin, laminin and elastin^59–61^ and it also activates other MMPs such as MMP-1, MMP-7, and MMP-9, rendering MMP-3 crucial in degradation of collagen and other extracellular matrix proteins^62^. We hypothesise that reduced Mmp3 in the conditioned media, together with increased PAI-1, would contribute to reduced collagen degradation and fibrogenesis. However, roles for Mmp3 might well be more complicated in view that Mmp-3 also has MMP activity-independent functions. For example, a novel transcription factor-like function of human MMP3 in regulating the pro-fibrotic Ctgf (CCN2) gene has been reported^63^.

Collagen crosslinking plays an important role in fibrosis^64^. Plod2 is responsible for the hydroxylation of lysine residues in collagen telopeptides and is essential for collagen pyridinoline cross-link formation^65,66^ and irreversible accumulation of collagen^64,67^. The exact role for TGF-β1-induced Plod2 in fibrosis remains elusive. We hypothesise that Plod2 is profibrotic and that targeting Plod2 may be a novel anti-fibrotic strategy.

Ctgf (Ccn2) is regarded as major profibrotic molecule downstream TGF-β1 and a therapeutic target in renal fibrosis^68–70^. Two additional CCN family members, Cyr61 (Ccn1) and Nov (Ccn3) may also play important roles in fibrogenesis, although these roles are less well established. Kim et al. reported that Cyr61 accumulated in livers of patients with cirrhosis and in murine models of hepatic injury. It triggered cellular senescence in activated hepatic stellate cells and portal fibroblasts, thereby limiting fibrogenesis and promoting regression of experimental liver fibrosis, and indeed, pharmaceutical administration of Cyr61 accelerated regression of established fibrosis^71^. These findings suggest that activation of the Cyr61-induced senescence pathway may hold therapeutic promise. Furthermore, Nov was reported to be a negative regulator of Ctgf both in vitro^72^ and in vivo^73^, with the capacity to limit collagen accumulation and ameliorate fibrosis. Taken together, our results suggest that simultaneously targeting multiple CCN family members may lead to better antifibrotic strategies^74^.

Tsku is a member of the secreted small leucine-rich repeat proteoglycan family that interacts with signalling molecules such as TGF-β1^75^ and was reported to inhibit myofibroblast differentiation by competing with endogenous TGF-β1^76,77^. Thus, TGF-β1-induced Tsku may play an anti-fibrotic role and deserves further investigation.

TGF-β1-induced chemokines Ccl2 (MCP-1) and Ccl7 (MCP-3) may lead to chemotactic effects and recruitment of inflammatory cells, which can in turn regulate fibrogenesis and disease progression. For example, diabetic kidneys of Ccl2-deficient mice or those treated with Ccl2 inhibitors had reduced macrophage infiltration, reduced myofibroblast accumulation and ameliorated renal fibrosis^78,79^, suggesting that Ccl2-mediated macrophage accumulation contributed to myofibroblast activation and renal fibrosis. Roles for Ccl7 in interstitial nephritis have been investigated in a mouse model of obstructive nephropathy. In Ccl7-deficient mice, an early (0–3 days) decrease of inflammatory infiltration was followed by a later (3–8 days) decrease in collagen accumulation. 10–14 days after obstruction, Ccl7-deficient mice displayed increased tubulointerstitial fibrosis and reduced inflammation^80^. Thus, targeting chemokines Ccl2 and Ccl7 may be effective in reducing inflammation, but effects on fibrosis may be more complicated.

Despite all the above interesting results, caution must be exercised in interpreting the findings of this study. First, proteomic methodology used in this study has its limitations. For example, collagen contents in cell lysates and conditioned media were both significantly increased in the TGF-β1-treated group (Fig. 1). Bioimaging analysis of immunofluorescence assays of this in vitro model showed significantly increased collagen types I & III^11^. However, neither collagen type I nor III was significantly altered in our proteomic data, likely because of post-translational modification, e.g. hydroxylation, glycosylation and crosslinking, which make the modified peptides unrecognisable by the proteomic method used in this study, rendering quantification of these proteins unreliable. This problem also applies to kinases and Smad2/3, which are known to be regulated by phosphorylation^19–23^ and can only be solved by specific, targeted proteomic analyses^81–85^. Nonetheless, among proteomic data of all collagens, Col5a1 was the only one significantly regulated by TGF-β1 in both cell lysates (TGF-β1/Control ratio 0.82, *p* < 0.05) and conditioned media (TGF-β1/Control ratio 1.56, *p* < 0.05) (Supplementary tables 1, 5). These results await further validation and are potentially important, given that collagen type V was recently reported to reduce scar size by preventing over-expression and over-activation of integrins^86^. Second, ELISA kits were quantitative, sensitive and commercially available, and thus chosen for validating protein expression. ELISA cannot differentiate different proteoforms, functions of which may vary. Third, NRK-49F cells originally derived from a single cell clone from a rat kidney^13^, thus it may only represent a particular subtype of heterogenous resident renal fibroblasts and may have drifted from its original phenotype in vivo. In addition, sex, strain, species differences must exist. Nonetheless, in view of the long-standing, worldwide use of NRK-49F cells, especially in studying fibrogenesis^8–24^, we propose that TGF- β1-induced in vitro model of fibrosis in this cell line and its proteomic profiling can be a useful reference model for comparison in future studies in other systems in vitro and in vivo.

In conclusion, we have established an unbiased holistic view of an in vitro model of TGF-β1-induced fibrogenesis in NRK-49F cells. Integrating intracellular and extracellular mechanisms, this study has laid the foundation for developing innovative therapeutic strategies in a network pharmacology approach^87^. Finally, data presented in this report are part of a set of experiments comprising seven groups (Supplementary Fig. 1)—in addition to the control and TGF-β1 groups reported here, there are five more groups treated by TGF-β1 in the presence of different antifibrotic agents, comparison of which with the TGF-β1 group has allowed for developing a proteomic roadmap to differentiating mechanisms of different antifibrotics. Analysis of the latter is available online^88,89^ and will be reported elsewhere.

## Materials and methods

### Materials

Human platelet TGF-β1 (R&D Systems, Abingdon, UK) was reconstituted in sterile 4 mM hydrochloric acid (HCl; VWR International Ltd, Lutterworth, UK) and 0.1% bovine serum albumin (BSA; PAA Laboratories GmbH, Pasching, Austria) that had been filter-sterilised through polyethersulfone membrane with 0.2 μm pore size (Sigma-Aldrich, Gillingham, UK). A stock solution of 10 ng/μl TGF-β1 was stored at − 80 °C until use. A vial of MS-SAFE protease and phosphatase inhibitors (Sigma-Aldrich) was dissolved into 2 ml 10 × RIPA lysis and extraction buffer (Sigma-Aldrich) and stored at − 20 °C as a stock solution. 1 × RIPA buffer was used to lyse cells and extract proteins. Bicinchoninic acid (BCA) protein assay (Thermo-Fisher Scientific, Paisley, UK) was used to measure protein concentration of cell lysates. Total collagen contents were measured indirectly through the measurement of hydroxyproline using the QuickZyme Total Collagen Assay (QuickZyme, Leiden, The Netherlands). Soluble collagens in conditioned media were colourimetrically detected by the Sircol soluble collagen assay (Biocolor Ltd., County Antrim, UK). Information of ELISA kits used in this study is listed in Supplementary table 9.

### Cell culture and TGF-β1 treatment

The NRK-49F cell line was purchased from the UK distributor of American Type Culture Collection, LGC Standards (Teddington, UK) and was confirmed as rat cells showing typical morphology of fibroblasts, positive for mesenchymal cell marker, negative for epithelial and endothelial markers, and was free of mycoplasma contamination (Supplementary Fig. 2). The cells were maintained as recommended by the suppliers in Dulbecco's Modified Eagle Medium (DMEM, Thermo-Fisher Scientific) supplemented with 5% foetal calf serum (FCS, Sigma-Aldrich), 100 IU/ml penicillin and 100 μg/ml streptomycin (PAA Laboratories) and 2.5 μg/ml amphotericin B (Thermo-Fisher Scientific) at 37 °C and 5% CO_2_. For experiments, NRK-49F cells were grown in 75 mm^2^ flasks, 2.0 × 10^6^ per flask, in DMEM supplemented with 2.5% FCS and 2.5% Nu-serum replacement for 3 days. Media were changed to 1% FCS DMEM supplemented with 1 × insulin-transferrin-selenium (ITS, Sigma-Aldrich) and the cells were cultured for four additional days. Cells were washed with serum free, phenol red-free DMEM (Thermo-Fisher Scientific) and changed to fresh phenol red-free DMEM and incubated at 37 °C for 30 min. This step was repeated once and the media were changed to serum-free, phenol red-free DMEM with and without 5 ng/ml TGF-β1 and the cells were cultured for 2 days to induce fibrogenesis.

### Harvest and preparation of conditioned media and cell lysates

The conditioned media were collected, mixed with MS-SAFE protease and phosphatase inhibitors (Sigma-Aldrich) and centrifuged at 500 *g* for 5 min at 4 °C to remove pellets of detached cells and large debris. Supernatants were collected and then centrifuged at 2500 *g* for 5 min at 4 °C to remove smaller debris. The final supernatants were stored at − 80 °C until use^90^. To extract cell lysates, flasks were placed on ice and washed thrice with cold phosphate buffer solution (PBS). RIPA buffer with protease and phosphatase inhibitors (Sigma-Aldrich), 1.0 ml per flask, was added to cover cell monolayer, scraped and harvested, and centrifuged at full speed for 10 min to remove debris. The supernatants were harvested, protein concentrations determined, and stored at − 80 °C for further analysis as cell-lysate samples.

### QuickZyme total collagen assay

The assay was modified based on the method described by Prockop and Udenfriend^91^ and conducted as per the manufacturer’s instructions.

### Sircol soluble collagen assay

The assay was a dye-binding method designed for the analysis of acid and pepsin-soluble collagens and conducted as per the manufacturer’s instructions.

### Sample preparation for proteomic analysis

Conditioned media were first concentrated with Amicon Ultra-0.5 3kD centrifugal filtration devices (Merck Millipore, Watford, UK). The cell lysates and conditioned medium samples were denatured by adding 6 M urea/2 M thiourea and reduced with 10 mM dithiothreitol for 1 h at 37 °C. 50 mM iodoacetamide was added for alkylation and the samples were briefly spun down and incubated for 1 h in the dark. Protein contents were precipitated by adding pre-chilled (− 20 °C) acetone (8 × volume) overnight at − 20 °C. Samples were centrifuged at 14,000 *g* for 25 min at 4 °C and supernatants subsequently discarded. The pellets were dried in a Speed Vac vacuum centrifuge (Thermo-Fisher Scientific) and re-suspended in 0.1 M triethylammonium bicarbonate (TEAB, pH = 8.2), and followed by digestion with trypsin (enzyme: substrate ratio, 1:50) overnight at 37 °C. The digestion was stopped by acidification of samples with 1% trifluoroacetic acid (TFA) then the samples were loaded onto 96-well MACROSpin C18 plates (Harvard Apparatus, Cambourne, UK) for peptide clean-up. The plate was firstly activated using 200 μl methanol and centrifuged at 1000 *g* for 1 min, followed by washing steps with 200 µl 80% acetonitrile (ACN), 0.1% TFA, and three equilibration steps using 200 µl 1% ACN, 0.1% TFA with centrifugation (1000 *g* for 1 min) after each step. Samples were loaded into the plate and centrifuged at 1500 *g* for 1 min; the flow-through was reloaded onto the plate a second time and centrifugation repeated. The plate was then washed three times with 200 µl 1% ACN, 0.1% TFA, then centrifuged at 1500 *g* for 1 min. Finally, the peptides were eluted with 170 µl 50% ACN, 0.1% TFA and centrifuged at 1500 *g* for 1 min; this step was repeated and the eluates were combined. Eluted peptides were dried in a Speed Vac vacuum centrifuge and resuspended in 0.1 M TEAB^92,93^. Isobaric labelling of digested peptides was performed using 10-plex TMT reagents (Thermo-Fisher Scientific) according to the manufacturer’s instruction. TMT-labelled samples were mixed and peptides were cleaned-up as described above. Finally, the eluates were dried using Speed Vac vacuum centrifuge and resuspended in 2% ACN, 0.05% TFA^94^.

### HPLC–MS/MS analysis

HPLC–MS/MS was carried out on an HPLC (Dionex UltiMate 3000 RSLCnano) hyphenated with an Orbitrap Fusion Lumos Tribrid Mass Spectrometer (Thermo-Fisher Scientific). Samples were injected onto a trap column (Acclaim PepMap100 C18 Trap, 5 mm × 300 μm, 5 μm, 100 Å) at a flow rate of 25 μl/min for 3 min, using mobile phase A: 2% ACN, 0.1% formic acid (FA) in LC–MS grade water. The following nano-flow LC gradient was then used to separate the peptides at 0.3 μl/min: 0–10 min, 2–10% B; 10–200 min, 10–30% B; 200–210 min, 30–40% B; 210–220 min, 99% B; 220–250 min, 2% B, B = 80% ACN, 0.1% FA in water. The EASY-Spray nano column (Acclaim PepMap100 C18, 50 cm × 75 μm, 2 μm, 100 Å) was set at 45 °C and coupled to an EASY-Spray ion source. The MS spectra were collected from an Orbitrap mass analyser with full ion scan mode over the mass-to-change (*m/z*) range 350–1550 m*/z*. MS/MS were performed on the Top Speed method to fit as many MS^2^ scan in 3 s cycle time using collision-induced dissociation (CID) fragmentation and collected in linear ion trap. For each MS^2^ scan, SPS-MS^3^ method was used to acquire the TMT reporter ion signal using higher-energy collisional dissociation (HCD) fragmentation and collected from Orbitrap. Raw files were analysed using Proteome Discoverer 2.1 and Mascot 2.3.01 (Matrix Science, London, UK) with the following parameters: full MS mass tolerance was set at 10 ppm for precursor ions, MS^2^ mass tolerance was set at 0.8 Da for fragment ions; trypsin was used as enzyme and maximum 2 missed cleavage was allowed; TMT-label on N-terminal and lysine, carbamidomethylation of cysteine were set as fixed modifications and oxidation of methionine, proline, lysine as variable modifications. UniProt/Swiss-Prot rat database (2016_02, 7947 protein entries) was used. Proteins with high identification confidence and with at least two independent peptides were used for quantification. The reporter ion signal was used as quantitative value and normalised to total ion signal then scaled to pooled control channel. Final protein tables were exported for subsequent analysis^92,93^.

### ELISA

ELISA of conditioned media for PAI-1, Ctgf, Plod2, Ccl2, Ccl7, Cyr61, Tsku, Nov and Mmp3, and of cell lysates for Enpp1, Aldh3a1 and Impdh2 were conducted according to the manufactures’ instructions. Absorbance was determined using a micro-plate reader. Experiments were in triplicates.

### Bioinformatics

For proteomic analysis, data were presented by fold-changes indicated by TGF-β1:Control ratios, *p*-value and *q*-values. Volcano plot (Prism 8.0, GraphPad, San Diego, CA) was used to illustrate the distribution of quantified proteins according to *p*-value and TGF-β1:Control ratio. Database for Annotation, Visualisation and Integrate Discovery (DAVID, https://david.ncifcrf.gov/) was used to investigate the biological pathways in which the differentially expressed proteins may participate. The STRING database (https://string-db.org) was then used to build protein–protein interaction networks to further explore their functions. For comparisons of collagen contents and ELISA results, t-tests were used. *p* < 0.05 was regarded as statistically significant.

## Supplementary information

Supplementary Information.
