# Supplementary material for: Proteomic landscape of TGF-β1-induced fibrogenesis in renal fibroblasts
Source: Sci Rep. 2020 Nov 4;10:19054. doi: 10.1038/s41598-020-75989-4 (PMC7642370; doi:10.1038/s41598-020-75989-4)
Supplement: Supplementary file 1 — Supplementary Information. [file 41598_2020_75989_MOESM1_ESM.docx]

**Proteomic landscape of TGF-β1-induced fibrogenesis in renal fibroblasts**

Shujun Zhou^1^,  Xiaoke Yin^2^,  Manuel Mayr^2^,  Mazhar Noor^1^, Peter J Hylands^3^,  Qihe Xu^1,*^

^1^ Renal Sciences and Integrative Chinese Medicine Laboratory, Department of Inflammation Biology, School of Immunology & Microbial Sciences, King's College London, London, UK

^2^ School of Cardiovascular Medicine & Sciences & King's BHF Centre of Research Excellence, King's College London, London, UK

^3^ Institute of Pharmaceutical Science, King's College London, London, UK

* Correspondences to [qihe.xu@kcl.ac.uk](mailto:qihe.xu@kcl.ac.uk)

**Index**

**Index**……………………………………………………………………………………………………….….2

**Supplementary figure 1.** Research design, experimentation and roadmap for reporting………….3

**Supplementary figure 2**. Authentication and quality control of the NRK-49F cells. …………….….4

**Supplementary table 1.** List of all cell-lysate proteins significantly regulated by TGF-β 1, as indicated by proteomic analysis….………………………………..………….6

**Supplementary table 2.** Cell-lysate proteins regulated by TGF-β1 are involved in 42 KEGG pathways……………………………………………………………………….21

**Supplementary table 3.** List of cell-lysate proteins in clusters a and b, Figure 4A………………..24

**Supplementary table 4.** KEGG pathways and enriched cell-lysate proteins regulated by TGF-β1 in clusters a and b, Figure 4A…………………………………………………..25

**Supplementary table 5.** List of all proteins in conditioned media significantly regulated by TGF-β1………………………………………………………………………………..26

**Supplementary table** 6. GO cellular component analysis of proteins in conditioned media significantly regulated by TGF-β1……………………………………………27

**Supplementary table 7.** GO biological process analysis of proteins in conditioned media significantly regulated by TGF-β1……………………………………………28

Supplementary table 8. GO molecular function analysis of proteins in conditioned media significantly regulated by TGF-β1……………………………………………30

Supplementary table 9. A list of ELISA kits used in this project……………………………………..30

**Supplementary figure 1. Research design, experimentation and roadmap for reporting**. In four independent experiments, NRK-49F cells were treated by vehicle, TGF-β1 or TGF-β1 and different concentrations of antifibrotics, followed by proteomic and ELISA analyses. To facilitate focused, clear reporting and in-depth discussions, comparison between Control and Model (TGF-β1) groups is reported here (highlighted by blue fonts); comparisons between the Model (TGF-β1) group and anti-fibrotic treatment groups will be published separately. IN1130, a TGF-β type-1 receptor inhibitor; SRM, methanolic extract of Scutellariae Radix; baicalein, a flavonoid rich in SRM. Media of all groups contained equal volumes (0.1%) of vehicles for baicalein and SRM (dimethyl sulfoxide) and IN1130 (normal saline).

**Supplementary figure 2. Authentication and quality control of the NRK-49F cells. A.** Light microscopy of the cells in culture; **B.** Immunocytofluoresence assays of the cells showing positive staining for vimentin and negative for cytokeratin and factor viii. HKC-8 cells, a human proximal tubular cell line, stained positive for cytokeratin and negative for vimentin and factor viii. Human umbilical vein endothelial cells (HUVEC) stained positive for factor viii and vimentin, negative for cytokeratin; **C.** Polymerase chain reaction (PCR) amplification with species-specific human, mouse and rat primer pairs of the *Cox1* gene. Amplified fragments were visualised by ethidium bromide staining after 2% agarose gel electrophoresis. L: DNA ladder; lanes 1-3: three human cell lines; lanes 4-5: two mouse cell lines; lane 6: NRK-49F (rat); lane 7: another rat cell culture; lanes 8-9: two mouse cell cultures; lane 10: Human control DNA; **D.** Mycoplasma contamination detection. Conditioned media of NRK-49F and another cell culture were harvested for PCR with a LookOut Mycoplasma PCR Detection Kit (MP 0035, Sigma-Aldrich). 20 μl amplification products were run in 1.2% agarose gel with ethidium bromide and photographed under ultraviolet. The negative and positive control samples showed distinct 481 bp and 260 bp bands, respectively. Both cultures were confirmed free of mycoplasma contamination. L: DNA ladder; P: Positive control; S1: NRK-49F cells; S2: another cell line; N: Negative control.

**Supplementary table 1. List of all cell-lysate proteins significantly regulated by TGF-β1, as indicated by proteomic analysis.** Proteins are listed in order of TGF-β1/control ratios; **“•**”: induced by TGF-β1; **“•**”: repressed by TGF-β1**.**

| **Accession** | **Gene Name** | **Protein Name** | **TGF-β1/ control** | **p-value** | **q-value** |
| --- | --- | --- | --- | --- | --- |
| Q924C3 | - Enpp1 | Ectonucleotide pyrophosphatase/phosphodiesterase family member 1 | 3.56 | 0.001098 | 0.007705 |
| Q62952 | - Dpysl3 | Dihydropyrimidinase-related protein 3 | 2.33 | 0.000002 | 0.000671 |
| P62864 | - Fau | 40S ribosomal protein S30 | 2.07 | 0.006588 | 0.026026 |
| P30823 | - Slc7a1 | High affinity cationic amino acid transporter 1 | 2.06 | 0.001813 | 0.010554 |
| Q3B8Q1 | - Ddx21 | Nucleolar RNA helicase 2 | 2.03 | 0.000041 | 0.001172 |
| O08623 | - Sqstm1 | Sequestosome-1 | 2.01 | 0.000856 | 0.006654 |
| Q5U1Z0 | - Rab3gap2 | Rab3 GTPase-activating protein non-catalytic subunit | 1.96 | 0.01178 | 0.039031 |
| Q62736 | - Cald1 | Non-muscle caldesmon | 1.96 | 0.000117 | 0.001918 |
| P83883 | - Rpl36a | 60S ribosomal protein L36a | 1.95 | 0.015902 | 0.04898 |
| P54690 | - Bcat1 | Branched-chain-amino-acid aminotransferase, cytosolic | 1.94 | 0.000008 | 0.000743 |
| P62268 | - Rps23 | 40S ribosomal protein S23 | 1.89 | 0.000462 | 0.004426 |
| Q62908 | - Csrp2 | Cysteine and glycine-rich protein 2 | 1.89 | 0.00001 | 0.000743 |
| P15205 | - Map1b | Microtubule-associated protein 1B | 1.87 | 0.00031 | 0.003401 |
| P52944 | - Pdlim1 | PDZ and LIM domain protein 1 | 1.86 | 0.000008 | 0.000743 |
| P49088 | - Asns | Asparagine synthetase [glutamine-hydrolysing] | 1.85 | 0.000019 | 0.000934 |
| Q9Z1Z9 | - Pdlim7 | PDZ and LIM domain protein 7 | 1.84 | 0.000252 | 0.003069 |
| P18666 | - Myl12b | Myosin regulatory light chain 12B | 1.82 | 0.021371 | 0.060139 |
| Q7TP54 | - Ripor2 | Protein FAM65B | 1.82 | 0.029861 | 0.078216 |
| P69736 | - Edf1 | Endothelial differentiation-related factor 1 | 1.81 | 0.001183 | 0.008184 |
| P41777 | - Nolc1 | Nucleolar and coiled-body phosphoprotein 1 | 1.79 | 0.000048 | 0.001276 |
| P0C5E3 | - Palld | Palladin (Fragment) | 1.77 | 0.001438 | 0.009069 |
| P47875 | - Csrp1 | Cysteine and glycine-rich protein 1 | 1.72 | 0.0082442 | 0.030713 |
| P04646 | - Rpl35a | 60S ribosomal protein L35a | 1.71 | 0.0100208 | 0.034807 |
| Q4KM49 | - Yars | Tyrosine-tRNA ligase, cytoplasmic | 1.71 | 0.0000057 | 0.000743 |
| Q5U2Q7 | - Etf1 | Eukaryotic peptide chain release factor subunit 1 | 1.7 | 0.0064419 | 0.025603 |
| A1L1L2 | - Tmem214 | Transmembrane protein 214 | 1.68 | 0.0070073 | 0.027308 |
| Q5M819 | - Psph | Phosphoserine phosphatase | 1.67 | 0.0258053 | 0.07028 |
| Q5RJT2 | - Ftsj3 | pre-rRNA processing protein FTSJ3 | 1.64 | 0.0046964 | 0.020527 |
| Q05982 | - Nme1 | Nucleoside diphosphate kinase A | 1.64 | 0.0184238 | 0.054401 |
| P62501 | - Tsc22d1 | TSC22 domain family protein 1 | 1.62 | 0.011702 | 0.038862 |
| P61621 | - Sec61a1 | Protein transport protein Sec61 subunit alpha isoform 1 | 1.61 | 0.0497411 | 0.113898 |
| E9PU28 | - Impdh2 | Inosine-5'-monophosphate dehydrogenase 2 | 1.61 | 0.000023 | 0.000934 |
| P62850 | - Rps24 | 40S ribosomal protein S24 | 1.6 | 0.0002702 | 0.003092 |
| P62634 | - Cnbp | Cellular nucleic acid-binding protein | 1.6 | 0.0017603 | 0.01046 |
| O70536 | - Soat1 | Sterol O-acyltransferase 1 | 1.59 | 0.011437 | 0.038607 |
| P13084 | - Npm1 | Nucleophosmin | 1.59 | 0.0013604 | 0.008933 |
| P07314 | - Ggt1 | Gamma-glutamyltranspeptidase 1 | 1.58 | 0.0002644 | 0.003088 |
| P61928 | - Rpl37 | 60S ribosomal protein L37 | 1.56 | 0.0034004 | 0.016354 |
| Q5I0G4 | - Gars | Glycine-tRNA ligase (Fragment) | 1.56 | 0.0000174 | 0.000895 |
| D3ZUA0 | - Mthfd2l | Probable bifunctional methylenetetrahydrofolate dehydrogenase/cyclohydrolase 2 | 1.56 | 0.0023511 | 0.012824 |
| P62752 | - Rpl23a | 60S ribosomal protein L23a | 1.56 | 0.0013196 | 0.008826 |
| P23347 | - Slc4a2 | Anion exchange protein 2 | 1.55 | 0.0030767 | 0.0151 |
| P19804 | - Nme2 | Nucleoside diphosphate kinase B | 1.55 | 0.0240352 | 0.066339 |
| P13832 | - Rlc-a | Myosin regulatory light chain RLC-A | 1.55 | 0.0432235 | 0.103592 |
| P18445 | - Rpl27a | 60S ribosomal protein L27a | 1.54 | 0.0029282 | 0.014592 |
| Q6AYD3 | - Pa2g4 | Proliferation-associated protein 2G4 | 1.52 | 0.0000577 | 0.001342 |
| P62909 | - Rps3 | 40S ribosomal protein S3 | 1.52 | 0.0028506 | 0.014433 |
| Q63016 | - Slc7a5 | Large neutral amino acids transporter small subunit 1 | 1.51 | 0.001791 | 0.010534 |
| Q794F9 | - Slc3a2 | 4F2 cell-surface antigen heavy chain | 1.51 | 0.0029327 | 0.014592 |
| Q66HF9 | - Lrrfip1 | Leucine-rich repeat flightless-interacting protein 1 | 1.5 | 0.0079293 | 0.029771 |
| P19945 | - Rplp0 | 60S acidic ribosomal protein P0 | 1.5 | 0.0004868 | 0.004575 |
| P63326 | - Rps10 | 40S ribosomal protein S10 | 1.5 | 0.009058 | 0.032645 |
| P17078 | - Rpl35 | 60S ribosomal protein L35 | 1.5 | 0.003375 | 0.016286 |
| O35821 | - Mybbp1a | Myb-binding protein 1A | 1.48 | 0.0000782 | 0.001502 |
| Q6P799 | - Sars | Serine-tRNA ligase, cytoplasmic | 1.47 | 0.0000214 | 0.000934 |
| Q62920 | - Pdlim5 | PDZ and LIM domain protein 5 | 1.47 | 0.0000037 | 0.000718 |
| P61515 | - Rpl37a-ps1 | Putative 60S ribosomal protein L37a | 1.47 | 0.0002564 | 0.003069 |
| P62282 | - Rps11 | 40S ribosomal protein S11 | 1.47 | 0.0021085 | 0.011752 |
| O70199 | - Ugdh | UDP-glucose 6-dehydrogenase | 1.46 | 0.0005065 | 0.00467 |
| P63029 | - Tpt1 | Translationally-controlled tumour protein | 1.46 | 0.000356 | 0.003792 |
| P13471 | - Rps14 | 40S ribosomal protein S14 | 1.46 | 0.0037187 | 0.017362 |
| P61354 | - Rpl27 | 60S ribosomal protein L27 | 1.45 | 0.000059 | 0.001342 |
| Q9JHW0 | - Psmb7 | Proteasome subunit beta type-7 | 1.45 | 0.0266744 | 0.071966 |
| P81795 | - Eif2s3 | Eukaryotic translation initiation factor 2 subunit 3 | 1.45 | 0.0417285 | 0.100779 |
| P27881 | - Hk2 | Hexokinase-2 | 1.45 | 0.0014123 | 0.009069 |
| Q9Z0W7 | - Clic4 | Chloride intracellular channel protein 4 | 1.45 | 0.0001658 | 0.002459 |
| P24049 | - Rpl17 | 60S ribosomal protein L17 | 1.45 | 0.0098013 | 0.034376 |
| Q3KRD8 | - Eif6 | Eukaryotic translation initiation factor 6 | 1.44 | 0.0083137 | 0.030812 |
| Q5U216 | - Ddx39a | ATP-dependent RNA helicase DDX39A | 1.44 | 0.0055149 | 0.022955 |
| P13383 | - Ncl | Nucleolin | 1.44 | 0.0000286 | 0.00099 |
| O35763 | - Msn | Moesin | 1.43 | 0.0000559 | 0.001342 |
| P62718 | - Rpl18a | 60S ribosomal protein L18a | 1.42 | 0.0002582 | 0.003069 |
| P06762 | - Hmox1 | Heme oxygenase 1 | 1.42 | 0.0014895 | 0.009267 |
| P38983 | - Rpsa | 40S ribosomal protein SA | 1.42 | 0.0009925 | 0.007172 |
| P62912 | - Rpl32 | 60S ribosomal protein L32 | 1.42 | 0.0026946 | 0.014056 |
| Q9Z1P2 | - Actn1 | Alpha-actinin-1 | 1.42 | 0.0006346 | 0.005399 |
| Q1RP77 | - Nop16 | Nucleolar protein 16 | 1.42 | 0.0023185 | 0.012725 |
| Q5U318 | - Pea15 | Astrocytic phosphoprotein PEA-15 | 1.41 | 0.0440684 | 0.105092 |
| P62250 | - Rps16 | 40S ribosomal protein S16 | 1.41 | 0.0001128 | 0.001865 |
| Q6P7B0 | - Wars | Tryptophan-tRNA ligase, cytoplasmic | 1.41 | 0.0095567 | 0.033683 |
| Q5RKI1 | - Eif4a2 | Eukaryotic initiation factor 4A-II | 1.4 | 0.0000144 | 0.000795 |
| P62963 | - Pfn1 | Profilin-1 | 1.39 | 0.0116613 | 0.038817 |
| Q9QXQ0 | - Actn4 | Alpha-actinin-4 | 1.39 | 0.0005752 | 0.005121 |
| Q7TP47 | - Syncrip | Heterogeneous nuclear ribonucleoprotein Q | 1.38 | 0.0009017 | 0.006789 |
| Q63690 | - Bax | Apoptosis regulator BAX | 1.38 | 0.0105709 | 0.036366 |
| P82808 | - Gfpt1 | Glutamine-fructose-6-phosphate aminotransferase [isomerising] 1 | 1.38 | 0.0252299 | 0.06901 |
| P63245 | - Rack1 | Guanine nucleotide-binding protein subunit beta-2-like 1 | 1.37 | 0.0045998 | 0.020226 |
| Q05175 | - Basp1 | Brain acid soluble protein 1 | 1.37 | 0.0230789 | 0.063945 |
| P47198 | - Rpl22 | 60S ribosomal protein L22 | 1.37 | 0.0013333 | 0.008876 |
| Q63560 | - Map6 | Microtubule-associated protein 6 | 1.37 | 0.0497353 | 0.113898 |
| P62278 | - Rps13 | 40S ribosomal protein S13 | 1.36 | 0.0001329 | 0.002103 |
| A0JPM9 | - Eif3j | Eukaryotic translation initiation factor 3 subunit J | 1.36 | 0.0025824 | 0.013833 |
| Q9ES21 | - Sacm1l | Phosphatidylinositide phosphatase SAC1 | 1.36 | 0.0088705 | 0.032293 |
| Q5U2X6 | - Ccdc47 | Coiled-coil domain-containing protein 47 | 1.35 | 0.0395551 | 0.0969 |
| P12001 | - Rpl18 | 60S ribosomal protein L18 | 1.35 | 0.0041345 | 0.018934 |
| Q712U5 | - Arpp19 | cAMP-regulated phosphoprotein 19 | 1.35 | 0.0048518 | 0.021015 |
| P11762 | - Lgals1 | Galectin-1 | 1.35 | 0.0372277 | 0.091982 |
| Q63525 | - Nudc | Nuclear migration protein nudC | 1.34 | 0.0124122 | 0.040751 |
| Q641X8 | - Eif3e | Eukaryotic translation initiation factor 3 subunit E | 1.34 | 0.0035566 | 0.016879 |
| P62961 | - Ybx1 | Nuclease-sensitive element-binding protein 1 | 1.34 | 0.002966 | 0.014707 |
| Q68FR6 | - Eef1g | Elongation factor 1-gamma | 1.34 | 0.0000589 | 0.001342 |
| Q8K1Q0 | - Nmt1 | Glycylpeptide N-tetradecanoyltransferase 1 | 1.34 | 0.0004197 | 0.004158 |
| P62275 | - Rps29 | 40S ribosomal protein S29 | 1.34 | 0.0475435 | 0.11027 |
| P12749 | - Rpl26 | 60S ribosomal protein L26 | 1.34 | 0.0017594 | 0.01046 |
| O35987 | - Nsfl1c | NSFL1 cofactor p47 | 1.33 | 0.0026978 | 0.014056 |
| P62755 | - Rps6 | 40S ribosomal protein S6 | 1.33 | 0.0008892 | 0.00673 |
| P21533 | - Rpl6 | 60S ribosomal protein L6 | 1.33 | 0.0000008 | 0.000391 |
| P62271 | - Rps18 | 40S ribosomal protein S18 | 1.33 | 0.0036672 | 0.017177 |
| Q63692 | - Cdc37 | Hsp90 co-chaperone Cdc37 | 1.33 | 0.0083535 | 0.030832 |
| Q9R0T3 | - Dnajc3 | DnaJ homolog subfamily C member 3 | 1.33 | 0.0051897 | 0.022079 |
| Q9QVC8 | - Fkbp4 | Peptidyl-prolyl cis-trans isomerase FKBP4 | 1.33 | 0.002405 | 0.013002 |
| D4A4T9 | - Chordc1 | Cysteine and histidine-rich domain-containing protein 1 | 1.33 | 0.0147319 | 0.046356 |
| Q6IRE4 | - Tsg101 | Tumor susceptibility gene 101 protein | 1.33 | 0.0066914 | 0.02629 |
| P39032 | - Rpl36 | 60S ribosomal protein L36 | 1.33 | 0.0071969 | 0.02782 |
| Q9R063 | - Prdx5 | Peroxiredoxin-5, mitochondrial | 1.33 | 0.0002008 | 0.00275 |
| P62630 | - Eef1a1 | Elongation factor 1-alpha 1 | 1.33 | 0.0000234 | 0.000934 |
| P27952 | - Rps2 | 40S ribosomal protein S2 | 1.33 | 0.0000325 | 0.001032 |
| P35427 | - Rpl13a | 60S ribosomal protein L13a | 1.33 | 0.000004 | 0.000718 |
| P69682 | - Necap1 | Adaptin ear-binding coat-associated protein 1 | 1.33 | 0.0173329 | 0.052228 |
| P61480 | - Wdr12 | Ribosome biogenesis protein WDR12 | 1.32 | 0.0012276 | 0.008389 |
| P62832 | - Rpl23 | 60S ribosomal protein L23 | 1.32 | 0.0038515 | 0.017866 |
| P17074 | - Rps19 | 40S ribosomal protein S19 | 1.32 | 0.0017947 | 0.010534 |
| Q62658 | - Fkbp1a | Peptidyl-prolyl cis-trans isomerase FKBP1A | 1.32 | 0.0011745 | 0.008184 |
| P62243 | - Rps8 | 40S ribosomal protein S8 | 1.32 | 0.0000114 | 0.000743 |
| P05197 | - Eef2 | Elongation factor 2 | 1.32 | 0.0001188 | 0.001919 |
| G3V9R8 | - Hnrnpc | Heterogeneous nuclear ribonucleoprotein C | 1.31 | 0.0077468 | 0.029315 |
| O35824 | - Dnaja2 | DnaJ homolog subfamily A member 2 | 1.31 | 0.0008499 | 0.006642 |
| P62845 | - Rps15 | 40S ribosomal protein S15 | 1.31 | 0.0043307 | 0.019502 |
| Q01986 | - Map2k1 | Dual specificity mitogen-activated protein kinase kinase 1 | 1.31 | 0.0075095 | 0.028568 |
| P09895 | - Rpl5 | 60S ribosomal protein L5 | 1.31 | 0.0000082 | 0.000743 |
| P63174 | - Rpl38 | 60S ribosomal protein L38 | 1.31 | 0.0020781 | 0.011627 |
| Q68FR9 | - Eef1d | Elongation factor 1-delta | 1.31 | 0.0002742 | 0.003092 |
| P41123 | - Rpl13 | 60S ribosomal protein L13 | 1.31 | 0.0200022 | 0.05775 |
| Q6PDV7 | - Rpl10 | 60S ribosomal protein L10 | 1.3 | 0.0002967 | 0.003282 |
| P05426 | - Rpl7 | 60S ribosomal protein L7 | 1.3 | 0.0002517 | 0.003069 |
| P02401 | - Rplp2 | 60S acidic ribosomal protein P2 | 1.3 | 0.0159066 | 0.04898 |
| Q9QZ86 | - Nop58 | Nucleolar protein 58 | 1.3 | 0.0002125 | 0.002856 |
| P25886 | - Rpl29 | 60S ribosomal protein L29 | 1.3 | 0.0104309 | 0.03597 |
| P61314 | - Rpl15 | 60S ribosomal protein L15 | 1.29 | 0.0006893 | 0.005664 |
| O88813 | - Acsl5 | Long-chain-fatty-acid-CoA ligase 5 | 1.29 | 0.0066855 | 0.02629 |
| P23358 | - Rpl12 | 60S ribosomal protein L12 | 1.29 | 0.0000076 | 0.000743 |
| Q6AYZ1 | - Tuba1c | Tubulin alpha-1C chain | 1.29 | 0.0116303 | 0.038804 |
| Q71TY3 | - Rps27 | 40S ribosomal protein S27 | 1.29 | 0.0005498 | 0.004941 |
| Q3T1J1 | - Eif5a | Eukaryotic translation initiation factor 5A-1 | 1.28 | 0.0058972 | 0.023821 |
| P28073 | - Psmb6 | Proteasome subunit beta type-6 | 1.28 | 0.0006328 | 0.005399 |
| P34058 | - Hsp90ab1 | Heat shock protein HSP 90-beta | 1.28 | 0.0091371 | 0.032766 |
| Q6P9U8 | - Eif3h | Eukaryotic translation initiation factor 3 subunit H | 1.28 | 0.0000794 | 0.001502 |
| P62856 | - Rps26 | 40S ribosomal protein S26 | 1.28 | 0.0023543 | 0.012824 |
| P62703 | - Rps4x | 40S ribosomal protein S4, X isoform | 1.28 | 0.0015485 | 0.009539 |
| P22509 | - Fbl | rRNA 2'-O-methyltransferase fibrillarin | 1.28 | 0.0133284 | 0.042403 |
| Q641Y8 | - Ddx1 | ATP-dependent RNA helicase DDX1 | 1.27 | 0.0281427 | 0.075362 |
| P20280 | - Rpl21 | 60S ribosomal protein L21 | 1.27 | 0.0007097 | 0.005799 |
| Q5M7W5 | - Map4 | Microtubule-associated protein 4 | 1.27 | 0.0014243 | 0.009069 |
| Q1JU68 | - Eif3a | Eukaryotic translation initiation factor 3 subunit A | 1.27 | 0.0000029 | 0.000691 |
| P62425 | - Rpl7a | 60S ribosomal protein L7a | 1.27 | 0.0002663 | 0.003088 |
| P21531 | - Rpl3 | 60S ribosomal protein L3 | 1.27 | 0.0010299 | 0.007402 |
| P62907 | - Rpl10a | 60S ribosomal protein L10a | 1.27 | 0.0030049 | 0.014849 |
| P62890 | - Rpl30 | 60S ribosomal protein L30 | 1.27 | 0.0005207 | 0.004769 |
| P62919 | - Rpl8 | 60S ribosomal protein L8 | 1.26 | 0.0048897 | 0.021115 |
| Q6VV72 | - Eif1a | Eukaryotic translation initiation factor 1A | 1.26 | 0.0079177 | 0.029771 |
| P11250 | - Rpl34 | 60S ribosomal protein L34 | 1.26 | 0.0004188 | 0.004158 |
| P63018 | - Hspa8 | Heat shock cognate 71 kDa protein | 1.26 | 0.0002032 | 0.002756 |
| Q5XIP1 | - Pelo | Protein pelota homolog | 1.26 | 0.00366 | 0.017177 |
| P22062 | - Pcmt1 | Protein-L-isoaspartate(D-aspartate) O-methyltransferase | 1.26 | 0.0465209 | 0.108942 |
| O08651 | - Phgdh | D-3-phosphoglycerate dehydrogenase | 1.26 | 0.0123408 | 0.040609 |
| P12785 | - Fasn | Fatty acid synthase | 1.26 | 0.0000126 | 0.000752 |
| P49242 | - Rps3a | 40S ribosomal protein S3a | 1.26 | 0.0001581 | 0.002393 |
| P83732 | - Rpl24 | 60S ribosomal protein L24 | 1.26 | 0.0322061 | 0.082701 |
| P60868 | - Rps20 | 40S ribosomal protein S20 | 1.26 | 0.0001764 | 0.002533 |
| Q6AZ50 | - Atg3 | Ubiquitin-like-conjugating enzyme ATG3 | 1.26 | 0.0368564 | 0.091695 |
| D3ZBE5 | - Nek7 | Serine/threonine-protein kinase Nek7 | 1.25 | 0.0101863 | 0.035213 |
| Q5RJR8 | - Lrrc59 | Leucine-rich repeat-containing protein 59 | 1.25 | 0.0095242 | 0.033683 |
| B1WC88 | - N/A | UPF0729 protein C18orf32 homolog | 1.25 | 0.0309817 | 0.080129 |
| Q64270 | - Eif2b1 | Translation initiation factor eIF-2B subunit alpha | 1.25 | 0.0287751 | 0.076344 |
| P50878 | - Rpl4 | 60S ribosomal protein L4 | 1.25 | 0.005755 | 0.023444 |
| Q9R1T1 | - Banf1 | Barrier-to-autointegration factor | 1.25 | 0.0167957 | 0.05111 |
| P21670 | - Psma4 | Proteasome subunit alpha type-4 | 1.24 | 0.0483779 | 0.111665 |
| P43245 | - Abcb1 | Multidrug resistance protein 1 | 1.24 | 0.0028145 | 0.014433 |
| B5DFC8 | - Eif3c | Eukaryotic translation initiation factor 3 subunit C | 1.24 | 0.0007518 | 0.00604 |
| Q9JMJ4 | - Prpf19 | Pre-mRNA-processing factor 19 | 1.23 | 0.0082776 | 0.030758 |
| P17077 | - Rpl9 | 60S ribosomal protein L9 | 1.23 | 0.000249 | 0.003069 |
| Q5XIM5 | - Cdv3 | Protein CDV3 homolog | 1.23 | 0.0449842 | 0.106167 |
| P38062 | - Metap2 | Methionine aminopeptidase 2 | 1.23 | 0.034074 | 0.08657 |
| P29314 | - Rps9 | 40S ribosomal protein S9 | 1.23 | 0.0004207 | 0.004158 |
| Q9WVB1 | - Rab6a | Ras-related protein Rab-6A | 1.23 | 0.0372107 | 0.091982 |
| P62246 | - Rps15a | 40S ribosomal protein S15a | 1.22 | 0.0133251 | 0.042403 |
| P17702 | - Rpl28 | 60S ribosomal protein L28 | 1.22 | 0.0023717 | 0.01287 |
| Q63356 | - Myo1e | Unconventional myosin-Ie | 1.22 | 0.0236681 | 0.065451 |
| P82995 | - Hsp90aa1 | Heat shock protein HSP 90-alpha | 1.21 | 0.0004333 | 0.00422 |
| F1LYQ8 | - Farp1 | FERM, RhoGEF and pleckstrin domain-containing protein 1 | 1.21 | 0.000808 | 0.006419 |
| Q66HA8 | - Hsph1 | Heat shock protein 105 kDa | 1.21 | 0.0021275 | 0.011812 |
| B0BNA7 | - Eif3i | Eukaryotic translation initiation factor 3 subunit I | 1.21 | 0.0008732 | 0.006679 |
| P07335 | - Ckb | Creatine kinase B-type | 1.21 | 0.0312553 | 0.080691 |
| Q9EQR2 | - Agps | Alkyldihydroxyacetonephosphate synthase, peroxisomal | 1.21 | 0.0257579 | 0.07028 |
| P37397 | - Cnn3 | Calponin-3 | 1.2 | 0.0026069 | 0.013833 |
| P04644 | - Rps17 | 40S ribosomal protein S17 | 1.2 | 0.0185435 | 0.054643 |
| O89049 | - Txnrd1 | Thioredoxin reductase 1, cytoplasmic | 1.2 | 0.0058558 | 0.023787 |
| Q5XHY5 | - Tars | Threonine--tRNA ligase, cytoplasmic | 1.19 | 0.0013528 | 0.008924 |
| Q7TNY6 | - Acbd3 | Golgi resident protein GCP60 | 1.19 | 0.0208809 | 0.059345 |
| Q811A3 | - Plod2 | Procollagen-lysine,2-oxoglutarate 5-dioxygenase 2 | 1.19 | 0.042638 | 0.102531 |
| P60123 | - Ruvbl1 | RuvB-like 1 | 1.19 | 0.0055658 | 0.022955 |
| Q5RJR2 | - Twf1 | Twinfilin-1 | 1.19 | 0.0497102 | 0.113898 |
| P84100 | - Rpl19 | 60S ribosomal protein L19 | 1.19 | 0.0189263 | 0.055205 |
| P68035 | - Actc1 | Actin, alpha cardiac muscle 1 | 1.19 | 0.0085285 | 0.031286 |
| Q2PQA9 | - Kif5b | Kinesin-1 heavy chain | 1.19 | 0.0034128 | 0.016359 |
| P38656 | - Ssb | Lupus La protein homolog | 1.19 | 0.0344342 | 0.087177 |
| P24051 | - Rps27l | 40S ribosomal protein S27-like | 1.18 | 0.0006568 | 0.005523 |
| Q5U312 | - Rai14 | Ankycorbin | 1.18 | 0.0009869 | 0.007168 |
| Q62881 | - Nol3 | Nucleolar protein 3 | 1.18 | 0.0301995 | 0.078958 |
| P40615 | - Dkc1 | H/ACA ribonucleoprotein complex subunit 4 | 1.18 | 0.0466678 | 0.108942 |
| Q6AYK8 | - Eif3d | Eukaryotic translation initiation factor 3 subunit D | 1.18 | 0.006048 | 0.024226 |
| Q63507 | - Rpl14 | 60S ribosomal protein L14 | 1.18 | 0.0178203 | 0.053231 |
| Q9Z270 | - Vapa | Vesicle-associated membrane protein-associated protein A | 1.18 | 0.0101869 | 0.035213 |
| Q9Z1A6 | - Hdlbp | Vigilin | 1.18 | 0.0015655 | 0.009539 |
| Q9JIH7 | - Wnk1 | Serine/threonine-protein kinase WNK1 | 1.17 | 0.02844 | 0.075834 |
| P05765 | - Rps21 | 40S ribosomal protein S21 | 1.17 | 0.0208823 | 0.059345 |
| Q4G061 | - Eif3b | Eukaryotic translation initiation factor 3 subunit B | 1.17 | 0.0178759 | 0.053231 |
| Q63228 | - Gmfb | Glia maturation factor beta | 1.17 | 0.0371693 | 0.091982 |
| Q63716 | - Prdx1 | Peroxiredoxin-1 | 1.16 | 0.001045 | 0.007402 |
| Q66HR2 | - Mapre1 | Microtubule-associated protein RP/EB family member 1 | 1.16 | 0.0000741 | 0.001488 |
| Q68FQ0 | - Cct5 | T-complex protein 1 subunit epsilon | 1.16 | 0.0004009 | 0.004088 |
| P45592 | - Cfl1 | Cofilin-1 | 1.15 | 0.0199921 | 0.05775 |
| Q66HL2 | - Cttn | Src substrate cortactin | 1.15 | 0.0188479 | 0.055088 |
| P28480 | - Tcp1 | T-complex protein 1 subunit alpha | 1.15 | 0.0165744 | 0.050696 |
| P43138 | - Apex1 | DNA- (apurinic or apyrimidinic site) lyase | 1.15 | 0.0129767 | 0.041934 |
| P30427 | - Plec | Plectin | 1.15 | 0.0098421 | 0.034435 |
| Q62812 | - Myh9 | Myosin-9 | 1.15 | 0.0269145 | 0.072478 |
| P52296 | - Kpnb1 | Importin subunit beta-1 | 1.14 | 0.0218246 | 0.060821 |
| P18420 | - Psma1 | Proteasome subunit alpha type-1 | 1.14 | 0.0195399 | 0.056879 |
| Q6AXS5 | - Serbp1 | Plasminogen activator inhibitor 1 RNA-binding protein | 1.14 | 0.0303913 | 0.07919 |
| Q9QZA2 | - Pdcd6ip | Programmed cell death 6-interacting protein | 1.14 | 0.0032095 | 0.015698 |
| Q04462 | - Vars | Valine--tRNA ligase | 1.14 | 0.04176 | 0.100779 |
| P62859 | - Rps28 | 40S ribosomal protein S28 | 1.14 | 0.0445586 | 0.105909 |
| P24155 | - Thop1 | Thimet oligopeptidase | 1.14 | 0.0046095 | 0.020226 |
| Q9Z269 | - Vapb | Vesicle-associated membrane protein-associated protein B | 1.14 | 0.0065536 | 0.025961 |
| P37285 | - Klc1 | Kinesin light chain 1 | 1.14 | 0.0289927 | 0.07678 |
| Q5EGY4 | - Ykt6 | Synaptobrevin homolog YKT6 | 1.13 | 0.0308115 | 0.079976 |
| P49791 | - Nup153 | Nuclear pore complex protein Nup153 | 1.13 | 0.0043654 | 0.019556 |
| Q9EPH8 | - Pabpc1 | Polyadenylate-binding protein 1 | 1.13 | 0.0026369 | 0.013899 |
| Q6P502 | - Cct3 | T-complex protein 1 subunit gamma | 1.12 | 0.0021966 | 0.012149 |
| Q63610 | - Tpm3 | Tropomyosin alpha-3 chain | 1.12 | 0.0050609 | 0.021659 |
| Q5XIG8 | - Strap | Serine-threonine kinase receptor-associated protein | 1.12 | 0.0154925 | 0.048159 |
| Q5HZY0 | - Ubxn4 | UBX domain-containing protein 4 | 1.11 | 0.0188267 | 0.055088 |
| D3ZTX0 | - Tmed7 | Transmembrane emp24 domain-containing protein 7 | 1.11 | 0.033877 | 0.086221 |
| P35281 | - Rab10 | Ras-related protein Rab-10 | 1.11 | 0.044936 | 0.106167 |
| Q08163 | - Cap1 | Adenylyl cyclase-associated protein 1 | 1.11 | 0.0309408 | 0.080129 |
| Q6MG08 | - Abcf1 | ATP-binding cassette sub-family F member 1 | 1.1 | 0.0284181 | 0.075834 |
| P40329 | - Rars | Arginine-tRNA ligase, cytoplasmic | 1.1 | 0.0318503 | 0.081933 |
| Q5XIM9 | - Cct2 | T-complex protein 1 subunit beta | 1.1 | 0.0055078 | 0.022955 |
| Q794E4 | - Hnrnpf | Heterogeneous nuclear ribonucleoprotein F | 1.1 | 0.0265802 | 0.071847 |
| O35814 | - Stip1 | Stress-induced-phosphoprotein 1 | 1.08 | 0.0002923 | 0.003259 |
| Q07266 | - Dbn1 | Drebrin | 1.08 | 0.0466116 | 0.108942 |
| P05712 | - Rab2a | Ras-related protein Rab-2A | 1.07 | 0.0127148 | 0.041366 |
| Q66H80 | - Arcn1 | Coatomer subunit delta | 1.05 | 0.0461805 | 0.108687 |
| Q4KMA2 | - Rad23b | UV excision repair protein RAD23 homolog B | 0.95 | 0.0287152 | 0.076326 |
| Q9Z1X1 | - Esyt1 | Extended synaptotagmin-1 | 0.93 | 0.0115308 | 0.038741 |
| Q64560 | - Tpp2 | Tripeptidyl-peptidase 2 | 0.93 | 0.0356649 | 0.089349 |
| Q6TUG0 | - Dnajb11 | DnaJ homolog subfamily B member 11 | 0.92 | 0.0200398 | 0.05775 |
| Q9WVC0 | - Sep-07 | Septin-7 | 0.92 | 0.0450362 | 0.106167 |
| Q00438 | - Ptbp1 | Polypyrimidine tract-binding protein 1 | 0.91 | 0.0354257 | 0.088904 |
| B0BNG0 | - Emc2 | ER membrane protein complex subunit 2 | 0.91 | 0.0251889 | 0.06901 |
| O08629 | - Trim28 | Transcription intermediary factor 1-beta | 0.91 | 0.0122504 | 0.040404 |
| F1MA98 | - Tpr | Nucleoprotein TPR | 0.91 | 0.0376338 | 0.092826 |
| P31977 | - Ezr | Ezrin | 0.91 | 0.0263836 | 0.071449 |
| P61980 | - Hnrnpk | Heterogeneous nuclear ribonucleoprotein K | 0.91 | 0.0000497 | 0.001276 |
| P15791 | - Camk2d | Calcium/calmodulin-dependent protein kinase type II subunit delta | 0.91 | 0.0443771 | 0.105653 |
| Q9JIL3 | - Ilf3 | Interleukin enhancer-binding factor 3 | 0.9 | 0.0028383 | 0.014433 |
| Q63355 | - Myo1c | Unconventional myosin-Ic | 0.9 | 0.0029042 | 0.014592 |
| P46462 | - Vcp | Transitional endoplasmic reticulum ATPase | 0.9 | 0.0027992 | 0.014427 |
| P28023 | - Dctn1 | Dynactin subunit 1 | 0.9 | 0.0166048 | 0.050696 |
| O54975 | - Xpnpep1 | Xaa-Pro aminopeptidase 1 | 0.9 | 0.000848 | 0.006642 |
| Q91Y78 | - Uchl3 | Ubiquitin carboxyl-terminal hydrolase isozyme L3 | 0.89 | 0.0227242 | 0.063084 |
| P35435 | - Atp5c1 | ATP synthase subunit gamma, mitochondrial | 0.89 | 0.0434718 | 0.104014 |
| Q6IUR5 | - Nenf | Neudesin | 0.89 | 0.0087244 | 0.031842 |
| P16036 | - Slc25a3 | Phosphate carrier protein, mitochondrial | 0.89 | 0.005468 | 0.022924 |
| P63331 | - Ppp2ca | Serine/threonine-protein phosphatase 2A catalytic subunit alpha isoform | 0.89 | 0.0095396 | 0.033683 |
| P29410 | - Ak2 | Adenylate kinase 2, mitochondrial | 0.89 | 0.0384143 | 0.094427 |
| Q91Y81 | - Sep-02 | Septin-2 | 0.89 | 0.0028444 | 0.014433 |
| O88989 | - Mdh1 | Malate dehydrogenase, cytoplasmic | 0.88 | 0.0024969 | 0.013448 |
| Q4V7C6 | - Gmps | GMP synthase [glutamine-hydrolyzing] | 0.88 | 0.0216074 | 0.060568 |
| P00388 | - Por | NADPH-cytochrome P450 reductase | 0.88 | 0.0276875 | 0.074281 |
| O88370 | - Pip4k2c | Phosphatidylinositol 5-phosphate 4-kinase type-2 gamma | 0.88 | 0.000025 | 0.000946 |
| Q63081 | - Pdia6 | Protein disulfide-isomerase A6 | 0.88 | 0.0043397 | 0.16632 |
| Q4FZT0 | - Stoml2 | Stomatin-like protein 2, mitochondrial | 0.88 | 0.0099038 | 0.034567 |
| P06685 | - Atp1a1 | Sodium/potassium-transporting ATPase subunit alpha-1 | 0.88 | 0.0017899 | 0.010534 |
| P37996 | - Arl3 | ADP-ribylation factor-like protein 3 | 0.88 | 0.0291822 | 0.076998 |
| P84092 | - Ap2m1 | AP-2 complex subunit mu | 0.87 | 0.0171178 | 0.051822 |
| Q6AY23 | - Pycr2 | Pyrroline-5-carboxylate reductase 2 | 0.87 | 0.0169387 | 0.051388 |
| Q9JK72 | - Ccs | Copper chaperone for superoxide dismutase | 0.87 | 0.0006857 | 0.005664 |
| P10111 | - Ppia | Peptidyl-prolyl cis-trans isomerase A | 0.87 | 0.010764 | 0.036679 |
| P04897 | - Gnai2 | Guanine nucleotide-binding protein G(i) subunit alpha-2 | 0.87 | 0.0178793 | 0.053231 |
| Q68FW9 | - Cops3 | COP9 signalosome complex subunit 3 | 0.87 | 0.0113669 | 0.038551 |
| O08839 | - Bin1 | Myc box-dependent-interacting protein 1 | 0.87 | 0.009192 | 0.032802 |
| Q5U211 | - Snx3 | Sorting nexin-3 | 0.86 | 0.0052603 | 0.022183 |
| Q5SGE0 | - Lrpprc | Leucine-rich PPR motif-containing protein, mitochondrial | 0.86 | 0.0014064 | 0.009069 |
| P48500 | - Tpi1 | Triosephosphate isomerase | 0.86 | 0.047181 | 0.109784 |
| Q6AY09 | - Hnrnph2 | Heterogeneous nuclear ribonucleoprotein H2 | 0.86 | 0.0011837 | 0.008184 |
| Q66HG4 | - Galm | Aldose 1-epimerase | 0.86 | 0.0260616 | 0.070844 |
| Q5U300 | - Uba1 | Ubiquitin-like modifier-activating enzyme 1 | 0.86 | 0.0082328 | 0.030713 |
| Q66H94 | - Fkbp9 | Peptidyl-prolyl cis-trans isomerase FKBP9 | 0.86 | 0.0036294 | 0.017112 |
| Q3MIE4 | - Vat1 | Synaptic vesicle membrane protein VAT-1 homolog | 0.86 | 0.0293934 | 0.077413 |
| P14408 | - Fh | Fumarate hydratase, mitochondrial | 0.86 | 0.044906 | 0.106167 |
| Q5XIH7 | - Phb2 | Prohibitin-2 | 0.86 | 0.0002638 | 0.003088 |
| Q2LAP6 | - Tes | Testin | 0.86 | 0.0060924 | 0.024336 |
| P04550 | - Ptms | Parathymosin | 0.86 | 0.017361 | 0.052228 |
| P63312 | - Tmsb10 | Thymosin beta-10 | 0.86 | 0.0272058 | 0.073125 |
| P16636 | - Lox | Protein-lysine 6-oxidase | 0.86 | 0.0083618 | 0.030832 |
| Q5XIN6 | - Letm1 | LETM1 and EF-hand domain-containing protein 1, mitochondrial | 0.86 | 0.0107029 | 0.036558 |
| P07323 | - Eno2 | Gamma-enolase | 0.86 | 0.0178061 | 0.053231 |
| P85515 | - Actr1a | Alpha-centractin | 0.86 | 0.0014028 | 0.009069 |
| Q3KR86 | - Immt | MIC complex subunit Mic60 (Fragment) | 0.86 | 0.0284774 | 0.075834 |
| Q5U2U0 | - Clpx | ATP-dependent Clp protease ATP-binding subunit clpX-like, mitochondrial | 0.85 | 0.0090503 | 0.032645 |
| Q4QQW4 | - Hdac1 | Histone deacetylase 1 | 0.85 | 0.0026765 | 0.014047 |
| Q9JLJ3 | - Aldh9a1 | 4-trimethylaminobutyraldehyde dehydrogenase | 0.85 | 0.0019633 | 0.011115 |
| Q505J8 | - Farsa | Phenylalanine-tRNA ligase alpha subunit | 0.85 | 0.0429945 | 0.103215 |
| O35094 | - Timm44 | Mitochondrial import inner membrane translocase subunit TIM44 | 0.85 | 0.0304649 | 0.07922 |
| P25286 | - Atp6v0a1 | V-type proton ATPase 116 kDa subunit a isoform 1 | 0.85 | 0.0359705 | 0.089646 |
| P35704 | - Prdx2 | Peroxiredoxin-2 | 0.85 | 0.0056877 | 0.023368 |
| Q4QQW8 | - Plbd2 | Putative phospholipase B-like 2 | 0.85 | 0.003479 | 0.016621 |
| Q8CGV7 | - Thtpa | Thiamine-triphosphatase | 0.85 | 0.015968 | 0.049064 |
| P43244 | - Matr3 | Matrin-3 | 0.85 | 0.0295578 | 0.077627 |
| Q80X08 | - Washc2 | WASH complex subunit FAM21 | 0.85 | 0.0044541 | 0.01983 |
| Q62991 | - Scfd1 | Sec1 family domain-containing protein 1 | 0.85 | 0.0106231 | 0.036371 |
| Q9JM53 | - Aifm1 | Apoptosis-inducing factor 1, mitochondrial | 0.85 | 0.0071194 | 0.027669 |
| P69060 | - Cmas | N-acylneuraminate cytidylyltransferase | 0.84 | 0.0291077 | 0.076943 |
| Q6PCT3 | - Tpd52l2 | Tumor protein D54 | 0.84 | 0.0422336 | 0.101729 |
| Q68FY0 | - Uqcrc1 | Cytochrome b-c1 complex subunit 1, mitochondrial | 0.84 | 0.0172724 | 0.05218 |
| P27615 | - Scarb2 | Lysosome membrane protein 2 | 0.84 | 0.0128804 | 0.04181 |
| P54001 | - P4ha1 | Prolyl 4-hydroxylase subunit alpha-1 | 0.84 | 0.021183 | 0.059963 |
| P18665 | - Mrpl3 | 39S ribosomal protein L3, mitochondrial | 0.84 | 0.0186807 | 0.054822 |
| Q641X3 | - Hexa | Beta-hex aminidase subunit alpha | 0.84 | 0.0055643 | 0.022955 |
| P04636 | - Mdh2 | Malate dehydrogenase, mitochondrial | 0.84 | 0.0331004 | 0.084394 |
| P63086 | - Mapk1 | Mitogen-activated protein kinase 1 | 0.84 | 0.0002483 | 0.003069 |
| P97629 | - Lnpep | Leucyl-cystinyl aminopeptidase | 0.84 | 0.0001766 | 0.002533 |
| Q5U2Z3 | - Nap1l4 | Nucleosome assembly protein 1-like 4 | 0.84 | 0.0067304 | 0.0263 |
| O35244 | - Prdx6 | Peroxiredoxin-6 | 0.83 | 0.0001017 | 0.001741 |
| Q62636 | - Rap1b | Ras-related protein Rap-1b | 0.83 | 0.0115236 | 0.038741 |
| P13668 | - Stmn1 | Stathmin | 0.83 | 0.0095801 | 0.033683 |
| Q5I0P2 | - Gcsh | Glycine cleavage system H protein, mitochondrial | 0.83 | 0.0213464 | 0.060139 |
| Q4KLH4 | - Pspc1 | Paraspeckle component 1 | 0.83 | 0.0014221 | 0.009069 |
| Q63269 | - Itpr3 | Inositol 1,4,5-trisphosphate receptor type 3 | 0.83 | 0.049323 | 0.113482 |
| Q62826 | - Hnrnpm | Heterogeneous nuclear ribonucleoprotein M | 0.83 | 0.0041922 | 0.019017 |
| Q9Z2L0 | - Vdac1 | Voltage-dependent anion-selective channel protein 1 | 0.82 | 0.013179 | 0.042376 |
| Q4KM73 | - Cmpk1 | UMP-CMP kinase | 0.82 | 0.0126134 | 0.041154 |
| Q4KLN7 | - Arfgap3 | ADP-ribylation factor GTPase-activating protein 3 | 0.82 | 0.0004936 | 0.004609 |
| B0BNM1 | - Naxe | NAD(P)H-hydrate epimerase | 0.82 | 0.0151538 | 0.047579 |
| Q66HC5 | - Nup93 | Nuclear pore complex protein Nup93 | 0.82 | 0.001045 | 0.007402 |
| P42123 | - Ldhb | L-lactate dehydrogenase B chain | 0.82 | 0.000082 | 0.001513 |
| P08081 | - Clta | Clathrin light chain A | 0.82 | 0.0006031 | 0.005256 |
| Q9WUC4 | - Atox1 | Copper transport protein ATOX1 | 0.82 | 0.0353984 | 0.088904 |
| P97697 | - Impa1 | Inositol monophosphatase 1 | 0.82 | 0.012621 | 0.041154 |
| Q9JI03 | - Col5a1 | Collagen alpha-1(V) chain | 0.82 | 0.0415892 | 0.100779 |
| Q5XI22 | - Acat2 | Acetyl-CoA acetyltransferase, cytosolic | 0.82 | 0.0002279 | 0.003034 |
| P06302 | - Ptma | Prothymosin alpha | 0.82 | 0.0014951 | 0.009267 |
| P11960 | - Bckdha | 2-oxoisovalerate dehydrogenase subunit alpha, mitochondrial (Fragment) | 0.82 | 0.0132905 | 0.042403 |
| P38659 | - Pdia4 | Protein disulfide-isomerase A4 | 0.82 | 0.0144377 | 0.019502 |
| P20069 | - Pmpca | Mitochondrial-processing peptidase subunit alpha | 0.82 | 0.0074923 | 0.028568 |
| P00507 | - Got2 | Aspartate aminotransferase, mitochondrial | 0.82 | 0.0005887 | 0.005162 |
| Q6PDU7 | - Atp5l | ATP synthase subunit g, mitochondrial | 0.82 | 0.0003393 | 0.003668 |
| P08461 | - Dlat | Dihydrolipoyllysine-residue acetyltransferase component of pyruvate dehydrogenase complex, mitochondrial | 0.82 | 0.0183297 | 0.054347 |
| Q9ESN0 | - Fam129a | Protein Niban | 0.81 | 0.0016023 | 0.00966 |
| P70615 | - Lmnb1 | Lamin-B1 | 0.81 | 0.0067297 | 0.0263 |
| P80254 | - Ddt | D-dopachrome decarboxylase | 0.81 | 0.0324687 | 0.083078 |
| Q6QD51 | - Ccdc80 | Coiled-coil domain-containing protein 80 | 0.81 | 0.0155696 | 0.048252 |
| Q6P7A9 | - Gaa | Lysosomal alpha-glucosidase | 0.81 | 0.0009507 | 0.007084 |
| Q5I0D1 | - Glod4 | Glyoxalase domain-containing protein 4 | 0.81 | 0.0000244 | 0.000946 |
| P17046 | - Lamp2 | Lysosome-associated membrane glycoprotein 2 | 0.81 | 0.0183831 | 0.054393 |
| Q5XHZ0 | - Trap1 | Heat shock protein 75 kDa, mitochondrial | 0.81 | 0.0004147 | 0.004158 |
| Q62868 | - Rock2 | Rho-associated protein kinase 2 | 0.81 | 0.0136241 | 0.043153 |
| Q810U0 | - Ccdc50 | Coiled-coil domain-containing protein 50 | 0.81 | 0.0329995 | 0.084286 |
| Q07936 | - Anxa2 | Annexin A2 | 0.81 | 0.0001686 | 0.002475 |
| P09456 | - Prkar1a | cAMP-dependent protein kinase type I-alpha regulatory subunit | 0.81 | 0.0091928 | 0.032802 |
| Q920D2 | - Dhfr | Dihydrofolate reductase | 0.81 | 0.0468536 | 0.109199 |
| Q5XFX0 | - Tagln2 | Transgelin-2 | 0.81 | 0.0035694 | 0.016884 |
| P20070 | - Cyb5r3 | NADH-cytochrome b5 reductase 3 | 0.81 | 0.0014827 | 0.009267 |
| P19511 | - Atp5f1 | ATP synthase F(0) complex subunit B1, mitochondrial | 0.8 | 0.0000492 | 0.001276 |
| Q99ML5 | - Pcyox1 | Prenylcysteine oxidase | 0.8 | 0.0125499 | 0.041109 |
| Q9QXU8 | - Dync1li1 | Cytoplasmic dynein 1 light intermediate chain 1 | 0.8 | 0.0005829 | 0.005142 |
| Q62733 | - Tmpo | Lamina-associated polypeptide 2, isoform beta | 0.8 | 0.005996 | 0.024152 |
| Q9EPB1 | - Dpp7 | Dipeptidyl peptidase 2 | 0.8 | 0.0029135 | 0.014592 |
| O88994 | - Mar-02 | Mitochondrial amidoxime reducing component 2 | 0.8 | 0.020214 | 0.058019 |
| Q8CG09 | - Abcc1 | Multidrug resistance-associated protein 1 | 0.8 | 0.0316521 | 0.081569 |
| P50137 | - Tkt | Transketolase | 0.8 | 0.0033514 | 0.016227 |
| Q64375 | - P3h4 | Synaptonemal complex protein SC65 (Fragment) | 0.8 | 0.0130969 | 0.042227 |
| O88767 | - Park7 | Protein deglycase DJ-1 | 0.8 | 0.0005405 | 0.004888 |
| P63281 | - Ube2i | SUMO-conjugating enzyme UBC9 | 0.8 | 0.0002356 | 0.003052 |
| Q9JLT0 | - Myh10 | Myosin-10 | 0.8 | 0.0074501 | 0.028493 |
| O35303 | - Dnm1l | Dynamin-1-like protein | 0.79 | 0.000729 | 0.005923 |
| Q642C0 | - Dnajc8 | DnaJ homolog subfamily C member 8 | 0.79 | 0.0003905 | 0.004011 |
| Q07205 | - Eif5 | Eukaryotic translation initiation factor 5 | 0.79 | 0.0072797 | 0.028065 |
| P49911 | - Anp32a | Acidic leucine-rich nuclear phosphoprotein 32 family member A | 0.79 | 0.0006473 | 0.005476 |
| Q6AXT0 | - Mrpl37 | 39S ribosomal protein L37, mitochondrial | 0.79 | 0.025243 | 0.06901 |
| Q5XIG0 | - Nudt9 | ADP-ribose pyrophosphatase, mitochondrial | 0.79 | 0.0349921 | 0.08815 |
| P85972 | - Vcl | Vinculin | 0.79 | 0.0417693 | 0.100779 |
| P21571 | - Atp5j | ATP synthase-coupling factor 6, mitochondrial | 0.79 | 0.0341873 | 0.086704 |
| P80386 | - Prkab1 | 5'-AMP-activated protein kinase subunit beta-1 | 0.79 | 0.0241881 | 0.066633 |
| Q63598 | - Pls3 | Plastin-3 | 0.79 | 0.0176144 | 0.05288 |
| A2RUW1 | - Tollip | Toll-interacting protein | 0.79 | 0.015506 | 0.048159 |
| E9PT23 | - Slc38a10 | Putative sodium-coupled neutral amino acid transporter 10 | 0.79 | 0.0086947 | 0.031814 |
| P21775 | - Acaa1a | 3-ketoacyl-CoA thiolase A, peroxisomal | 0.79 | 0.0008882 | 0.00673 |
| Q5GFD9 | - Impact | Protein IMPACT | 0.79 | 0.0000745 | 0.001488 |
| P34926 | - Map1a | Microtubule-associated protein 1A | 0.79 | 0.0015535 | 0.009539 |
| P47860 | - Pfkp | ATP-dependent 6-phosphofructokinase, platelet type | 0.79 | 0.0000319 | 0.001032 |
| P35571 | - Gpd2 | Glycerol-3-phosphate dehydrogenase, mitochondrial | 0.79 | 0.0089304 | 0.032429 |
| Q63184 | - Eif2ak2 | Interferon-induced, double-stranded RNA-activated protein kinase | 0.79 | 0.0032215 | 0.015703 |
| Q5XI78 | - Ogdh | 2-oxoglutarate dehydrogenase, mitochondrial | 0.79 | 0.0003847 | 0.003983 |
| Q920L2 | - Sdha | Succinate dehydrogenase [ubiquinone] flavoprotein subunit, mitochondrial | 0.79 | 0.0010525 | 0.007419 |
| Q9Z2F5 | - Ctbp1 | C-terminal-binding protein 1 | 0.79 | 0.0221039 | 0.06148 |
| Q32KJ6 | - Galns | N-acetylgalact amine-6-sulfatase | 0.79 | 0.0026002 | 0.013833 |
| Q9Z2Z8 | - Dhcr7 | 7-dehydrocholesterol reductase | 0.79 | 0.0448463 | 0.106167 |
| Q704S8 | - Crat | Carnitine O-acetyltransferase | 0.78 | 0.0168116 | 0.05111 |
| Q80Z70 | - Sel1l | Protein sel-1 homolog 1 | 0.78 | 0.0060134 | 0.024154 |
| Q99NA5 | - Idh3a | Isocitrate dehydrogenase [NAD] subunit alpha, mitochondrial | 0.78 | 0.0012564 | 0.008482 |
| P0C5W1 | - Map1s | Microtubule-associated protein 1S | 0.78 | 0.0154979 | 0.048159 |
| P61959 | - Sumo2 | Small ubiquitin-related modifier 2 | 0.78 | 0.0000643 | 0.001376 |
| Q64057 | - Aldh7a1 | Alpha-aminoadipic semialdehyde dehydrogenase | 0.78 | 0.0008614 | 0.00666 |
| Q62940 | - Nedd4 | E3 ubiquitin-protein ligase NEDD4 | 0.78 | 0.0002572 | 0.003069 |
| F1LP64 | - Trip12 | E3 ubiquitin-protein ligase TRIP12 | 0.78 | 0.0055348 | 0.022955 |
| P97521 | - Slc25a20 | Mitochondrial carnitine/acylcarnitine carrier protein | 0.78 | 0.0154279 | 0.048159 |
| Q9ER34 | - Aco2 | Aconitate hydratase, mitochondrial | 0.78 | 0.0005769 | 0.005121 |
| P85125 | - Cavin1 | Polymerase I and transcript release factor | 0.77 | 0.0007397 | 0.005976 |
| Q01205 | - Dlst | Dihydrolipoyllysine-residue succinyltransferase component of 2-oxoglutarate dehydrogenase complex, mitochondrial | 0.77 | 0.021727 | 0.060785 |
| P19836 | - Pcyt1a | Choline-phosphate cytidylyltransferase A | 0.77 | 0.0045096 | 0.019953 |
| P47942 | - Dpysl2 | Dihydropyrimidinase-related protein 2 | 0.77 | 0.0000575 | 0.001342 |
| Q5XIC2 | - Ecsit | Evolutionarily conserved signaling intermediate in Toll pathway, mitochondrial | 0.77 | 0.010009 | 0.034807 |
| P32198 | - Cpt1a | Carnitine O-palmitoyltransferase 1, liver isoform | 0.77 | 0.0347431 | 0.087804 |
| Q8VHF5 | - Cs | Citrate synthase, mitochondrial | 0.77 | 0.0001331 | 0.002103 |
| P05708 | - Hk1 | Hexokinase-1 | 0.77 | 0.0000347 | 0.001061 |
| P04166 | - Cyb5b | Cytochrome b5 type B | 0.77 | 0.0051621 | 0.022027 |
| P15999 | - Atp5a1 | ATP synthase subunit alpha, mitochondrial | 0.77 | 0.0003735 | 0.00392 |
| Q62696 | - Dlg1 | Disks large homolog 1 | 0.77 | 0.0092286 | 0.032848 |
| P05942 | - S100a4 | Protein S100-A4 | 0.77 | 0.000385 | 0.003983 |
| Q2TA68 | - Opa1 | Dynamin-like 120 kDa protein, mitochondrial | 0.77 | 0.0205501 | 0.058867 |
| P0C1X8 | - Aak1 | AP2-associated protein kinase 1 | 0.77 | 0.0480759 | 0.111146 |
| Q64361 | - Lxn | Latexin | 0.77 | 0.026163 | 0.070986 |
| P42676 | - Nln | Neurolysin, mitochondrial | 0.76 | 0.0027773 | 0.014366 |
| Q06647 | - Atp5o | ATP synthase subunit O, mitochondrial | 0.76 | 0.0053029 | 0.022297 |
| P30009 | - Marcks | Myristoylated alanine-rich C-kinase substrate | 0.76 | 0.0052503 | 0.022183 |
| Q9Z1E1 | - Flot1 | Flotillin-1 | 0.76 | 0.0212713 | 0.060095 |
| Q5XIT9 | - Mccc2 | Methylcrotonoyl-CoA carboxylase beta chain, mitochondrial | 0.76 | 0.0000958 | 0.00166 |
| Q6AYE2 | - Sh3glb1 | Endophilin-B1 | 0.76 | 0.0378772 | 0.093266 |
| P97700 | - Slc25a11 | Mitochondrial 2-oxoglutarate/malate carrier protein | 0.76 | 0.0057248 | 0.023444 |
| Q6P6R2 | - Dld | Dihydrolipoyl dehydrogenase, mitochondrial | 0.76 | 0.0027338 | 0.014192 |
| P32089 | - Slc25a1 | Tricarboxylate transport protein, mitochondrial | 0.76 | 0.002007 | 0.011318 |
| O70351 | - Hsd17b10 | 3-hydroxyacyl-CoA dehydrogenase type-2 | 0.76 | 0.004254 | 0.019237 |
| P26772 | - Hspe1 | 10 kDa heat shock protein, mitochondrial | 0.76 | 0.036988 | 0.091863 |
| P55260 | - Anxa4 | Annexin A4 | 0.76 | 0.0055711 | 0.022955 |
| Q2EJA0 | - Yap1 | Transcriptional coactivator YAP1 | 0.75 | 0.0134712 | 0.042763 |
| P04041 | - Gpx1 | Glutathione peroxidase 1 | 0.75 | 0.0115888 | 0.038804 |
| Q68FP1 | - Gsn | Gelsolin | 0.75 | 0.0000217 | 0.000934 |
| Q924K2 | - Faf1 | FAS-associated factor 1 | 0.75 | 0.0322683 | 0.082713 |
| Q4QQV3 | - Fam162a | Protein FAM162A | 0.75 | 0.0476892 | 0.11043 |
| P00173 | - Cyb5a | Cytochrome b5 | 0.75 | 0.0015601 | 0.009539 |
| P15865 | - Hist1h1e | Histone H1.4 | 0.75 | 0.014317 | 0.045248 |
| Q3B8P0 | - Parl | Presenilins-associated rhomboid-like protein, mitochondrial | 0.75 | 0.0414244 | 0.100622 |
| P48037 | - Anxa6 | Annexin A6 | 0.75 | 0.0000279 | 0.00099 |
| Q10758 | - Krt8 | Keratin, type II cytoskeletal 8 | 0.75 | 0.0000942 | 0.00166 |
| Q4V8H8 | - Ehd2 | EH domain-containing protein 2 | 0.75 | 0.0004423 | 0.004269 |
| P97571 | - Capn1 | Calpain-1 catalytic subunit | 0.74 | 0.0000103 | 0.000743 |
| Q9Z1H9 | - Cavin3 | Protein kinase C delta-binding protein | 0.74 | 0.004402 | 0.019659 |
| P06214 | - Alad | Delta-aminolevulinic acid dehydratase | 0.74 | 0.0303985 | 0.07919 |
| Q5U2Q3 | - N/A | Ester hydrolase C11orf54 homolog | 0.74 | 0.0463222 | 0.108842 |
| Q68FU3 | - Etfb | Electron transfer flavoprotein subunit beta | 0.74 | 0.0003606 | 0.003813 |
| P23785 | - Grn | Granulins | 0.74 | 0.0207259 | 0.059134 |
| Q62638 | - Glg1 | Golgi apparatus protein 1 | 0.74 | 0.0050421 | 0.021643 |
| P30904 | - Mif | Macrophage migration inhibitory factor | 0.74 | 0.013202 | 0.042376 |
| P45953 | - Acadvl | Very long-chain specific acyl-CoA dehydrogenase, mitochondrial | 0.74 | 0.0000231 | 0.000934 |
| P38652 | - Pgm1 | Phosphoglucomutase-1 | 0.74 | 0.0006844 | 0.005664 |
| P21913 | - Sdhb | Succinate dehydrogenase [ubiquinone] iron-sulfur subunit, mitochondrial | 0.74 | 0.020986 | 0.059522 |
| P10688 | - Plcd1 | 1-phosphatidylinositol 4,5-bisphosphate phosphodiesterase delta-1 | 0.74 | 0.0040607 | 0.018656 |
| Q8VID1 | - Dhrs4 | Dehydrogenase/reductase SDR family member 4 | 0.74 | 0.0295825 | 0.077627 |
| P08010 | - Gstm2 | Glutathione S-transferase Mu 2 | 0.74 | 0.0001534 | 0.002362 |
| P10719 | - Atp5b | ATP synthase subunit beta, mitochondrial | 0.74 | 0.0006633 | 0.005546 |
| P52873 | - Pc | Pyruvate carboxylase, mitochondrial | 0.73 | 0.0002753 | 0.003092 |
| Q4KM35 | - Psmb10 | Proteasome subunit beta type-10 | 0.73 | 0.040488 | 0.098848 |
| P49432 | - Pdhb | Pyruvate dehydrogenase E1 component subunit beta, mitochondrial | 0.73 | 0.0000004 | 0.000294 |
| P28042 | - Ssbp1 | Single-stranded DNA-binding protein, mitochondrial | 0.73 | 0.001851 | 0.01069 |
| Q63663 | - Gbp2 | Guanylate-binding protein 1 | 0.73 | 0.0071477 | 0.027704 |
| Q4V7F2 | - Creld1 | Cysteine-rich with EGF-like domain protein 1 | 0.73 | 0.0000831 | 0.001513 |
| B0BNF1 | - Sep-08 | Septin-8 | 0.73 | 0.0156106 | 0.048275 |
| P36201 | - Crip2 | Cysteine-rich protein 2 | 0.73 | 0.009093 | 0.032689 |
| Q9Z2S9 | - Flot2 | Flotillin-2 | 0.73 | 0.0010387 | 0.007402 |
| P02454 | - Col1a1 | Collagen alpha-1(I) chain | 0.73 | 0.0019132 | 0.010874 |
| Q5I0C3 | - Mccc1 | Methylcrotonoyl-CoA carboxylase subunit alpha, mitochondrial | 0.73 | 0.0358732 | 0.089558 |
| Q99068 | - Lrpap1 | Alpha-2-macroglobulin receptor-associated protein | 0.73 | 0.0014664 | 0.009208 |
| Q9QZK5 | - Htra1 | Serine protease HTRA1 | 0.73 | 0.0181115 | 0.053811 |
| Q5PQM0 | - Tmem168 | Transmembrane protein 168 | 0.72 | 0.0196592 | 0.057111 |
| P63095 | - Gnas | Guanine nucleotide-binding protein G(s) subunit alpha isoforms short | 0.72 | 0.0113979 | 0.038565 |
| P63045 | - Vamp2 | Vesicle-associated membrane protein 2 | 0.72 | 0.0488765 | 0.112635 |
| P15651 | - Acads | Short-chain specific acyl-CoA dehydrogenase, mitochondrial | 0.72 | 0.0064452 | 0.025603 |
| P07092 | - Serpine2 | Glia-derived nexin | 0.72 | 0.0399649 | 0.097737 |
| P56522 | - Fdxr | NADPH: adrenodoxin oxidoreductase, mitochondrial | 0.72 | 0.0162405 | 0.049795 |
| Q9JHY2 | - Sfxn3 | Sideroflexin-3 | 0.72 | 0.0018098 | 0.010554 |
| P14604 | - Echs1 | Enoyl-CoA hydratase, mitochondrial | 0.72 | 0.0030698 | 0.0151 |
| P35434 | - Atp5d | ATP synthase subunit delta, mitochondrial | 0.72 | 0.0049924 | 0.021494 |
| P97532 | - Mpst | 3-mercaptopyruvate sulfurtransferase | 0.72 | 0.0004222 | 0.004158 |
| Q6P747 | - Hp1bp3 | Heterochromatin protein 1-binding protein 3 | 0.72 | 0.0000947 | 0.00166 |
| Q8CG45 | - Akr7a2 | Aflatoxin B1 aldehyde reductase member 2 | 0.71 | 0.0028299 | 0.014433 |
| P53534 | - Pygb | Glycogen phosphorylase, brain form (Fragment) | 0.71 | 0.0000647 | 0.001376 |
| P47858 | - Pfkm | ATP-dependent 6-phosphofructokinase, muscle type | 0.71 | 0.0001908 | 0.002638 |
| P10888 | - Cox4i1 | Cytochrome c oxidase subunit 4 isoform 1, mitochondrial | 0.71 | 0.0012368 | 0.008389 |
| Q64232 | - Tecr | Very-long-chain enoyl-CoA reductase | 0.71 | 0.0129254 | 0.041862 |
| Q6Q0N3 | - Nt5dc2 | 5'-nucleotidase domain-containing protein 2 | 0.71 | 0.0022816 | 0.012571 |
| P13803 | - Etfa | Electron transfer flavoprotein subunit alpha, mitochondrial | 0.71 | 0.0000466 | 0.001265 |
| Q75WE7 | - Vwa5a | von Willebrand factor A domain-containing protein 5A | 0.71 | 0.0001544 | 0.002362 |
| D4ACN8 | - Plgrkt | Plasminogen receptor (KT) | 0.71 | 0.0009718 | 0.007143 |
| P04182 | - Oat | Ornithine aminotransferase, mitochondrial | 0.71 | 0.0047211 | 0.020572 |
| P27867 | - Sord | Sorbitol dehydrogenase | 0.71 | 0.0004821 | 0.004561 |
| Q4KM98 | - Mff | Mitochondrial fission factor | 0.71 | 0.0435993 | 0.104146 |
| P28077 | - Psmb9 | Proteasome subunit beta type-9 | 0.7 | 0.0413773 | 0.100622 |
| Q4TU93 | - Mrc2 | C-type mannose receptor 2 | 0.7 | 0.0008429 | 0.006642 |
| O54748 | - Stk3 | Serine/threonine-protein kinase 3 | 0.7 | 0.0076585 | 0.029058 |
| P60825 | - Cirbp | Cold-inducible RNA-binding protein | 0.7 | 0.0350027 | 0.08815 |
| Q63797 | - Psme1 | Proteasome activator complex subunit 1 | 0.7 | 0.0002322 | 0.003036 |
| Q6AY84 | - Scrn1 | Secernin-1 | 0.7 | 0.0018914 | 0.010836 |
| Q9JJ22 | - Erap1 | Endoplasmic reticulum aminopeptidase 1 | 0.7 | 0.0009802 | 0.007155 |
| P41562 | - Idh1 | Isocitrate dehydrogenase [NADP] cytoplasmic | 0.7 | 0.0000098 | 0.000743 |
| P15650 | - Acadl | Long-chain specific acyl-CoA dehydrogenase, mitochondrial | 0.7 | 0.0000598 | 0.001342 |
| P0C2X9 | - Aldh4a1 | Delta-1-pyrroline-5-carboxylate dehydrogenase, mitochondrial | 0.7 | 0.0003332 | 0.00363 |
| P11240 | - Cox5a | Cytochrome c oxidase subunit 5A, mitochondrial | 0.7 | 0.0001077 | 0.001801 |
| P14882 | - Pcca | Propionyl-CoA carboxylase alpha chain, mitochondrial | 0.7 | 0.0000914 | 0.001643 |
| Q60587 | - Hadhb | Trifunctional enzyme subunit beta, mitochondrial | 0.7 | 0.0003499 | 0.003755 |
| Q9JIM0 | - Mre11 | Double-strand break repair protein MRE11A | 0.7 | 0.0083976 | 0.030884 |
| Q64428 | - Hadha | Trifunctional enzyme subunit alpha, mitochondrial | 0.69 | 0.0000443 | 0.001232 |
| Q810F4 | - Fam3c | Protein FAM3C | 0.69 | 0.0013938 | 0.009069 |
| Q63321 | - Plod1 | Procollagen-lysine,2-oxoglutarate 5-dioxygenase 1 | 0.69 | 0.0000215 | 0.000934 |
| P21396 | - Maoa | Amine oxidase [flavin-containing] A | 0.69 | 0.0000825 | 0.001513 |
| P25093 | - Fah | Fumarylacetoacetase | 0.69 | 0.00402 | 0.018528 |
| Q6AXU6 | - JPT1 | Hematological and neurological expressed 1 protein | 0.69 | 0.0014327 | 0.009069 |
| Q9Z327 | - Synpo | Synaptopodin | 0.69 | 0.0039664 | 0.01834 |
| P56574 | - Idh2 | Isocitrate dehydrogenase [NADP], mitochondrial | 0.69 | 0.0001601 | 0.002398 |
| Q7TQ16 | - Uqcrq | Cytochrome b-c1 complex subunit 8 | 0.69 | 0.047405 | 0.110127 |
| Q9JI85 | - Nucb2 | Nucleobindin-2 | 0.68 | 0.0186665 | 0.054822 |
| P11951 | - Cox6c2 | Cytochrome c oxidase subunit 6C-2 | 0.68 | 0.0200809 | 0.057753 |
| D3ZAF6 | - Atp5j2 | ATP synthase subunit f, mitochondrial | 0.68 | 0.0032847 | 0.015957 |
| Q7TPB4 | - Cd276 | CD276 antigen | 0.68 | 0.0001779 | 0.002533 |
| P41350 | - Cav1 | Caveolin-1 | 0.68 | 0.0016633 | 0.009966 |
| Q10739 | - Mmp14 | Matrix metalloproteinase-14 | 0.68 | 0.0013451 | 0.008914 |
| Q00238 | - Icam1 | Intercellular adhesion molecule 1 | 0.68 | 0.0005359 | 0.004877 |
| Q9Z0J5 | - Txnrd2 | Thioredoxin reductase 2, mitochondrial | 0.68 | 0.0006278 | 0.005399 |
| P12075 | - Cox5b | Cytochrome c oxidase subunit 5B, mitochondrial | 0.68 | 0.0041743 | 0.018996 |
| O88775 | - Emb | Embigin | 0.68 | 0.0206963 | 0.059134 |
| B0BNA5 | - Cotl1 | Coactosin-like protein | 0.68 | 0.0089586 | 0.03245 |
| O35854 | - Bcat2 | Branched-chain-amino-acid aminotransferase, mitochondrial | 0.68 | 0.0035203 | 0.016762 |
| P15978 | - RT1-Aw2 | Class I histocompatibility antigen, Non-RT1.A alpha-1 chain | 0.68 | 0.0041685 | 0.018996 |
| P23965 | - Eci1 | Enoyl-CoA delta isomerase 1, mitochondrial | 0.67 | 0.0000586 | 0.001342 |
| P18886 | - Cpt2 | Carnitine O-palmitoyltransferase 2, mitochondrial | 0.67 | 0.0020404 | 0.011461 |
| P04906 | - Gstp1 | Glutathione S-transferase P | 0.67 | 0.0132907 | 0.042403 |
| P97608 | - Oplah | 5-oxoprolinase | 0.67 | 0.0046135 | 0.020226 |
| Q9ER24 | - Atxn10 | Ataxin-10 | 0.67 | 0.0153647 | 0.048136 |
| P14841 | - Cst3 | Cystatin-C | 0.67 | 0.0215712 | 0.060568 |
| P97546 | - Nptn | Neuroplastin | 0.67 | 0.002868 | 0.014471 |
| P97852 | - Hsd17b4 | Peroxisomal multifunctional enzyme type 2 | 0.67 | 0.000066 | 0.001376 |
| P06536 | - Nr3c1 | Glucocorticoid receptor | 0.67 | 0.0385047 | 0.094488 |
| P18437 | - Hmgn2 | Non-histone chromosomal protein HMG-17 | 0.66 | 0.0081645 | 0.030574 |
| P50123 | - Enpep | Glutamyl aminopeptidase | 0.66 | 0.0006071 | 0.005259 |
| P10860 | - Glud1 | Glutamate dehydrogenase 1, mitochondrial | 0.66 | 0.0002313 | 0.003036 |
| P52759 | - Rida | Ribonuclease UK114 | 0.65 | 0.0008711 | 0.006679 |
| P26453 | - Bsg | Basigin | 0.65 | 0.0012228 | 0.008389 |
| Q5XIC0 | - Eci2 | Enoyl-CoA delta isomerase 2, mitochondrial | 0.65 | 0.0000079 | 0.000743 |
| Q5XIE6 | - Hibch | 3-hydroxyisobutyryl-CoA hydrolase, mitochondrial | 0.65 | 0.0358018 | 0.089536 |
| Q6P7C7 | - Gpnmb | Transmembrane glycoprotein NMB | 0.65 | 0.0001072 | 0.001801 |
| P06760 | - Gusb | Beta-glucuronidase | 0.65 | 0.0074165 | 0.02844 |
| Q6P0K8 | - Jup | Junction plakoglobin | 0.65 | 0.0005066 | 0.00467 |
| P00406 | - Mtco2 | Cytochrome c oxidase subunit 2 | 0.65 | 0.0001418 | 0.002216 |
| P15684 | - Anpep | Aminopeptidase N | 0.64 | 0.0218193 | 0.060821 |
| P28037 | - Aldh1l1 | Cytosolic 10-formyltetrahydrofolate dehydrogenase | 0.64 | 0.0106161 | 0.036371 |
| Q920G2 | - Slc9a3r2 | Na(+)/H(+) exchange regulatory cofactor NHE-RF2 | 0.64 | 0.0119657 | 0.039556 |
| P84817 | - Fis1 | Mitochondrial fission 1 protein | 0.64 | 0.0009579 | 0.007101 |
| P21708 | - Mapk3 | Mitogen-activated protein kinase 3 | 0.64 | 0.001231 | 0.008389 |
| P70584 | - Acadsb | Short/branched chain specific acyl-CoA dehydrogenase, mitochondrial | 0.64 | 0.0002386 | 0.003063 |
| P07150 | - Anxa1 | Annexin A1 | 0.63 | 0.0000119 | 0.000743 |
| Q4G075 | - Serpinb1a | Leukocyte elastase inhibitor A | 0.63 | 0.0018797 | 0.010812 |
| P35053 | - Gpc1 | Glypican-1 | 0.63 | 0.0001877 | 0.002627 |
| P13596 | - Ncam1 | Neural cell adhesion molecule 1 | 0.63 | 0.0015943 | 0.00966 |
| Q9QX69 | - Lancl1 | LanC-like protein 1 | 0.63 | 0.0007704 | 0.006155 |
| P29266 | - Hibadh | 3-hydroxyisobutyrate dehydrogenase, mitochondrial | 0.63 | 0.000031 | 0.001032 |
| Q08850 | - Stx4 | Syntaxin-4 | 0.63 | 0.0002746 | 0.003092 |
| Q1AAU6 | - Asap1 | Arf-GAP with SH3 domain, ANK repeat and PH domain-containing protein 1 | 0.63 | 0.0057536 | 0.023444 |
| P07154 | - Ctsl | Cathepsin L1 | 0.63 | 0.0018394 | 0.010666 |
| Q6MG60 | - Ddah2 | N(G),N(G)-dimethylarginine dimethylaminohydrolase 2 | 0.63 | 0.0000713 | 0.001465 |
| P70550 | - Rab8b | Ras-related protein Rab-8B | 0.63 | 0.0016055 | 0.00966 |
| Q9Z252 | - Lin7b | Protein lin-7 homolog B | 0.62 | 0.0109877 | 0.037353 |
| P29411 | - Ak3 | GTP:AMP phosphotransferase AK3, mitochondrial | 0.62 | 0.000036 | 0.00108 |
| Q9ERB4 | - Vcan | Versican core protein (Fragments) | 0.62 | 0.0116038 | 0.038804 |
| Q5XI42 | - Aldh3b1 | Aldehyde dehydrogenase family 3 member B1 | 0.62 | 0.0078887 | 0.029771 |
| P08011 | - Mgst1 | Microsomal glutathione S-transferase 1 | 0.62 | 0.0199485 | 0.05775 |
| P18614 | - Itga1 | Integrin alpha-1 | 0.61 | 0.0246665 | 0.067821 |
| P18297 | - Spr | Sepiapterin reductase | 0.61 | 0.0009427 | 0.007061 |
| Q9QWN8 | - Sptbn2 | Spectrin beta chain, non-erythrocytic 2 | 0.61 | 0.0019092 | 0.010874 |
| P12007 | - Ivd | Isovaleryl-CoA dehydrogenase, mitochondrial | 0.61 | 0.0000446 | 0.001232 |
| Q4G017 | - Nisch | Nischarin | 0.61 | 0.0407536 | 0.099328 |
| P17764 | - Acat1 | Acetyl-CoA acetyltransferase, mitochondrial | 0.61 | 0.0009736 | 0.007143 |
| Q5BJK8 | - Golim4 | Golgi integral membrane protein 4 | 0.6 | 0.000066 | 0.001376 |
| Q9WVK7 | - Hadh | Hydroxyacyl-coenzyme A dehydrogenase, mitochondrial | 0.6 | 0.0000067 | 0.000743 |
| Q8K5A9 | - Nradd | Death domain-containing membrane protein NRADD | 0.6 | 0.0004733 | 0.004507 |
| P04904 | - Gsta3 | Glutathione S-transferase alpha-3 | 0.59 | 0.0012798 | 0.0086 |
| Q9EPH2 | - Marcksl1 | MARCKS-related protein | 0.59 | 0.0038476 | 0.017866 |
| P11915 | - Scp2 | Non-specific lipid-transfer protein | 0.59 | 0.0002519 | 0.003069 |
| P07633 | - Pccb | Propionyl-CoA carboxylase beta chain, mitochondrial | 0.58 | 0.0000289 | 0.00099 |
| P11505 | - Atp2b1 | Plasma membrane calcium-transporting ATPase 1 | 0.58 | 0.0000227 | 0.000934 |
| P16086 | - Sptan1 | Spectrin alpha chain, non-erythrocytic 1 | 0.58 | 0.0000094 | 0.000743 |
| D4A1J4 | - Bdh2 | 3-hydroxybutyrate dehydrogenase type 2 | 0.58 | 0.0094715 | 0.03363 |
| P30839 | - Aldh3a2 | Fatty aldehyde dehydrogenase | 0.58 | 0.0004343 | 0.00422 |
| P16391 | - N/A | RT1 class I histocompatibility antigen, AA alpha chain | 0.58 | 0.0048209 | 0.020944 |
| Q9JJ19 | - Slc9a3r1 | Na(+)/H(+) exchange regulatory cofactor NHE-RF1 | 0.57 | 0.0000786 | 0.001502 |
| P12369 | - Prkar2b | cAMP-dependent protein kinase type II-beta regulatory subunit | 0.57 | 0.004501 | 0.019953 |
| Q6JE36 | - Ndrg1 | Protein NDRG1 | 0.56 | 0.0058849 | 0.023821 |
| Q9WU82 | - Ctnnb1 | Catenin beta-1 | 0.56 | 0.0000517 | 0.001304 |
| Q62627 | - Pawr | PRKC apoptosis WT1 regulator protein | 0.55 | 0.0465606 | 0.108942 |
| P13437 | - Acaa2 | 3-ketoacyl-CoA thiolase, mitochondrial | 0.55 | 0.0000607 | 0.001342 |
| Q02253 | - Aldh6a1 | Methylmalonate-semialdehyde dehydrogenase [acylating], mitochondrial | 0.55 | 0.0000017 | 0.000621 |
| Q5U4F3 | - Fam107b | Protein FAM107B | 0.55 | 0.0026386 | 0.013899 |
| P11884 | - Aldh2 | Aldehyde dehydrogenase, mitochondrial | 0.54 | 0.0000111 | 0.000743 |
| P52631 | - Stat3 | Signal transducer and activator of transcription 3 | 0.54 | 0.000033 | 0.001032 |
| Q5U2T3 | - Spats2l | SPATS2-like protein | 0.52 | 0.0001881 | 0.002627 |
| Q5RKH6 | - Os9 | Protein Os-9 | 0.51 | 0.0000169 | 0.000895 |
| Q62651 | - Ech1 | Delta (3,5)-Delta (2,4)-dienoyl-CoA isomerase, mitochondrial | 0.5 | 0.0014289 | 0.009069 |
| Q64591 | - Decr1 | 2,4-dienoyl-CoA reductase, mitochondrial | 0.49 | 0.0002485 | 0.003069 |
| O08628 | - Pcolce | Procollagen C-endopeptidase enhancer 1 | 0.49 | 0.0000142 | 0.000795 |
| P04762 | - Cat | Catalase | 0.47 | 0.0000000 | 0.000004 |
| Q63028 | - Add1 | Alpha-adducin | 0.44 | 0.0000762 | 0.001501 |
| P08430 | - Ugt1a6 | UDP-glucuronosyltransferase 1-6 | 0.43 | 0.0000274 | 0.00099 |
| O35276 | - Nrp2 | Neuropilin-2 | 0.43 | 0.0074137 | 0.02844 |
| Q8R4C0 | - Capn5 | Calpain-5 | 0.39 | 0.0025916 | 0.013833 |
| Q62847 | - Add3 | Gamma-adducin | 0.37 | 0.0052223 | 0.022152 |
| Q8VIF7 | - Selenbp1 | Selenium-binding protein 1 | 0.34 | 0.0000377 | 0.001107 |
| P11883 | - Aldh3a1 | Aldehyde dehydrogenase, dimeric NADP-preferring | 0.22 | 0.0000075 | 0.000743 |

Supplementary table 2. Cell-lysate proteins regulated by TGF-β1 are involved in 42 KEGG pathways. Proteins are listed in order of TGF-β1/control ratios; **“•**”: induced by TGF-β1; **“•**”: repressed by TGF-β1**.**

| KEGG pathways | Proteins significantly regulated by TGF-β1 (TGF-β1/control ratio) |
| --- | --- |
| Ribosome  (most significant) | - Rps30 (2.07), Rpl36a (1.95), Rps23 (1.89), Rpl35a (1.71), Rps24 (1.6), Rpl37 (1.56), Rpl23a (1.56), Rpl27a (1.54), Rps3 (1.52), Rplp0 (1.5), Rps10 (1.5), Rpl35 (1.5), Rps11 (1.47), Rps14 (1.46), Rpl17 (1.45), Rpl27 (1.45), Rpl32 (1.42), Rpsa (1.42), Rpl18A (1.42), Rps16 (1.41), Rps17 (1.41), Rpl22 (1.37), Rps13 (1.36), Rpl18 (1.35), Rps29 (1.34), Rpl26 (1.34), Rps2 (1.33), Rps18 (1.33), Rpl36 (1.33), Rpl6 (1.33), Rps6 (1.33), Rpl13a (1.33), Rps19 (1.32), Rps8 (1.32), Rpl23 (1.32), Rpl13 (1.31), Rps15 (1.31), Rpl38 (1.31), Rpl5 (1.31), Rplp2 (1.3), Rpl10 (1.3), Rpl7 (1.3), Rpl29 (1.3), Rpl15 (1.29), Rps27l (1.29), Rpl12 (1.29), Rps4X (1.28), Rps26 (1.28), Rpl30 (1.27), Rpl3 (1.27), Rpl7a (1.27), Rpl10a (1.27), Rpl21 (1.27), Rps3a (1.26), Rpl34 (1.26), Rpl8 (1.26), Rps20 (1.26), Rpl24 (1.26), Rpl4 (1.25), Rpl9 (1.23), Rps9 (1.23), Rps15a (1.22), Rpl28 (1.22), Rpl19 (1.19), Rpl14 (1.18), Rps27 (1.18), Rps21 (1.17), Rps28 (1.14). - Mrpl3 (0.84) |
| Biosynthesis of antibiotics | - Bcat1 (1.94), Psph (1.67), Nme1 (1.64), Nme2 (1.55), Hk2 (1.45), Gfpt1 (1.38), Phgdh (1.26). - Ak2 (0.89), Mdh1 (0.88), Galm (0.86), Tpi1 (0.86), Eno2 (0.86), Fh (0.86), Aldh9a1 (0.85), Mdh2 (0.84), Gcsh (0.83), Ldhb (0.82), Got2 (0.82), Pycr2 (0.82), Acat2 (0.82), Bckdha (0.82), Dlat (0.82), Pcyox1 (0.8), Tkt (0.8), Ogdh (0.79), Pfkp (0.79), Acaa1a (0.79), Sdha (0.79), Aco2 (0.78), Aldh7a1 (0.78), Idh3a (0.78), Hk1 (0.77), Dlst (0.77), Cs (0.77), Dld (0.76), Hsd17b10 (0.76), Pgm1 (0.74), Sdhb (0.74), Pdhb (0.73), Echs1 (0.72), Pfkm (0.71), Oat (0.71), Pcca (0.7), Hadhb (0.7), Idh1 (0.7), Hadha (0.69), Idh2 (0.69), Bcat2 (0.68), Ak3 (0.62), Acat1 (0.61), Hadh (0.6), Pccb (0.58), Aldh3a2 (0.58), Acaa2 (0.55), Aldh2 (0.54), Cat (0.47) |
| Valine, leucine and isoleucine degradation | - Bcat1 (1.94) - Aldh9a1 (0.85), Bckdha (0.82), Acat2 (0.82), Acaa1a (0.79), Aldh7A1 (0.78), Hsd17b10 (0.76), Mccc2 (0.76), Dld (0.76), Mccc1 (0.73), Acads (0.72), Echs1 (0.72), Hadhb (0.7), Pcca (0.7), Hadha (0.69), Bcat2 (0.68), Hibch (0.65), Acadsb (0.64), Hibadh (0.63), Acat1 (0.61), Ivd (0.61), Hadh (0.6), Aldh3a2 (0.58), Pccb (0.58), Aldh6A1 (0.55), Acaa2 (0.55), Aldh2 (0.54) |
| Carbon metabolism | - Psph (1.67), Hk2 (1.45), Phgdh (1.26); - Mdh1 (0.88), Tpi1 (0.86), Eno2 (0.86), Fh (0.86), Mdh2 (0.84), Acat2 (0.82), Got2 (0.82), Dlat (0.82), Tkt (0.8), Ogdh (0.79), Pfkp (0.79), Sdha (0.79), Aco2 (0.78), Idh3a (0.78), Hk1 (0.77), Dlst (0.77), Cs (0.77), Dld (0.76), Sdhb (0.74), Pdhb (0.73), Pc (0.73), Echs1 (0.72), Acads (0.72), Pfkm (0.71), Idh1 (0.7), Pcca (0.7), Hadha (0.69), Idh2 (0.69), Glud1 (0.66), Hibch (0.65), Acat1 (0.61), Pccb (0.58), Aldh6A1 (0.55), Cat (0.47) |
| Fatty acid degradation | - Acsl5 (1.29); - Aldh9a1 (0.85), Acat2 (0.82), Acaa1a (0.79), Aldh7A1 (0.78), Cpt1a (0.77), Acadvl (0.74), Acads (0.72), Echs1 (0.72), Acadl (0.7), Hadhb (0.7), Hadha (0.69), Eci1 (0.67), Cpt2 (0.67), Eci2 (0.65), Acadsb (0.64), Acat1 (0.61), Hadh (0.6), Aldh3a2 (0.58), Acaa2 (0.55), Aldh2 (0.54) |
| Citrate cycle (TCA cycle) | - Mdh1 (0.88), Fh (0.86), Mdh2 (0.84), Dlat (0.82), Ogdh (0.79), Sdha (0.79), Aco2 (0.78), Idh3a (0.78), Dlst (0.77), Cs (0.77), Dld (0.76), Sdhb (0.74), Pdhb (0.73), Pc (0.73), Idh1 (0.7), Idh2 (0.69) |
| Metabolic pathways  (involved highest number of proteins) | - Enpp1 (3.56), Bcat1 (1.94), Asns (1.85), Psph (1.67), Nme1 (1.64), Impdh2 (1.61), Ggt1 (1.58), Mthfd2l (1.56), Nme2 (1.55), Ugdh (1.46), Hk2 (1.45), Gfpt1 (1.38), Acsl5 (1.29), Fasn (1.26), Phgdh (1.26), Agps (1.21), Ckb (1.21); - Atp5c1 (0.89), Ak2 (0.89), Mdh1 (0.88), Gmps (0.88), Galm (0.86), Tpi1 (0.86), Fh (0.86), Eno2 (0.86), Thtpa (0.85), Aldh9a1 (0.85), Atp6v0a1 (0.85), Cmas (0.84), Mdh2 (0.84), Uqcrc1 (0.84), Hexa (0.84), P4ha1 (0.84), Gcsh (0.83), Prdx6 (0.83), Ldhb (0.82), Got2 (0.82), Pycr2 (0.82), Acat2 (0.82), Cmpk1 (0.82), Dlat (0.82), Impa1 (0.82), Atp5l (0.82), Bckdha (0.82), Dhfr (0.81), Gaa (0.81), Tkt (0.8), Atp5f1 (0.8), Ogdh (0.79), Galns (0.79), Atp5j (0.79), Pfkp (0.79), Dhcr7 (0.79), Acaa1a (0.79), Sdha (0.79), Idh3a (0.78), Aco2 (0.78), Aldh7A1 (0.78), Pcyt1a (0.77), Hk1 (0.77), Dlst (0.77), Cs (0.77), Atp5a1 (0.77), Dld (0.76), Hsd17b10 (0.76), Mccc2 (0.76), Atp5o (0.76), Alad (0.74), Pgm1 (0.74), Atp5b (0.74), Acadvl (0.74), Dhrs4 (0.74), Plcd1 (0.74), Sdhb (0.74), Pdhb (0.73), Pc (0.73), Mccc1 (0.73), Mpst (0.72), Atp5d (0.72), Acads (0.72), Echs1 (0.72), Cox4i1 (0.71), Pfkm (0.71), Oat (0.71), Sord (0.71), Pygb (0.71), Cox5a (0.7), Pcca (0.7), Hadhb (0.7), Idh1 (0.7), Aldh4a1 (0.7), Acadl (0.7), Fah (0.69), Hadha (0.69), Idh2 (0.69), Maoa (0.69), Uqcrq (0.69), Cox5b (0.68), Atp5j2 (0.68), Bcat2 (0.68), Hsd17b4 (0.67), Glud1 (0.66), Gusb (0.65), Hibch (0.65), Acadsb (0.64), Anpep (0.64), Hibadh (0.63), Aldh3b1 (0.62), Spr (0.61), Acat1 (0.61), Ivd (0.61), Hadh (0.6), Scp2 (0.59), Pccb (0.58), Aldh3a2 (0.58), Bdh2 (0.58), Acaa2 (0.55), Aldh6A1 (0.55), Aldh2 (0.54), Ugt1A6 (0.43), Aldh3a1 (0.22) |
| Fatty acid metabolism | - Acsl5 (1.29), Fasn (1.26); - Acat2 (0.82), Acaa1a (0.79), Cpt1a (0.77), Acadvl (0.74), Acads (0.72), Echs1 (0.72), Tecr (0.71), Acadl (0.7), Hadhb (0.7), Hadha (0.69), Cpt2 (0.67), Acadsb (0.64), Acat1 (0.61), Hadh (0.6), Acaa2 (0.55) |
| Pyruvate metabolism | - Mdh1 (0.88), Fh (0.86), Aldh9a1 (0.85), Mdh2 (0.84), Ldhb (0.82), Dlat (0.82), Acat2 (0.82), Aldh7A1 (0.78), Dld (0.76), Pdhb (0.73), Pc (0.73), Acat1 (0.61), Aldh3a2 (0.58), Aldh2 (0.54) |
| Glycolysis / Gluconeogenesis | - Hk2 (1.45); - Galm (0.86), Tpi1 (0.86), Eno2 (0.86), Aldh9a1 (0.85), Ldhb (0.82), Dlat (0.82), Pfkp (0.79), Aldh7A1 (0.78), Hk1 (0.77), Dld (0.76), Pgm1 (0.74), Pdhb (0.73), Pfkm (0.71), Aldh3b1 (0.62), Aldh3a2 (0.58), Aldh2 (0.54), Aldh3a1 (0.22) |
| Glyoxylate and dicarboxylate metabolism | - Mdh1 (0.88), Mdh2 (0.84), Gcsh (0.83), Acat2 (0.82), Aco2 (0.78), Cs (0.77), Dld (0.76), Pcca (0.7), Acat1 (0.61), Pccb (0.58), Cat (0.47) |
| Biosynthesis of amino acids | - Bcat1 (1.94), Psph (1.67), Phgdh (1.26); - Tpi1 (0.86), Eno2 0.86), Got2 (0.82), Pycr2 (0.82), Tkt (0.8), Pfkp (0.79), Aco2 (0.78), Idh3a (0.78), Aldh7A1 (0.78), Cs (0.77), Pc (0.73), Pfkm (0.71), Idh1 (0.7), Idh2 (0.69), Bcat2 (0.68) |
| Lysine degradation | - Plod2 (1.19); - Aldh9a1 (0.85), Acat2 (0.82), Ogdh (0.79), Aldh7A1 (0.78), Dlst (0.77), Echs1 (0.72), Hadha (0.69), Plod1 (0.69), Acat1 (0.61), Hadh (0.6), Aldh3a2 (0.58), Aldh2 (0.54) |
| Tryptophan metabolism | - Aldh9a1 (0.85), Acat2 (0.82), Ogdh (0.79), Aldh7a1 (0.78), Echs1 (0.72), Maoa (0.69), Hadha (0.69), Acat1 (0.61), Hadh (0.6), Aldh3a2 (0.58), Aldh2 (0.54), Cat (0.47) |
| 2-Oxocarboxylic acid metabolism | - Bcat1(1.94); - Got2(0.82), Aco2(0.78), Idh3a (0.78), Cs (0.77), Idh1(0.7), Idh2(0.69), Bcat2(0.68) |
| beta-Alanine metabolism | - Aldh9a1(0.85), Aldh7A1(0.78), Echs1(0.72), Hadha (0.69), Hibch (0.65), Aldh3b1(0.62), Aldh3a2(0.58), Aldh6a1(0.55), Aldh2(0.54), Aldh3a1(0.22) |
| Propanoate metabolism | - Ldhb (0.82), Acat2 (0.82), Echs1 (0.72), Pcca (0.7), Hadha (0.69), Hibch (0.65), Acat1 (0.61), Pccb (0.58), Aldh6a1 (0.55) |
| Arginine and proline metabolism | - Ckb (1.21); - Aldh9a1 (0.85), P4ha1 (0.84), Got2 (0.82), Pycr2 (0.82), Aldh7a1 (0.78), Oat (0.71), Aldh4a1 (0.7), Maoa (0.69), Aldh3a2(0.58), Aldh2(0.54) |
| Peroxisome | - Prdx5 (1.33), Acsl5 (1.29), Agps (1.21), Prdx1 (1.16); - Acaa1a (0.79), Crat (0.78), Dhrs4 (0.74), Idh1 (0.7), Idh2 (0.69), Hsd17b4 (0.67), Eci2 (0.65), Scp2 (0.59), Ech1 (0.5), Cat (0.47) |
| RNA transport | - Eif2S3 (1.45), Eif4a2 (1.4), Eif3j (1.36), Eef1a1 (1.33), Eif3h (1.28), Eif3a (1.27), Eif2b1 (1.25), Eif3c (1.24), Strap (1.22), Eif3i (1.21), Eif3d (1.18), Eif3b (1.17), Kpnb1 (1.14), Nup153 (1.13), Pabpc1 (1.13); - Tpr (0.91), Nup93 (0.82), Ube2i (0.8), Eif5 (0.79), Sumo2 (0.78) |
| Histidine metabolism | - Aldh9a1 (0.85), Aldh7a1 (0.78), Maoa (0.69), Aldh3b1 (0.62), Aldh3a2 (0.58), Aldh2 (0.54), Aldh3a1 (0.22) |
| Parkinson's disease | - Atp5c1 (0.89), Gnai2 (0.87), Uba1 (0.86), Uqcrc1 (0.84), Vdac1 (0.82), Atp5f1 (0.8), Park7 (0.8), Sdha (0.79), Atp5j (0.79), Atp5a1 (0.77), Atp5o (0.76), Atp5b (0.74), Sdhb (0.74), Atp5d (0.72), Cox4i1 (0.71), Cox5a (0.7), Uqcrq (0.69), Cox5b (0.68) |
| Butanoate metabolism | - Acat2 (0.82), Acads (0.72), Echs1 (0.72), Hadha (0.69), Acat1 (0.61), Hadh (0.6), Bdh2 (0.58) |
| Glutathione metabolism | - Ggt1 (1.58); - Oplah (0.79), Gpx1 (0.75), Gstm2 (0.74), Idh1 (0.7), Idh2 (0.69), Gstp1 (0.67), Anpep (0.64), Mgst1 (0.62), Gsta3 (0.59) |
| Oxidative phosphorylation | - Atp5c1 (0.89), Atp6v0a1 (0.85), Uqcrc1 (0.84), Atp5l (0.82), Atp5f1 (0.8), Sdha (0.79), Atp5j (0.79), Atp5a1 (0.77), Atp5o (0.76), Atp5b (0.74), Sdhb (0.74), Atp5d (0.72), Cox4i1 (0.71), Cox5a (0.7), Uqcrq (0.69), Atp5j2 (0.68), Cox5b (0.68) |
| Galactose metabolism | - Hk2 (1.45); - Galm (0.86), Gaa (0.81), Pfkp (0.79), Hk1 (0.77), Pgm1 (0.74), Pfkm (0.71) |
| Protein processing in endoplasmic reticulum | - Sec61a1 (1.61), Bax (1.38), Nsfl1C (1.33), Dnajc3 (1.33), Dnaja2 (1.31), Hsp90Ab1 (1.28), Hspa8 (1.26), Hsp90Aa1 (1.21), Hsph1 (1.21); - Rad23b (0.95), Dnajb11 (0.92), Vcp (0.9), Pdia6 (0.88), Pdia4 (0.82), Eif2ak2 (0.79), Sel1l (0.78), Capn1 (0.74), Os9 (0.51) |
| Alzheimer's disease | - Atp5c1 (0.89), Uqcrc1 (0.84), Mapk1 (0.84), Itpr3 (0.83), Atp5f1 (0.8), Sdha (0.79), Atp5j (0.79), Atp5a1 (0.77), Hsd17b10 (0.76), Atp5o (0.76), Atp5b (0.74), Capn1 (0.74), Sdhb (0.74), Atp5d (0.72), Cox4i1 (0.71), Cox5a (0.7), Uqcrq (0.69), Cox5b (0.68), Mapk3 (0.64) |
| Huntington's disease | - Bax (1.38); - Dctn1 (0.9), Atp5c1 (0.89), Ap2m1 (0.87), Hdac1 (0.85), Uqcrc1 (0.84), Clta (0.82), Vdac1 (0.82), Atp5f1 (0.8), Sdha (0.79), Atp5j (0.79), Atp5a1 (0.77), Atp5o (0.76), Atp5b (0.74), Sdhb (0.74), Atp5d (0.72), Cox4i1 (0.71), Cox5a (0.7), Uqcrq (0.69), Cox5b (0.68) |
| Amino sugar and nucleotide sugar metabolism | - Ugdh (1.46), Hk2 (1.45), Gfpt1 (1.38); - Cmas (0.84), Hexa (0.84), Cyb5r3 (0.81), Pgm1 (0.74), Hk1 (0.77) |
| Ascorbate and aldarate metabolism | - Ugdh (1.46); - Aldh9a1 (0.85), Aldh7a1 (0.78), Aldh3a2 (0.58), Aldh2 (0.54), Ugt1a6 (0.43) |
| Fatty acid elongation | - Echs1 (0.72), Tecr (0.71), Hadhb (0.70), Hadha (0.69), Hadh (0.60), Acaa2 (0.55) |
| Thyroid cancer | - Map2k1 (1.31), Tpm3 (1.12); - Tpr (0.91), Mapk1 (0.84), Mapk3 (0.64), Ctnnb1 (0.56) |
| Central carbon metabolism in cancer | - Slc7a5 (1.51), Hk2 (1.45), Map2k1 (1.31); - Mapk1 (0.84), Pfkp (0.79), Hk1 (0.77), Pdhb (0.73), Pfkm (0.71), Mapk3 (0.64) |
| Starch and sucrose metabolism | - Enpp1 (3.56), Hk2 (1.45); - Gaa (0.81), Hk1 (0.77), Pgm1 (0.74), Pygb (0.71) |
| Prion diseases | - Bax (1.38), Map2k1 (1.31), Stip1 (1.08); - Mapk1 (0.84), Mapk3 (0.64), Ncam1 (0.63) |
| Phenylalanine metabolism | - Got2 (0.82), Mif (0.74), Maoa (0.69), Aldh3b1 (0.62), Aldh3a1 (0.22) |
| Proteasome | - Psmb7 (1.45), Psmb6 (1.28), Psma4 (1.24), Psma1 (1.14); - Psmb10 (0.73), Psme1 (0.70), Psmb9 (0.70) |
| Pentose and glucuronate interconversions | - Ugdh (1.46); - Sord (0.71), Gusb (0.65), Aldh3a2 (0.58), Aldh2 (0.54), Ugt1A6 (0.43) |
| Fructose and mannose metabolism | - Hk2 (1.45); - Tpi1 (0.86), Pfkp (0.79), Hk1 (0.77), Sord (0.71), Pfkm (0.71) |
| Glycine, serine and threonine metabolism | - Psph (1.67), Phgdh (1.26); - Gcsh (0.83), Aldh7A1 (0.78), Dld (0.76), Maoa (0.69) |
| Tyrosine metabolism | - Got2 (0.82), Mif (0.74), Maoa (0.69), Fah (0.69), Aldh3b1 (0.62), Aldh3a1 (0.22) |

**Supplementary table 3. List of cell-lysate proteins in clusters a and b, Figure 4A.** Proteins are listed in order of TGF-β1/control ratios; **“•**”: induced by TGF-β1; **“•**”: repressed by TGF-β1**.**

| **Accession** | **Protein** | **Description** | **TGF-β1/ control** | **Cluster** |
| --- | --- | --- | --- | --- |
| Q62952 | - Dpysl3 | Dihydropyrimidinase-related protein 3 | 2.3262 | a |
| P30823 | - Slc7a1 | High affinity cationic amino acid transporter 1 | 2.0636 | a |
| Q63016 | - Slc7a5 | Large neutral amino acids transporter small subunit 1 | 1.5106 | a |
| P97852 | - Hsd17b4 | Peroxisomal multifunctional enzyme type 2 | 0.6697 | a |
| P10860 | - Glud1 | Glutamate dehydrogenase 1, mitochondrial | 0.6588 | a |
| P52759 | - Hrsp12 | Ribonuclease UK114 | 0.6538 | a |
| Q5XIC0 | - Eci2 | Enoyl-CoA delta isomerase 2, mitochondrial | 0.6525 | a |
| Q5XIE6 | - Hibch | 3-hydroxyisobutyryl-CoA hydrolase, mitochondrial | 0.6522 | a |
| P28037 | - Aldh1l1 | Cytosolic 10-formyltetrahydrofolate dehydrogenase | 0.6431 | a |
| P70584 | - Acadsb | Short/branched chain specific acyl-CoA dehydrogenase, mitochondrial | 0.6362 | a |
| P29266 | - Hibadh | 3-hydroxyisobutyrate dehydrogenase, mitochondrial | 0.6302 | a |
| Q5XI42 | - Aldh3b1 | Aldehyde dehydrogenase family 3 member B1 | 0.6198 | a |
| P12007 | - Ivd | Isovaleryl-CoA dehydrogenase, mitochondrial | 0.6106 | a |
| P17764 | - Acat1 | Acetyl-CoA acetyltransferase, mitochondrial | 0.606 | a |
| Q9WVK7 | - Hadh | Hydroxyacyl-coenzyme A dehydrogenase, mitochondrial | 0.5985 | a |
| P11915 | - Scp2 | Non-specific lipid-transfer protein | 0.5931 | a |
| P07633 | - Pccb | Propionyl-CoA carboxylase beta chain, mitochondrial | 0.582 | a |
| D4A1J4 | - Bdh2 | 3-hydroxybutyrate dehydrogenase type 2 | 0.5803 | a |
| P30839 | - Aldh3a2 | Fatty aldehyde dehydrogenase | 0.5781 | a |
| P13437 | - Acaa2 | 3-ketoacyl-CoA thiolase, mitochondrial | 0.5473 | a |
| Q02253 | - Aldh6a1 | Methylmalonate-semialdehyde dehydrogenase [acylating], mitochondrial | 0.5472 | a |
| P11884 | - Aldh2 | Aldehyde dehydrogenase, mitochondrial | 0.5406 | a |
| Q62651 | - Ech1 | Delta (3,5)-Delta (2,4)-dienoyl-CoA isomerase, mitochondrial | 0.4955 | a |
| Q64591 | - Decr1 | 2,4-dienoyl-CoA reductase, mitochondrial | 0.4932 | a |
| P04762 | - Cat | Catalase | 0.4743 | a |
| Q63028 | - Add1 | Alpha-adducin | 0.4357 | a |
| Q62847 | - Add3 | Gamma-adducin | 0.3743 | a |
| P11883 | - Aldh3a1 | Aldehyde dehydrogenase, dimeric NADP-preferring | 0.2221 | a |
| P62864 | - Rps30 | 40S ribosomal protein S30 | 2.0675 | b |
| P83883 | - Rpl36a | 60S ribosomal protein L36a | 1.9455 | b |
| P62268 | - Rps23 | 40S ribosomal protein S23 | 1.8894 | b |
| P04646 | - Rpl35a | 60S ribosomal protein L35a | 1.7115 | b |
| Q4KM49 | - Yars | Tyrosine-tRNA ligase, cytoplasmic | 1.7072 | b |
| Q5U2Q7 | - Etf1 | Eukaryotic peptide chain release factor subunit 1 | 1.7037 | b |
| Q05982 | - Nme1 | Nucleoside diphosphate kinase A | 1.6351 | b |
| P61621 | - Sec61a1 | Protein transport protein Sec61 subunit alpha isoform 1 | 1.6099 | b |
| P62850 | - Rps24 | 40S ribosomal protein S24 | 1.6045 | b |
| P61928 | - Rpl37 | 60S ribosomal protein L37 | 1.5624 | b |
| P62752 | - Rpl23a | 60S ribosomal protein L23a | 1.5556 | b |
| P19804 | - Nme2 | Nucleoside diphosphate kinase B | 1.5517 | b |
| P18445 | - Rpl27a | 60S ribosomal protein L27a | 1.5435 | b |
| Q6AYD3 | - Pa2g4 | Proliferation-associated protein 2G4 | 1.5241 | b |
| P62909 | - Rps3 | 40S ribosomal protein S3 | 1.5218 | b |
| Q794F9 | - Slc3a2 | 4F2 cell-surface antigen heavy chain | 1.5066 | b |
| P19945 | - Rplp0 | 60S acidic ribosomal protein P0 | 1.5003 | b |
| P29411 | - Ak3 | GTP: AMP phosphotransferase AK3, mitochondrial | 0.6212 | b |

Supplementary table 4. KEGG pathways and enriched cell-lysate proteins regulated by TGF-β1 in clusters a and **b, Figure 4A.** Proteins are listed in order of TGF-β1/control ratios; **“•**”: induced by TGF-β1; **“•**”: repressed by TGF-β1.

| **KEGG pathways** | **TGF-β1-regulated proteins (TGF-β1/control ratio)** |
| --- | --- |
| **Cluster a** | |
| Valine, leucine and isoleucine degradation | - Hibch (0.65), Acadsb (0.64), Hibadh (0.63), Ivd (0.61), Acat1 (0.61), Hadh (0.60), Pccb (0.58), Aldh3a2 (0.58), Acaa2 (0.55), Aldh6a1 (0.55), Aldh2 (0.52) |
| Fatty acid degradation | - Eci2 (0.65), Acadsb (0.64), Acat1(0.61), Hadh (0.60), Aldh3a2 (0.58), Acaa2 (0.55), Aldh2 (0.52) |
| Metabolic pathways | - Hsd17b4 (0.67), Glud1(0.66), Hibch (0.65), Acadsb (0.64), Hibadh (0.63), Aldh3b1(0.62), Ivd (0.61), Acat1(0.61), Hadh (0.60), Scp2 (0.59), Aldh3a2 (0.58), Bdh2 (0.58), Pccb (0.58), Acaa2 (0.55), Aldh6a1(0.55), Aldh2 (0.52), Aldh3a1(0.22) |
| beta-Alanine metabolism | - Hibch (0.65), Aldh3b1 (0.62), Aldh3a2 (0.58), Aldh6a1(0.55), Aldh2 (0.52), Aldh3a1 (0.22) |
| Tryptophan metabolism | - Acat1(0.61), Hadh (0.60), Aldh3a2 (0.58), Aldh2 (0.52), Cat (0.47) |
| Carbon metabolism | - Glud1(0.66), Hibch (0.65), Acat1(0.61), Pccb (0.58), Aldh6a1(0.55), Cat (0.47), |
| Biosynthesis of antibiotics | - Acaa2 (0.55), Aldh2 (0.52), Cat (0.47), Hadh (0.60), Acat1(0.61), Pccb (0.58), Aldh3a2 (0.58) |
| Histidine metabolism | - Aldh3b1 (0.62), Aldh3a2 (0.58), Aldh2 (0.52), Aldh3a1(0.22) |
| Propanoate metabolism | - Hibch (0.65), Acat1(0.61), Pccb (0.58), Aldh6a1(0.55) |
| Peroxisome | - Hsd17b4 (0.67), Eci2 (0.65), Scp2 (0.59), Ech1 (0.50), Cat (0.47) |
| Lysine degradation | - Acat1(0.61), Hadh (0.60), Aldh3a2 (0.58), Aldh2 (0.52) |
| Fatty acid metabolism | - Acadsb (0.64), Acat1(0.61), Hadh (0.60), Acaa2 (0.55) |
| Glycolysis / Gluconeogenesis | - Aldh3b1 (0.62), Aldh3a2 (0.58), Aldh2 (0.52), Aldh3a1 (0.22) |
| Glyoxylate and dicarboxylate metabolism | - Acat1(0.61), Pccb (0.58), Cat (0.47) |
| Butanoate metabolism | - Acat1(0.61), Hadh (0.60), Bdh2 (0.58) |
| Pyruvate metabolism | - Acat1(0.61), Aldh3a2 (0.58), Aldh2 (0.52) |
| Synthesis and degradation of ketone bodies | - Acat1(0.61), Bdh2 (0.58) |
| Primary bile acid biosynthesis | - Hsd17b4 (0.67), Scp2 (0.59) |
| **Cluster b** | |
| Ribosome | - Rps30 (2.11), Rpl36a (1.95), Rps23 (1.89), Rpl35a (1.71), Rps24 (1.60), Rpl23a (1.56), Rpl37 (1.56), Rpl27a (1.54), Rps3 (1.52), Rplp0 (1.50) |

**Supplementary table 5.** **List of all proteins in conditioned media significantly regulated by TGF-β1.** Proteins are listed in order of TGF-β1/control ratios; “•”: induced by TGF-β1; “•”: repressed by TGF-β1.

| **Accession** | **Gene Name** | **Protein Name** | **TGF-β1 /control** | **p-value** | **q-value** |
| --- | --- | --- | --- | --- | --- |
| P20961 | - Serpine1 (PAI-1) | Plasminogen activator inhibitor 1 | 4.41 | 0.0003 | 0.1494 |
| P14844 | - Ccl2 | C-C motif chemokine 2 | 3.47 | 0.0012 | 0.1996 |
| Q6QMY6 | - Tsku | Tsukushin | 2.60 | 0.0178 | 0.5190 |
| Q9QXY8 | - Ccl7 | C-C motif chemokine 7 | 2.43 | 0.0014 | 0.1996 |
| Q9ES72 | - Cyr61 | Protein CYR61 | 2.14 | 0.0082 | 0.3967 |
| Q9R0K8 | - Stc2 | Stanniocalcin-2 | 1.82 | 0.0360 | 0.5626 |
| Q811A3 | - Plod2 | Procollagen-lysine,2-oxoglutarate 5-dioxygenase 2 | 1.81 | 0.0005 | 0.1494 |
| O35806 | - Ltbp2 | Latent-transforming growth factor beta-binding protein 2 | 1.78 | 0.0310 | 0.5626 |
| Q9R1E9 | - Ctgf | Connective tissue growth factor | 1.73 | 0.0024 | 0.2467 |
| P17209 | - Myl4 | Myosin light chain 4 | 1.68 | 0.0027 | 0.2467 |
| Q9JI03 | - Col5a1 | Collagen alpha-1(V) chain 1 | 1.56 | 0.0442 | 0.5626 |
| P54690 | - Bcat1 | Branched-chain-amino-acid aminotransferase, cytosolic | 1.55 | 0.0243 | 0.5626 |
| Q9WV75 | - Spon2 | Spondin-2 | 1.54 | 0.0480 | 0.5780 |
| Q9Z0W7 | - Clic4 | Chloride intracellular channel protein 4 | 1.53 | 0.0379 | 0.5626 |
| Q62736 | - Cald1 | Non-muscle caldesmon | 1.49 | 0.0024 | 0.2467 |
| Q4V7E8 | - Lrrfip2 | Leucine-rich repeat flightless-interacting protein 2 | 1.49 | 0.0494 | 0.5780 |
| Q62908 | - Csrp2 | Cysteine and glycine-rich protein 2 | 1.46 | 0.0126 | 0.4562 |
| Q3B8Q1 | - Ddx21 | Nucleolar RNA helicase 2 | 1.35 | 0.0043 | 0.2558 |
| Q2LAP6 | - Tes | Testin | 1.27 | 0.0231 | 0.5577 |
| P39069 | - Ak1 | Adenylate kinase isoenzyme 1 | 1.27 | 0.0332 | 0.5626 |
| Q9Z2G8 | - Nap1l1 | Nucleosome assembly protein 1-like 1 | 1.25 | 0.0421 | 0.5626 |
| P23514 | - Copb1 | Coatomer subunit beta | 1.21 | 0.0468 | 0.5780 |
| P62752 | - Rpl23a | 60S ribosomal protein L23a | 1.19 | 0.0270 | 0.5626 |
| Q6AXS5 | - Serbp1 | Plasminogen activator inhibitor 1 RNA-binding protein | 1.15 | 0.0098 | 0.4431 |
| P13084 | - Npm1 | Nucleophosmin | 1.14 | 0.0325 | 0.5626 |
| O35217 | - Minpp1 | Multiple inositol polyphosphate phosphatase 1 | 0.85 | 0.0200 | 0.5190 |
| Q68FP1 | - Gsn | Gelsolin | 0.84 | 0.0122 | 0.4562 |
| Q4KM35 | - Psmb10 | Proteasome subunit beta type-10 | 0.82 | 0.0341 | 0.5626 |
| P28077 | - Psmb9 | Proteasome subunit beta type-9 | 0.8 | 0.0180 | 0.5190 |
| P70615 | - Lmnb1 | Lamin-B1 | 0.79 | 0.0250 | 0.5626 |
| Q6P7A9 | - Gaa | Lysosomal alpha-glucosidase | 0.78 | 0.0209 | 0.5233 |
| Q66H12 | - Naga | Alpha-N-acetylgalactosaminidase | 0.76 | 0.0276 | 0.5626 |
| Q6IRK9 | - Cpq | Carboxypeptidase Q | 0.75 | 0.0082 | 0.3967 |
| Q32KJ6 | - Galns | N-acetylgalactosamine-6-sulfatase | 0.7 | 0.0175 | 0.5190 |
| B0BNI5 | - Olfml3 | Olfactomedin-like protein 3 | 0.69 | 0.0435 | 0.5626 |
| Q6P502 | - Cct3 | T-complex protein 1 subunit gamma | 0.69 | 0.0041 | 0.2558 |
| P35053 | - Gpc1 | Glypican-1 | 0.68 | 0.0169 | 0.5190 |
| Q5U4F3 | - Fam107b | Protein FAM107B | 0.67 | 0.0107 | 0.4552 |
| P07154 | - Ctsl | Cathepsin L1 | 0.66 | 0.0420 | 0.5626 |
| P97546 | - Nptn | Neuroplastin | 0.63 | 0.0316 | 0.5626 |
| P17164 | - Fuca1 | Tissue alpha-L-fucosidase | 0.62 | 0.0006 | 0.1494 |
| P00787 | - Ctsb | Cathepsin B | 0.61 | 0.0046 | 0.2558 |
| P15087 | - Cpe | Carboxypeptidase E | 0.6 | 0.0286 | 0.5626 |
| P11883 | - Aldh3a1 | Aldehyde dehydrogenase, dimeric NADP-preferring | 0.59 | 0.0189 | 0.5190 |
| O35276 | - Nrp2 | Neuropilin-2 | 0.59 | 0.0034 | 0.2558 |
| P52303 | - Ap1b1 | AP-1 complex subunit beta-1 | 0.58 | 0.0198 | 0.5190 |
| P05371 | - Clu | Clusterin | 0.57 | 0.0342 | 0.5626 |
| P05964 | - S100a6 | Protein S100-A6 | 0.56 | 0.0394 | 0.5626 |
| P07092 | - Serpine2 | Glia-derived nexin | 0.56 | 0.0041 | 0.2558 |
| Q08420 | - Sod3 | Extracellular superoxide dismutase [Cu-Zn] | 0.55 | 0.0283 | 0.5626 |
| O55004 | - Rnase4 | Ribonuclease 4 | 0.54 | 0.0357 | 0.5626 |
| Q62894 | - Ecm1 | Extracellular matrix protein 1 | 0.53 | 0.0372 | 0.5626 |
| P01322 | - Ins1 | Insulin-1 | 0.52 | 0.0122 | 0.4562 |
| O70513 | - Lgals3bp | Galectin-3-binding protein | 0.50 | 0.0424 | 0.5626 |
| Q6P7C7 | - Gpnmb | Transmembrane glycoprotein NMB | 0.49 | 0.0180 | 0.5190 |
| P03957 | - Mmp3 | Stromelysin-1 | 0.43 | 0.0475 | 0.5780 |
| Q6IE64 | - C1rl | Complement C1r subcomponent-like protein | 0.41 | 0.0487 | 0.5780 |
| Q6P6T1 | - C1s | Complement C1s subcomponent | 0.39 | 0.0412 | 0.5626 |
| Q64610 | - Enpp2 | Ectonucleotide pyrophosphatase/phosphodiesterase family member 2 | 0.36 | 0.0435 | 0.5626 |
| Q63548 | - Sema3a | Semaphorin-3A | 0.35 | 0.0412 | 0.5626 |
| B5DFC9 | - Nid2 | Nidogen-2 | 0.34 | 0.0393 | 0.5626 |
| Q9QZQ5 | - Nov | Protein NOV homolog | 0.28 | 0.0339 | 0.5626 |

Supplementary table 6. GO cellular component analysis of proteins in conditioned media significantly regulated by TGF-β1. Proteins are listed in order of TGF-β1/control ratios; **“•**”: induced by TGF-β1; **“•**”: repressed by TGF-β1**.**

| **GO cellular components** | **Protein (TGF-β1/control ratio)** |
| --- | --- |
| Extracellular exosome | - PAI-1(4.41), Plod2 (1.81), Ltbp2 (1.78), Col5a1 (1.56), Spon2 (1.54), Clic4 (1.53), Ak1 (1.27), Rpl23a (1.19), Serbp1 (1.15) |
|  | - Nid2 (0.34), C1s (0.39), C1rl (0.41), Lgals3bp (0.50), Ecm1 (0.53), Rnase4 (0.54), Sod3 (0.55), S100a6 (0.56), Clu (0.57), Cpe (0.60), Ctsb (0.61), Fuca1 (0.62), Ctsl (0.66), Gpc1 (0.68), Cct3 (0.69), Galns (0.70), Cpq (0.75), Naga (0.76), Gaa (0.78), Psmb9 (0.80), Gsn (0.84), Minpp1 (0.85) |
| Cytoplasm | - PAI-1(4.41), Ccl2 (3.47), Bcat1 (1.55), Clic4 (1.53), Tes (1.27), Ak1 (1.27), Nap1l1 (1.25), Copb1 (1.21), Rpl23a (1.19), Serbp1 (1.15), Npm1 (1.14) |
|  | - Nov (0.28), Enpp2 (0.36), Ins1 (0.52), Sod3 (0.55), S100a6 (0.56), Clu (0.57), Aldh3a1 (0.59), Ctsb (0.61), Ctsl (0.66), Cct3 (0.69), Cpq (0.75), Naga (0.76), Lmnb1 (0.79), Psmb9 (0.80), Psmb10 (0.82), Gsn (0.84) |
| Extracellular space | - PAI-1(4.41), Ccl2 (3.47), Tsku (2.60), Ccl7 (2.43), Stc2 (1.82), Ltbp2 (1.78), Ctgf (1.73), Spon2 (1.54) |
|  | - Sema3a (0.35), Enpp2 (0.36), C1rl (0.41), Mmp3 (0.43), Lgals3bp (0.50), Ins1 (0.52), Ecm1 (0.53), Sod3 (0.55), Serpine2 (0.56), Clu (0.57), Aldh3a1 (0.59), Cpe (0.60), Ctsb (0.61), Ctsl (0.66), Gpc1 (0.68), Cpq (0.75), Gsn (0.84) |
| Extracellular region | - PAI-1(4.41), Ccl2 (3.47), Tsku (2.60), Cyr61 (2.14), Ctgf (1.73) |
|  | - Nov (0.28), C1s (0.39), C1rl (0.41), Rnase4 (0.54), Serpine2 (0.56), Clu (0.57), Cpe (0.60), Ctsb (0.61), Olfml3 (0.69), Gsn (0.84) |
| Extracellular matrix | - PAI-1(4.41), Cyr61 (2.14), Col5a1 (1.56), Spon2 (1.54), Ltbp2 (1.78) |
|  | - Nov (0.28), Nid2 (0.34), Mmp3 (0.43), Lgals3bp (0.50), Ecm1 (0.53), Sod3 (0.55), Serpine2 (0.56), Clu (0.57) |
| Cytosol | - Clic4 (1.53), Ctgf (1.73), Bcat1 (1.55), Npm1 (1.14) |
|  | - Mmp3 (0.43), Ins1 (0.52), S100a6 (0.56), Serpine2 (0.56), Clu (0.57), Aldh3a1 (0.59), Psmb9 (0.80), Gsn (0.84) |
| Proteinaceous extracellular matrix | - Cyr61 (2.14), Ltbp2 (1.78), Ctgf (1.73), Col5a1 (1.56), Spon2 (1.54) |
|  | - Nov (0.28), Nid2 (0.34), Mmp3 (0.43), Lgals3bp (0.50), Ecm1 (0.53), Gpc1 (0.68) |
| Perinuclear region of cytoplasm | - Ccl2 (3.47), Stc2 (1.82), Ctgf (1.73), Clic4 (1.53), Ak1 (1.27), Serbp1 (1.15) |
|  | - S100a6 (0.56), Clu (0.57), Ctsb (0.61), Gsn (0.84) |
| Lysosome | - Ctsb (0.61), Fuca1 (0.62), Ctsl (0.66), Galns (0.70), Cpq (0.75), Naga (0.76), Gaa (0.78) |
| Dendrite | - Ccl2 (3.47), Cald1 (1.49) |
|  | - Nov (0.28), Sema3a (0.35), Mmp3 (0.43), Cpe (0.60), Nptn (0.63) |
| Endoplasmic reticulum | - Stc2 (1.82), Plod2 (1.81), Clic4 (1.53), Copb1 (1.21) |
|  | - Clu (0.57), Aldh3a1 (0.59), Cpq (0.75) |
| Golgi apparatus | - Stc2 (1.82), Ctgf (1.73), Copb1 (1.21) |
|  | - Enpp2 (0.36), Ap1b1 (0.58), Cpe (0.60), Cpq (0.75) |
| Neuronal cell body | - Ccl2 (3.47), Cald1 (1.49) |
|  | - Nov (0.28), Serpine2 (0.56), Cpe (0.60), Gpc1 (0.68) |
| Blood microparticle | - Gsn (0.84), Clu (0.57), Lgals3bp (0.50), C1s (0.39) |
| Melanosome | - Nap1l1 (1.25) |
|  | - Gpnmb (0.49), Ctsb (0.61) |
| Nuclear matrix | - Clic4 (1.53), Npm1 (1.14) |
|  | - Lmnb1 (0.79) |
| Secretory granule | - Ctsl (0.66), Cpe (0.60), Ins1 (0.52) |
| Chromaffin granule | - PAI-1(4.41) |
|  | - Clu (0.57) |
| Actin cap | - Cald1 (1.49) |
|  | - Gsn (0.84) |
| Spermatoproteasome complex | - Psmb10 (0.82), Psmb9 (0.80) |

Supplementary table 7. GO biological process analysis of proteins in conditioned media significantly regulated by TGF-β1. Proteins are listed in order of TGF-β1/control ratios; **“•**”: induced by TGF-β1; **“•**”: repressed by TGF-β1)**.**

| **GO biological processes** | **Protein (TGF-β1/control ratio)** |
| --- | --- |
| Cell adhesion | - Cyr61 (2.14), Ctgf (1.73), Col5a1 (1.56), Spon2 (1.54) - Nov (0.28), Gpnmb (0.49), Lgals3bp (0.50) |
| Angiogenesis | - PAI-1(4.41), Ctgf (1.73), Clic4 (1.53) - Nov (0.28), Ecm1 (0.53), Nrp2 (0.59) |
| Proteolysis | - C1s (0.39), C1rl (0.41), Mmp3 (0.43), Ctsb (0.61), Ctsl (0.66), Cpq (0.75) |
| Cellular response to interleukin-1 | - PAI-1(4.41), Ccl2 (3.47), Ccl7 (2.43) - Mmp3 (0.43), Psmb9 (0.80) |
| Positive regulation of ERK1 and ERK2 cascade | - Ccl2 (3.47), Ccl7 (2.43), Ctgf (1.73) - Gpnmb (0.49), Nptn (0.63) |
| Response to hypoxia | - Ccl2 (3.47), Plod2 (1.81) - Mmp3 (0.43), Sod3 (0.55), Aldh3a1 (0.59) |
| Ageing | - Ccl2 (3.47), Ctgf (1.73) - Clu (0.57), Aldh3a1 (0.59), Gsn (0.84) |
| Decidualization | - PAI-1(4.41), Stc2 (1.82) - Ctsb (0.61), Ctsl (0.66) |
| Proteolysis involved in cellular protein catabolic process | - Psmb10 (0.82), Psmb9 (0.80), Ctsl (0.66), Ctsb (0.61) |
| Response to wounding | - Ccl2 (3.47) - Serpine2 (0.56), Clu (0.57), Ctsb (0.61) |
| Negative regulation of cell death | - Cyr61 (2.14), Ctgf (1.73) - Nov (0.28), Ctsb (0.61) |
| Response to cytokine | - PAI-1(4.41) - Mmp3 (0.43), Ins1 (0.52), Ctsb (0.61) |
| Response to peptide hormone | - Stc2 (1.82), Ctgf (1.73) - Ins1 (0.52), Ctsb (0.61) |
| Positive regulation of protein phosphorylation | - Cyr61 (2.14), Ctgf (1.73) - Gpnmb (0.49), Nptn (0.63) |
| Negative regulation of gene expression | - PAI-1(4.41), Stc2 (1.82), Ctgf (1.73), Npm1 (1.14) |
| Nerve development | - Sema3a (0.35), Nrp2 (0.59), Ctsl (0.66) |
| Collagen catabolic process | - Mmp3 (0.43), Ctsb (0.61), Ctsl (0.66) |
| Tissue regeneration | - PAI-1(4.41) - Cpq (0.75), Gsn (0.84) |
| Positive regulation of cell differentiation | - Cyr61 (2.14), Ctgf (1.73) - Clu (0.57) |
| Positive regulation of tumuor necrosis factor production | - Ccl2 (3.47), Spon2 (1.54) - Clu (0.57) |
| Response to amino acid | - Ccl2 (3.47), Ctgf (1.73) - Mmp3 (0.43) |
| Positive regulation of inflammatory response | - PAI-1(4.41), Ccl2 (3.47), Ccl7 (2.43) |
| Response to cAMP | - Aldh3a1 (0.59), Ins1 (0.52), C1s (0.39) |
| Cell-cell signalling | - Cyr61 (2.14), Ctgf (1.73) - Nov (0.28) |
| Facioacoustic ganglion development | - Nrp2 (0.59), Sema3a (0.35) |
| Gonadotrophin-releasing hormone neuronal migration to the hypothalamus | - Nrp2 (0.59), Sema3a (0.35) |
| Semaphorin-plexin signalling pathway involved in neuron projection guidance | - Nrp2 (0.59), Sema3a (0.35) |
| Sympathetic neuron projection extension | - Nrp2 (0.59), Sema3a (0.35) |
| Ventral trunk neural crest cell migration | - Nrp2 (0.59), Sema3a (0.35) |
| Sympathetic neuron projection guidance | - Nrp2 (0.59), Sema3a (0.35) |
| Negative regulation of plasminogen activation | - PAI-1(4.41) - Serpine2 (0.56) |
| Trigeminal ganglion development | - Nrp2 (0.59), Sema3a (0.35) |
| Neural crest cell migration involved in autonomic nervous system development | - Nrp2 (0.59), Sema3a (0.35) |
| Glycoside catabolic process | - Naga (0.76), Fuca1 (0.62) |
| Sympathetic ganglion development | - Nrp2 (0.59), Sema3a (0.35) |
| Facial nerve structural organization | - Nrp2 (0.59), Sema3a (0.35) |
| Extracellular fibril organization | - Ltbp2 (1.78), Col5a1 (1.56) |
| Cellular response to ATP | - PAI-1(4.41), Ccl2 (3.47) |
| Response to transforming growth factor beta | - PAI-1(4.41), Cald1 (1.49) |
| Axon extension involved in axon guidance | - Nrp2 (0.59), Sema3a (0.35) |

Supplementary table 8. GO molecular function analysis of proteins in conditioned media significantly regulated by TGF-β1. Proteins are listed in order of TGF-β1/control ratios; **“•**”: induced by TGF-β1; **“•**”: repressed by TGF-β1)**.**

| **GO molecular functions** | **Protein (TGF-β1/ control ratio)** |
| --- | --- |
| Heparin binding | - Ccl2 (3.47), Ccl7 (2.43), Cyr61 (2.14), Ltbp2 (1.78), Ctgf (1.73), Col5a1 (1.56); - Nov (0.28), Gpnmb (0.49), Serpine2 (0.56), Nrp2 (0.59) |
| Calcium ion binding | - Ltbp2 (1.78), Myl4 (1.68); - Nid2 (0.34), Enpp2 (0.36), C1s (0.39), Mmp3 (0.43), S100a6 (0.56), Gsn (0.84) |
| Integrin binding | - Cyr61 (2.14), Ctgf (1.73); - Nov (0.28), Gpnmb (0.49) |
| Proteoglycan binding | - Col5a1 (1.56); - Ctsb (0.61), Ctsl (0.66) |
| Insulin-like growth factor binding | - Cyr61 (2.14), Ctgf (1.73); - Nov (0.28) |
| Kininogen binding | - Ctsb (0.61), Ctsl (0.66); |
| CCR2 chemokine receptor binding | - Ccl2 (3.47), Ccl7 (2.43) |

**Supplementary table 9.** A list of ELISA kits used in this project.

| **ELISA kits** | **Suppliers** |
| --- | --- |
| Protein Nov (Ccn3), ab205570 | Abcam (Cambridge, UK) |
| Plasminogen activator inhibitor 1 (PAI-1/Serpine1), ab201283 |  |
| Connective Tissue Growth Factor (Ctgf), MBS261004 | MyBiosource (San Diego, USA) |
| Matrix Metalloproteinase 3 (Mmp3), MBS762109 |  |
| Procollagen Lysine-2-Oxoglutarate-5-Dioxygenase 2 (Plod2), MBS9391878 |  |
| C-C motif chemokine 2 (Ccl2), MBS824584 |  |
| C-C motif chemokine 7 (Ccl7), MBS8244676 |  |
| Protein Cyr61, MBS9425693 |  |
| Inosine-5'-monophosphate dehydrogenase 2 (Impdh2), XPER1807 | Express biotech International (Frederick, USA) |
| Ectonucleotide pyrophosphatase/phosphodiesterase family member 1 (Enpp1), ER0419 | Fine Biotech (Wuhan, China) |
| Transferrin receptor (Tfrc), ER1375 |  |
| Aldehyde dehydrogenase, dimeric NADP-preferring (Aldh3a1), E02A1034 | BlueGene Biotech (Shanghai, China) |
| Coiled-coil domain-containing protein 80 (Ccdc80), E02C1111 |  |
| Tsukushin (Tsku), E02T0739 |  |
| Sequestosome-1 (Sqstm1), E12411744 | Sincere Biotech (Beijing, China) |

.
